# Solvent-free hydroboration of alkynes catalyzed by an NHC–cobalt complex†

**DOI:** 10.1039/d2ra03005e

**Published:** 2022-06-27

**Authors:** Małgorzata Bołt, Patrycja Żak

**Affiliations:** Department of Organometallic Chemistry, Faculty of Chemistry, Adam Mickiewicz University in Poznan Uniwersytetu Poznańskiego 8 61-614 Poznan Poland pkw@amu.edu.pl

## Abstract

A new cobalt complex bearing a bulky N-heterocyclic carbene (NHC) ligand is described as a pre-catalyst for alkyne hydroboration. The proposed catalytic system, synthesized using easily accessible reagents, allowed obtaining a series of mono- and dialkenylboranes in solvent-free conditions with excellent efficiency and selectivity. The results have been compared to those obtained in the presence of the same cobalt complex containing smaller NHC ligands and those achieved for the catalytic system based on a CoCl_2_ – NHC precursor.

## Abstract 2

Cobalt complex bearing bulky NHC ligand enable selective hydroboration of alkynes in solvent-free conditions.

## Graphical abstract

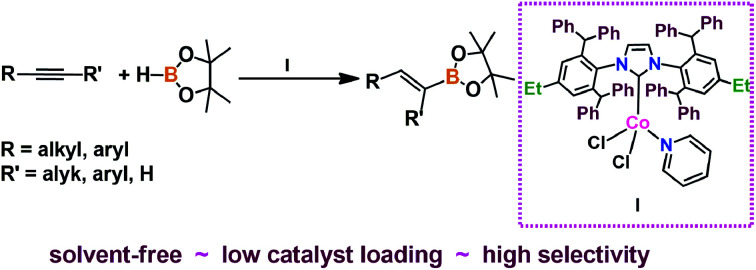

## 1 Introduction

Alkenylboronate esters are important and valuable compounds in modern organic chemistry.^1–5^ They make a group of useful building-blocks in many transformations among which the Suzuki–Miyaura cross-coupling is the most significant and widely known.^6–8^ Synthesis of alkenylboronic acid derivatives can be performed by the reactions of lithium or Grignard reagents with alkylboranes, however, these reactions involve the use of toxic and corrosive substances.^9–11^ Recently, hydroboration of alkynes has become the most important method of obtaining these compounds.^12,13^ B–H addition to alkene and alkynes can be catalyzed by the main group elements, such as sodium,^14^ lithium,^15^ magnesium,^16^ aluminium^17,18^ and boron.^19,20^ Precious metal-based systems are commonly used in transformations of this type, however, a number of earth abundant metal catalysts have been developed and proved to be active in this reaction as well.^21–23^ Cobalt and iron have been of particular interest, but most of the examples known in this field include pincer ligands.^24–29^ Lu *et al.* have applied a cobalt(ii) complex bearing the chiral imidazoline iminopyridine (IIP) ligand in sequential hydroboration/hydrogenation of internal alkynes,^30^ though, the substrate scope in the method is limited to propargyl ethers. Most recently the same group have described Markovnikov-selective terminal alkyne hydroboration using cobalt(ii) acetate and *N*-(oxazolinylphenyl)quinoline-2-carboxamide as a ligand.^31^ The reaction runs smoothly for a number of terminal alkynes but 5 mol% of cobalt salt is needed for the effective process. In 2017 Zheng *et al.* devised a cobalt(ii) coordination polymer active in alkyne hydroboration characterized by very high TOF values, but the final yield of the products strongly depended on the substituents.^32^ In 2021 Breit *et al.* published a successful cobalt-catalyzed hydroboration for a wide scope of alkynes in the presence of phosphine ligands.^33^

Among the most recently published methods leading to β-(*E*)-products of terminal alkyne hydroboration we can mention protocols based on transition metals such as: iron,^34^ cobalt,^32,35,36^ cooper,^37^ zirconium^38^ and silver.^39^ What is more, several processes catalyzed by main-group elements^16–20^ can lead to anti-Markovnikov products. However, they still suffer from some disadvantages such as high catalyst loading, elevated temperature and limited substrate scope.

To the best of our knowledge, there is only one example of a cobalt complex bearing an NHC ligand that proved to be active in hydroboration. Co(Mes)_2_Cl was applied in Markovnikov-selective alkene hydroboration, although mixtures of products were obtained for a bunch of examples.^40^ The same group has published also effective borylation of aryl and alkyl halides in the presence of NHC cobalt complexes.^41,42^

Herein, we report the application of a new cobalt complex bearing a bulky NHC carbene ligand in selective formation of mono- and dialkenylboranes with *E* geometry around a newly formed double bond.

## 2 Results and discussion

We began our research by examining the reaction of 4-ethynyltoluene (1a) with pinacolborane (2) in the presence of CoCl_2_ with different types of NHC precursors (Table 1). Mixing 2 mol% of a known and commercially available IMes precursor (A) with 1 mol% of cobalt(ii) chloride and 4 mol% of KO*t*Bu^43^ led to 74% conversion of alkyne. GC-MS and ^1^H NMR analyses confirmed the formation of *E* and *Z* isomers in a ratio of 82 : 18 (Table 1, entry 1). More sterically crowded imidazolium salt – Dipp (B) showed comparable activity and selectivity (Table 1, entry 2). Application of triazolium salt (C) gave a lower yield of the product without a meaningful change in the isomers ratio in the post-reaction mixture (Table 1, entry 3). The choice of a superbulky NHC salt (D) allowed the highest conversion and selectivity from among all tested ligand precursors (Table 1, entry 4). The use of cobalt chloride in the absence of any ligand allowed a higher conversion of alkyne, however, what is important, in the post-reaction mixture, apart from hydroboration products, we observed products of acetylene trimerization (Table 1, entry 5). We decided to test *in situ* generated catalytic system with ligand D with different types of bases. Application of KHMDS allowed a higher alkyne conversion but with lower selectivity (Table 1, entry 6). Weaker bases, such as potassium and cesium carbonates (Table 1, entries 7 and 8) caused a significant conversion lowering. It is worth mentioning that in all these cases we observed a significant amount of trimerization products. A test without the use of any base confirmed that the base presence is necessary for a successful reaction run (Table 1, entry 9).

**Table 1:** Hydroboration of 4-ethynyltoluene (1a) with pinacolborane (2) catalyzed by CoCl_2_/NHC. Optimization of reaction conditions^a^

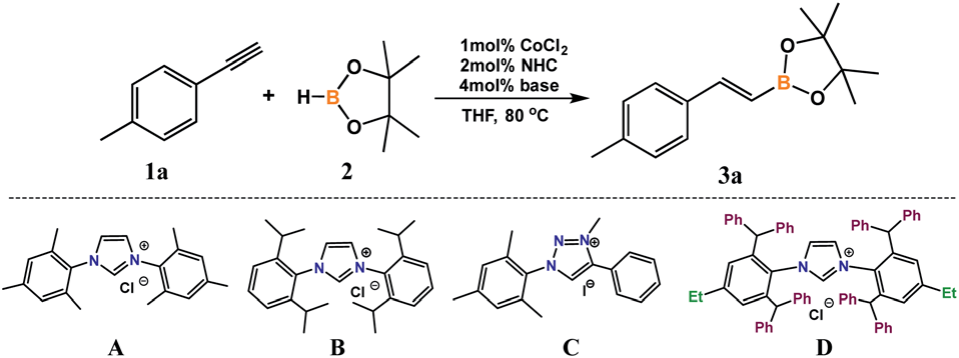					
Entry	NHC	Base	Conv. of 1a^d^ [%]	Yield of 3a^e^ [%]	(*E*) : (*Z*)^e^
1	A	KO*t*Bu	74	74	82 : 18
2	B	KO*t*Bu	68	68	87 : 13
3	C	KO*t*Bu	48	48	85 : 15
4	D	KO*t*Bu	79	79	91 : 9
5	—	KO*t*Bu	86	50^c^	98 : 02
6^b^	D	KHMDS	88	30^c^	83 : 17
7	D	K_2_CO_3_	48	45^c^	87 : 13
8	D	Cs_2_CO_3_	57	40^c^	86 : 14
9	D	—	30	30	97 : 3

a [1a] : [2] = 1 : 1.2, [Co] = 1 mol%, [NHC] = 2 mol%, [base] = 4 mol%, 3–24 h, THF, 80 °C, argon.

b Toluene was used as solvent.

c Products of trimerization of 1a were detected along with hydroboration products.

d Determined by GC-MS analysis using dodecane as an internal standard.

e Determined by GC-MS analysis and confirmed by ^1^HNMR spectroscopy of the crude reaction mixture.

**Scheme 1:**
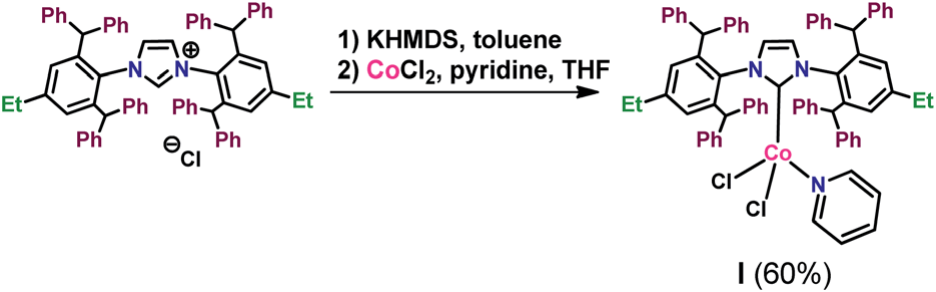
Synthesis of cobalt complex I.

Encouraged by the positive results, we made attempts at isolation of the complex generated in the processes occurring in the presence of D. For this purpose we carried out a reaction between anhydrous cobalt(ii) chloride and freshly prepared carbene solution, according to the procedure described earlier by Matsubara.^44^ We additionally used pyridine as a 2e-donor ligand to stabilize the obtained complex (Scheme 1):

Resulting complex I was isolated as blue solid in a yield of 60%. It was not air stable and had to be stored under inert gas. It was insoluble in methanol, n-hexane, pentane, but it was well-soluble in chloroform, dichloromethane and toluene. Due to the paramagnetic character of I, we were able to observe broad signals in the ^1^H NMR spectrum, which are consistent with its proposed structure. Additional HSQC correlation spectrum was recorded, which confirmed the presence of signals from CH_2_ at 1.99 ppm and from CH_3_ at 0.59 ppm, corresponding to the NHC ligand ethyl groups (see ESI†). MALDI-TOF analysis confirmed the presence of a compound with a mass of 1172.61, which corresponds to the mass of the proposed complex I increased by the mass of sodium.

The synthesized cobalt complex I was tested in the hydroboration of alkynes. Preliminary test was performed in the conditions optimized for the above-described system in which complex I was generated *in situ* in the reaction conditions (Table 1, entry 4). The addition of 1 mol% of complex I followed by 1 mol% of KO *t*Bu to the reaction mixture at 80 °C, resulted in full conversion of alkyne after 8 hours and the formation of a single product, which was identified by GC-MS and ^1^H NMR spectroscopy as (*E*)-2-(4-methylphenyl)-vinylboronic acid pinacol ester (3a). This positive result encouraged us to undertake further investigation. We conducted a series of optimization tests to determine the effect of solvent, temperature, type of base as well as type and concentration of the pre-catalyst. The results are collected in Table 2.

**Table 2:** Hydroboration of 4-ethynyltoluene (1a) with pinacolborane (2) catalyzed by I. Optimization of reaction conditions^a^

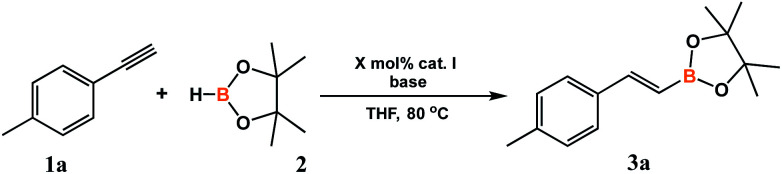					
Entry	Cat.	[Co] (mol%)	Base	Conv. of 1a^c^ [%]	(*E*) : (*Z*)^d^
1		10^–1^	LiHBEt_3_	80	99 : 1
2		5 × 10^−1^	LiHBEt_3_	98	99 : 1
3		10^0^	LiHBEt_3_	100	99 : 1
4^b^		5 × 10^−1^	LiHBEt_3_	78	99 : 1
5	I	5 × 10^−1^	NaHBEt_3_	94	97 : 3
6		5 × 10^−1^	KO*t*Bu	82	99 : 1
7		5 × 10^−1^	KHMDS	93	81 : 19
8		5 × 10^−1^	K_2_CO_3_	67	78 : 22
9		5 × 10^−1^	—	<5	—
10	II	5 × 10^−1^	LiHBEt_3_	78	91 : 3
11	III	5 × 10^−1^	LiHBEt_3_	82	97 : 3
12	—	5 × 10^−1^	LiHBEt_3_	<5	—

a [1a]: [2] = 1 : 1.2, [base]: [Co] = 2 : 1, I – [(IPr*^Et^)Co(py)Cl_2_], II - [(IMes)Co(py)Cl_2_], III - [(IPr)Co(py)Cl_2_], 8–24 h, THF, 80 °C, argon.

b THF, 60 °C.

c Determined by GC analysis using dodecane as an internal standard.

d Determined by GC-MS analysis and confirmed by ^1^HNMR spectroscopy of the crude reaction mixture.

As indicated in Table 2, a nearly complete conversion of alkyne can be obtained in the presence of 0.5 mol% of catalyst I (Table 2, entry 2). A slight decrease in the conversion of the reactants was noted when the catalyst concentration was reduced to 0.1 mol% (Table 2, entry 1). Also, lowering the temperature to 60 °C resulted in a slightly lower conversion (Table 2, entry 4). Several bases were tested as pre-catalyst activators, of which LiHBEt_3_ was shown to give the best results. Almost the same effect was observed with NaHBEt_3_ (Table 2, entry 5).^45^ The use of KO*t*Bu reduced the alkyne conversion to 82%, while the selectivity remained unchanged (Table 2, entry 6). The use of K_2_CO_3_ or KHMDS as the activator, led to obtaining a mixture of products (Table 2, entries 7 and 8). Carrying out the reaction without addition of a base led to a trace degree of conversion, which confirmed that a base is indispensable for effective reaction course (Table 2, entry 9). Finally, we compared the catalytic performance of catalyst I with that of its analogue bearing an NHC ligand causing smaller steric hindrance (Table 2, entries 10 and 11). While the selectivity of this reactions was only slightly worse, the activity of complexes II proved to be lower and led to only 78–82% of alkyne conversion. Finally, the activity of 1 mol% of LiHBEt_3_ without the cobalt catalyst was tested, but this reaction led to traces of desired products (Table 2, entry 12). Although it has been shown that the bases used by us as activators can act as hidden catalysts,^46,47^ the control test with 1 mol% of LiHBEt_3_ revealed that this amount was insufficient to achieve a satisfying efficiency of the reaction. Hence, the necessity of the base presence is rather connected with the reduction of cobalt and generation of the active catalyst. The reaction studied was optimized as to the amount and type of solvent. We discovered that the process can be carried out under solvent-free conditions, which is very attractive for economic and ecological reasons, however, toluene can be successfully used as the reaction medium without loss of selectivity or reactants conversion. The efficiency of the process was independent of the amount of solvent used. Therefore, when using both solid reagents, the minimum amount of solvents can be used.

**Scheme 2:**
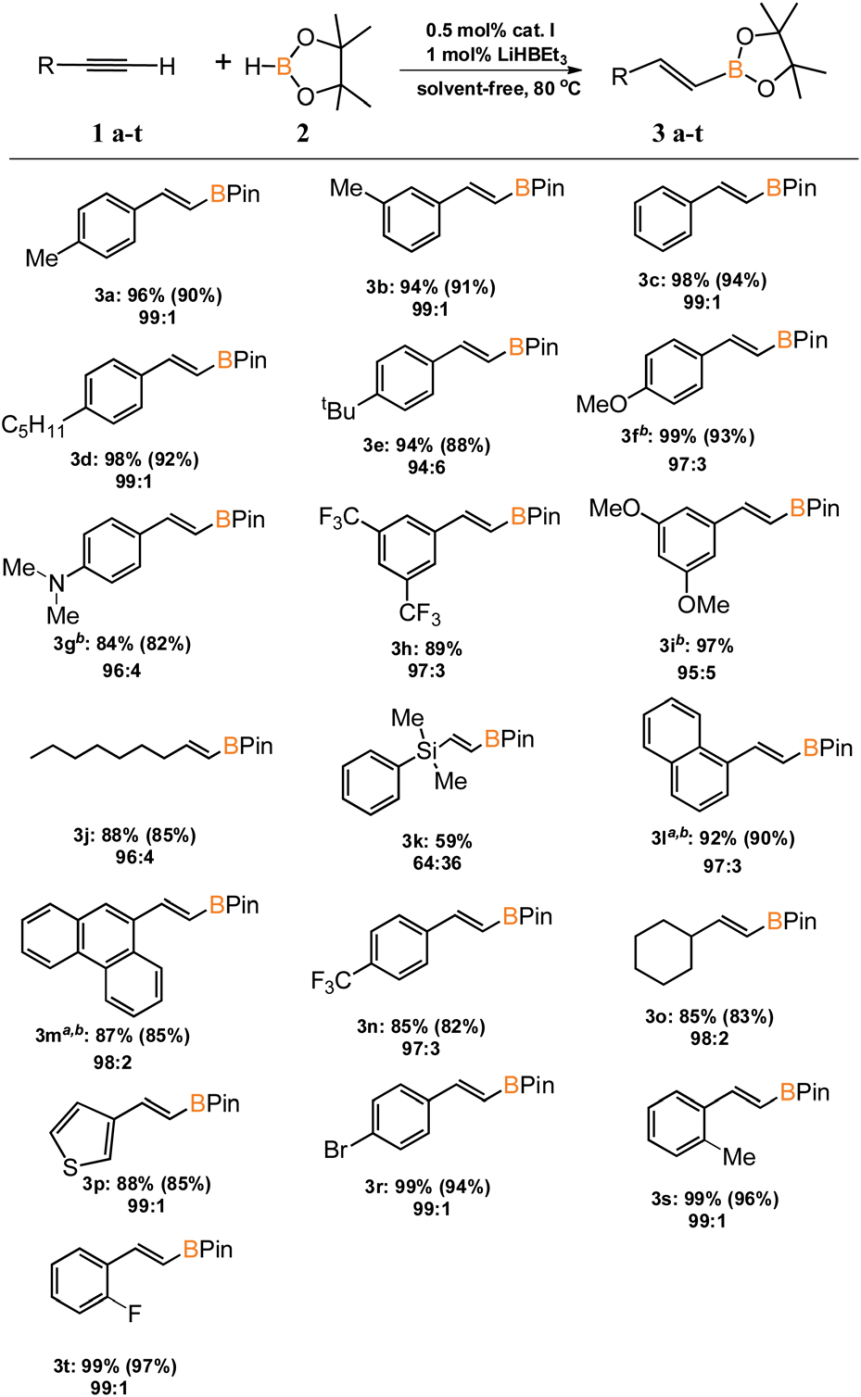
Substrate scope. Conversion of alkynes and selectivity β- *E*:β-*Z* are given under the product structure. Isolated yields are given in parentheses. ^*a*^ 1 mol% of catalyst I was used. ^*b*^ 0.2 mL of toluene was used because the reactants were in solid state.

With an active and selective catalytic system in hand, the range of substrates was extended to determine the versatility of the method. Several commercially available alkynes with alkyl, aryl and silyl substituents were tested in the optimized reaction conditions. For most of the tested substrates, nearly quantitative yields and exclusive formation of β-*E* product was detected (Scheme 2). No meaningful difference in the efficiency and selectivity of process for aryl substituted acetylenes was noted, except for 1-ethynylnaphthalene (3l) and 9-ethynylphenanthrene (3m). For these sterically crowded substrates, a higher catalyst loading was indispensable to achieve satisfactory results. Hydroboration of alkyl substituted alkyne gave the expected product with good yield and selectivity. Only for (dimethylphenylsilyl)acetylene (3k), precatalyst I showed mediocre activity and the reaction gave a mixture of β-*E* and β-*Z* products in the ratio of 64 to 36. We isolated all products in order to develop a universal method for their separation from the reaction mixture.

**Scheme 3:**
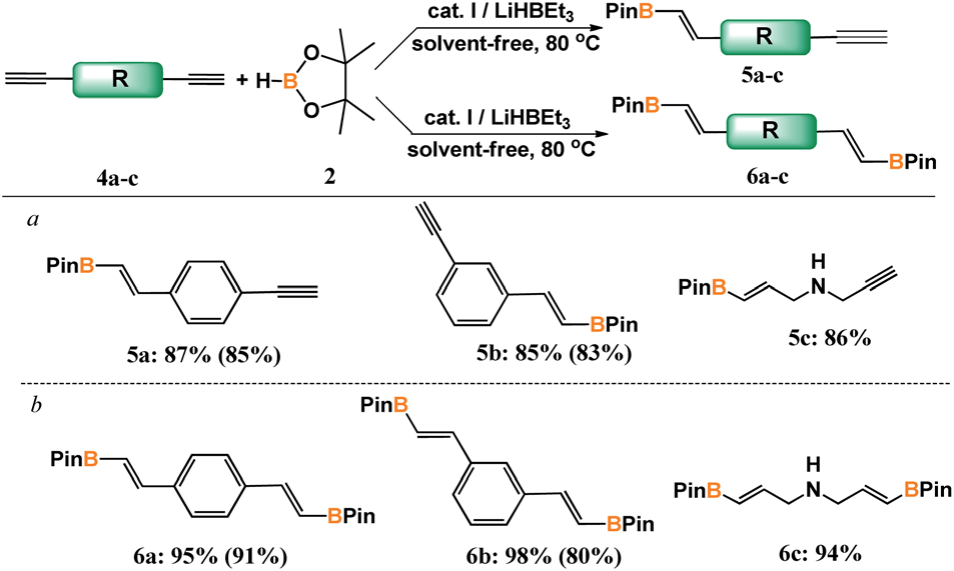
Hydroboration of dialkynes with pinacolborane. ^*a*^ [4a–c] : [2] = 1 : 1.2, [I] = 0.5 mol%. ^*b*^ [4a–c] : [2] = 1 : 2.4, [I] = 1 mol%. Isolated yields are given in parentheses.

At the next stage of the study, the design and developed catalytic system was employed in hydroboration of diynes (4a–c) (Scheme 3).

Depending on the ratio of the reagent concentrations, the products of mono- or disubstitution were obtained. A twofold excess of pinacolborane with respect to diyne led selectively to bis-borylated products in excellent yields. When the reaction was conducted with the reactants at equimolar amounts, we observed the majority of mono-addition product with up to 15% of bis-addition product in the post reaction mixture.

**Scheme 4:**
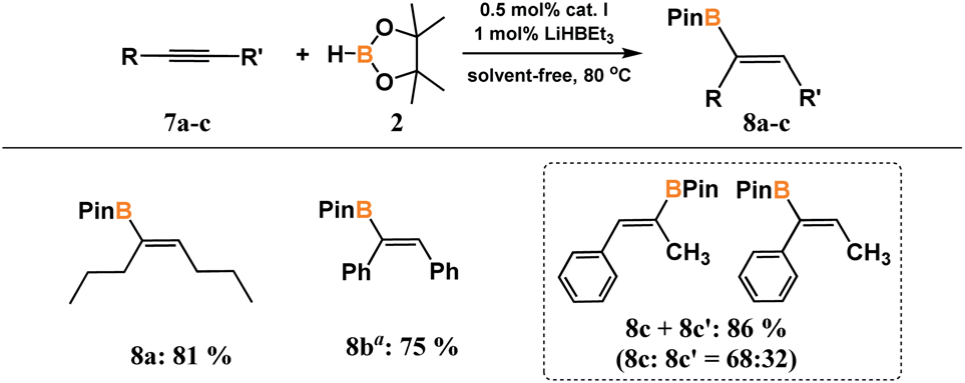
Hydroboration of internal alkynes. ^*a*^0.2 mL of toluene was used because the reactants were in solid state. Isolated yield is given in parentheses.

To investigate the versality of the method, we also decided to test much more challenging symmetrically and unsymmetrically substituted internal acetylenes. For this purpose, unsaturated derivatives having alkyl (7a), aryl (7b) or mixed (7c) substituents at carbon–carbon triple bonds were subjected to reactions with 2 (Scheme 4).

In each case, complete or nearly complete conversion of the reacting partners was achieved under the optimized conditions for the hydroboration of terminal acetylenes. In the reaction with symmetric 4-octyne and diphenylacetylene, only (*Z*)-isomer was obtained exclusively. The use of unsymmetric 1-phenylpropyne led to a mixture of two isomers. The ^1^H NMR analysis confirmed the formation of the products of *syn*-addition 8c and 8cʹ in the ratio of 68 to 32.

In order to determine whether the reaction occurs in a homogenous manner, a mercury poisoning test was performed. The reaction of 4-ethynyltoulene and pinacolborane was performed in standard conditions, then after 30 minutes, 1000 equivalents of mercury in relation to the catalyst were added to the reaction mixture. After 24 hours, 91% conversion of alkyne was detected, which is comparable with the results obtained in the standard test (Table 2, entry 2). Selectivity of the reaction remained the same.

**Scheme 5:**
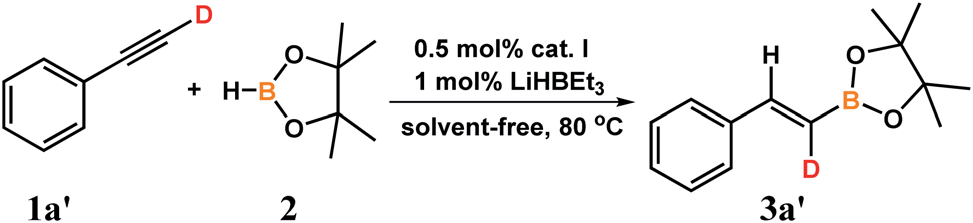
Deuterium-labeling experiment.

We performed also deuterium-labeling experiment using D 1-phenylacetylene (1aʹ) and pinacolborane (2) (Scheme 5):

The experiment was conducted in standard conditions leading to full conversion of reactants after 6 hours. GC-MS analysis exhibited selective formation of the product with the appropriate mass. NMR spectra confirmed formation of syn-addition product with deuterium preserved in the terminal carbon atom. Deuterium-incorporation in the final product was more than 99%.

## 3 Conclusions

In summary, new cobalt complex bearing bulky NHC carbene ligand was synthesized and proven to be catalytically active in the hydroboration of terminal and internal alkynes, leading to the anti-Markovnikov products. Efficient and selective hydroboration of diacetylene with pinacolborane affording mono- and bisborylated products with an exclusive (*E*)-stereochemistry around the newly formed carbon–carbon double bonds was also proposed. The most important advantages of the developed methodologies are full elimination of solvents in the most cases, the use of the catalyst based on the first–row transition metal and its low concentration, full chemoselectivity facilitating isolation of the final product, thereby permitting elimination of additional operations and reduction of production cost and time. Additionally, the effectiveness of the reaction studied was demonstrated to depend on the type of NHC carbene present in the catalyst. Complexes with bulky NHC carbene ligands, in contrast to those with less sterically developed ligands, ensure highly efficient and fully selective course of the process towards formation of the expected product only.

## 4 Experimental

### 4.1. General methods and chemicals

All syntheses and catalytic tests were carried out under dry argon, using standard Schlenk line and vacuum techniques. ^1^H NMR and ^13^C NMR spectra were recorded in CDCl_3_ on a Varian 400 operating at 402.6 and 101.2 MHz, respectively. HSQC spectra were recorded on Bruker Avance DRX 600, operating at frequency of 600.13 MHz (1H). GC analyses were carried out on an Agilent 7890B instrument. GC-MS analyses were performed on a Varian Saturn 2100 T equipped with DB-5, 30 m capillary column and ion trap detector.

MALDI-TOF mass spectra were recorded on a UltrafleXtreme mass spectrometer (Bruker Daltonics, Bremen, Germany), equipped with a SmartBeam II laser (355 nm) in the 500–4000 *m*/*z* range. 2,5-Dihydroxybenzoic acid (DHB, Bruker Daltonics, Bremen, Germany) served as the matrix and was prepared in TA30 solvent (30 : 70 v/v acetonitrile: 0.1% TFA in water) at a concentration of 20 mg mL^−1^. The studied samples were dissolved in dichloromethane (2 mg mL^−1^) and then mixed in a ratio of 1 : 1 v/v with a matrix solution. The matrix/sample mixtures (1 μL) were spotted onto the MALDI target and dried in air. The mass spectra were measured in the reflection mode. The data were analyzed using the software provided with the Ultraflex instrument-FlexAnalysis (version 3.4). The mass calibration (cubic calibration based on five to seven points) was performed using external standards (Peptide Calibration Standard).

Reagents were purchased from commercial sources and were used without further purification. Phenylacetylene-*d* (99 atom% D) was purchased from Sigma-Aldrich. 1,3-bis{2,6-bis(diphenylmethyl)-4-ethylphenyl} imidazolium chloride,^48^ 1,3-bis(2,6-diisopropylphenyl)imidazolium chloride,^49^ 1,3-bis(2,4,6-trimethylphenyl)imidazolium chloride^49^ and 1-mesityl-3-methyl-4-phenyl-1,2,3-triazolium iodide^50,51^ were prepared on the basis of the synthetic methods previously reported. Solvents were dried prior to use over CaH_2_ and stored under argon. THF was purified by distillation over sodium and benzophenone, under argon atmosphere.

### 4.2. Synthesis of NHC-cobalt complexes I

A 25 mL high-pressure Schlenk vessel equipped with a magnetic stirring bar and connected to a gas and vacuum line was charged with imidazolium salt (250 mg, 2.56 × 10^−4^ mol, 1 eq.), potassium bis(trimethylsilyl)amide (56.1 mg, 2.81 × 10^−4^ mol, 1.1 eq.) and dry toluene (5 mL). The reaction mixture was stirred at room temperature for 1 h. After this time, the reaction mixture was filtered off and the volatiles were evaporated. Free carbene was dissolved in dry THF and then cobalt(ii) chloride (33 mg, 2.56 × 10^−4^ mol, 1 eq.) and pyridine (21 μl, 2.56 × 10^−4^ mol, 1 eq.) were added subsequently. The reaction mixture was stirred for 12 h at room temperature. The solvent was removed under reduced pressure and the residual solid was dissolved in a small amount of toluene and filtered through Celite. Hexane was added to the solution. Blue precipitate was filtered off and the product was dried under vacuum.

### 4.3. General procedure for catalytic tests using CoCl_2_/NHC system

A 10 mL high-pressure Schlenk vessel connected to a gas and vacuum line was charged under argon with NHC precursor (3.54 × 10^−6^ mol), cobalt chloride (3.54 × 10^−6^ mol), base (7.08 × 10 ^−6^ mol) and dry THF (1 mL). The suspension was stirred for 30 minutes at room temperature allowing the complex generation. After this time, acetylene (1.77 × 10^−4^ mol), pinacolborane (2.12 × 10^−4^ mol) and 15 μL of decane or dodecane were added subsequently. The reaction mixture was warmed up in an oil bath to 80 °C in a closed vessel. Conversion of the substrate was controlled by GC-MS. Formation of a desire product was confirmed by GC-MS and ^1^H NMR analysis.

### 4.4. General procedure for catalytic tests using complex I

A 10 mL high-pressure Schlenk vessel connected to a gas and vacuum line was charged under argon with acetylene (1.77 × 10 ^−4^ mol), pinacolborane (2.12 × 10^−4^ mol) and 15 μL of decane or dodecane. Cobalt complex I (8.85 × 10^−7^ mol or 1.77 × 10^−6^ mol) and LiHBEt_3_ (1.77 × 10^−6^ mol or 3.54 × 10^−6^ mol) were added subsequently to the reaction mixture. The reaction mixture was warmed up in an oil bath to 80 °C in a closed vessel. Conversion of the substrate was controlled by GC-MS. Formation of a desired product was confirmed by GC-MS and ^1^H NMR analysis.

### 4.5. General procedure for the synthesis of alkynes hydroboration products

A 10 mL high-pressure Schlenk vessel connected to a gas and vacuum line was charged under argon with acetylene (9.03 × 10 ^−4^ mol) and pinacolborane (1.08 × 10^−3^ mol). Cobalt complex I (4.52 × 10^−6^ mol) and LiHBEt_3_ (9.03 × 10^−6^ mol) were added subsequently to the reaction mixture. The reaction mixture was warmed up in an oil bath to 80 °C in a closed vessel for 18 h. Then the solvent was evaporated under vacuum and the resulting product was isolated and purified by chromatography (silica gel 60/n-hexane : DCM = 9 : 1). Evaporation of the solvent gave the analytically pure product.

### 4.6. General procedure for the synthesis of products of diynes mono- and bishydroboration

A 10 mL high-pressure Schlenk vessel connected to a gas and vacuum line was charged under argon with diyne (9.03 × 10 ^−4^ mol) and pinacolborane (2.17 × 10^−3^ mol or4.34 × 10 ^−3^ mol). Cobalt complex I (4.52 × 10^−6^ mol or 9.04 × 10^−6^ mol) and LiHBEt_3_ (9.03 × 10^−6^ mol or 1.8 × 10^−6^ mol) were added subsequently to the reaction mixture. The reaction mixture was warmed up in an oil bath to 80 °C in a closed vessel for 18 h. Then the solvent was evaporated under vacuum and the resulting product was isolated and purified by chromatography (silica gel 60/n-hexane : DCM = 1 : 5). Evaporation of the solvent gave the analytically pure product.

### 4.7. Deuterium-labeling experiment

A 10 mL high-pressure Schlenk vessel connected to a gas and vacuum line was charged under argon with phenylacetylene-d (9.03 × 10^−4^ mol) and pinacolborane (1.08 × 10^−3^ mol). Cobalt complex I (4.52 × 10^−6^ mol) and LiHBEt_3_ (9.03 × 10^−6^ mol) were added subsequently to the reaction mixture. The reaction mixture was warmed up in an oil bath to 80 °C in a closed vessel for 6 h. Then the solvent was evaporated under vacuum and the resulting product was isolated and purified by chromatography (silica gel 60/n-hexane : DCM = 9 : 1). Evaporation of the solvent gave the analytically pure product.

### 4.8. Mercury poisoning test

A 10 mL high-pressure Schlenk vessel connected to a gas and vacuum line was charged under argon with 4-ethyltoluene (1.77 × 10^−4^ mol), pinacolborane (2.12 × 10^−4^ mol) and 15 μL of decane. Cobalt complex I (8.85 × 10^−7^ mol) and LiHBEt_3_ (1.77 × 10 ^−6^ mol) were added subsequently to the reaction mixture. The reaction mixture was warmed up in an oil bath to 80 °C in a closed vessel for 1 hour. After this time the conversion of substrates was measured using gas chromatography. Then 0.15 g of mercury (1000 eq. in relation to catalyst) was added and the reaction was further carried out at 80 °C upon vigorous stirring. Reaction course was controlled by GC-MS. After 24 h the post-reaction mixture was filtrated. Solvent was evaporated and residue was purified by chromatography (silica gel 60/n-hexane : DCM = 9 : 1). Formation of a desired product was confirmed by GC-MS and ^1^H NMR analysis.

## Conflicts of interest

There are no conflicts to declare.

## Supplementary Material

RA-012-D2RA03005E-s001
