# Supplementary material for: Solvent-free hydroboration of alkynes catalyzed by an NHC–cobalt complex
Source: RSC Adv. 2022 Jun 27;12(29):18572–7. doi: 10.1039/d2ra03005e (PMC9234744; doi:10.1039/d2ra03005e)
Supplement: RA-012-D2RA03005E-s001 [file RA-012-D2RA03005E-s001.pdf]

Electronic Supplementary Material (ESI) for RSC Advances.
This journal is © The Royal Society of Chemistry 2022

Supporting information

for

Solvent-free hydroboration of alkynes catalyzed by NHC-cobalt complex

Małgorzata Bołt, Patrycja Żak*

Adam Mickiewicz University in Poznań, Faculty of Chemistry, Uniwersytetu Poznańskiego 8, 61-614 Poznań, Poland

CONTENTS:

1. Analytical data of NHC-cobalt complex **I** S2
2. Analytical data of hydroboration products S2
3. NMR spectra of NHC-cobalt complex **I** S8
4. NMR spectra of isolated products S10
5. References S33

1. Analytical data of NHC-cobalt complex I

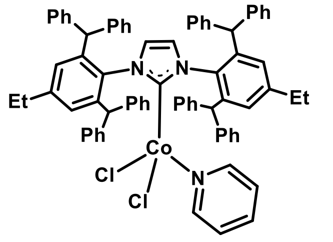

(IPr*^{Et})Co(py)Cl$_2$

^{1}H NMR (600 MHz, $CDCl_3$): δ (ppm) 38.15 (br s, 2H, CH^{Im}), 7.35 (br s, 8H, CH_{Ar}), 7.29 (br s, 5H, CH_{Ar}), 7.24 (br s, 9H, CH_{Ar}), 6.56 (br s, 5H, CH_{Ar}), 6.46 (br s, 3H, CH_{Ar}), 6.25 (br s, 7H, CH_{Ar}), 6.11 (br s, 8H, CH_{Ar}), 5.00 (br s, 8H, CHPh$_2$ and $CH_{Ar\text{-}m}$), 1.99 (br s, 4H, CH_2), 0.59 (br s, 6H, CH_3); ^{13}C NMR (75 MHz, $CDCl_3$): δ (ppm) 146.91, 142.94, 142.24, 141.74, 132.42, 130.90, 130.61, 129.00, 128.90, 128.05, 127.37, 127.34, 126.90, 126.39, 55.26 (CHPh$_2$) , 28.75 (CH$_2$), 15.36 (CH$_3$); MALDI-TOF MS (m/z $[M+Na]^+$): found: 1172.61; calculated for $C_{76}H_{66}Cl_2CoN_3Na$: 1172.38.

2. Analytical data of isolated products

3a: E-2-(4-Methylphenyl)vinylboronic acid pinacol ester[1]

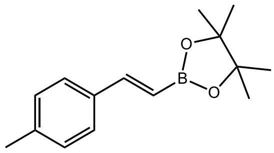

^{1}H NMR (400 MHz, $CDCl_3$): δ (ppm) 7.35-7.40 (m, 3H, CH_{Ar}, CH=), 7.14 (d, J_{HH} = 8.0 Hz, 2H, CH_{Ar}), 6.11 (d, J_{HH} = 18.5 Hz, 1H, CH=) 2.34 (s, 3H, CH_3), 1.31 (s, 12H, CH_3), ^{13}C NMR (75 MHz, $CDCl_3$): δ (ppm) 149.46, 138.95, 134.79, 129.28, 127.01, 83.26, 24.80, 21.32, 1.01; MS: *m/z* (rel. Intensity): 116.00 (13), 117.00 (13), 143.20 (36), 143.90 (21), 158.00 (32), 159 (18), 201.10 (13), 229.20 (13), 243 (30), 244.00 (100, M$^+$).

3b: E-2-(3-Methylphenyl)vinylboronic acid pinacol ester[1]

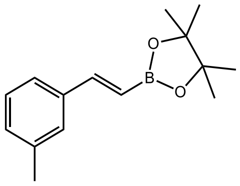

^{1}H NMR (400 MHz, $CDCl_3$): δ (ppm) 7.38 (d, J_{HH} = 18.4 Hz, 1H, CH=), 7.29 – 7.31 (m, 2H, CH_{Ar}), 7.23 (t, J_{HH} = 8.0 Hz, 1H, CH_{Ar}), 7.11 (d, J_{HH} = 8.4 Hz, 1H), 6.15 (d, J_{HH} = 18.4 Hz, 1H, CH=), 2.35 (s, 3H, CH_3), 1.32 (s, 12H, CH_3); ^{13}C NMR (75 MHz, $CDCl_3$): δ (ppm) 149.65, 138.05, 137.40, 129.69, 128.42, 127.75, 124.21, 83.28, 24.78, 21.37; MS: *m/z* (rel. Intensity): 116.10 (25), 117.00 (27), 142.20 (63), 143.20 (34), 158.00 (48), 159.00 (31), 171.10 (10), 201.10 (41), 202.00 (10), 228.20 (10), 229.20 (27), 234.20 (10), 243.20 (34), 244.00 (100, M$^+$).

3c: E-2-(Phenyl)vinylboronic acid pinacol ester[1]

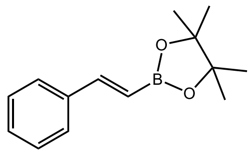

^{1}H NMR (400 MHz, $CDCl_3$): δ (ppm) 7.51-7.48 (m, 2H, CH_{Ar}), 7.41 (d, J_{HH} = 18.5 Hz, 1H, C*H*=), 7.36 – 7.27 (m, 3H, CH_{Ar}), 6.18 (d, J_{HH} = 18.4 Hz, 1H, C*H*=), 1.32 (s, 12H, CH_3); ^{13}C NMR (75 MHz, $CDCl_3$): δ (ppm) 149.49, 137.44, 128.86, 128.53, 127.03, 83.32, 24.78; MS: *m/z* (rel. Intensity): 51.10 (10), 77.20 (11), 105.00 (19), 116.80 (7), 129.20 (56), 130.10 (50), 144.10 (100), 145.00 (48), 173.00 (11), 186.20 (12), 187.20 (38), 2062.0 (11), 215.20 (42), 230.00 (88, M^+).

3d: E-2-(4-Pentylphenyl)vinylboronic acid pinacol ester[4]

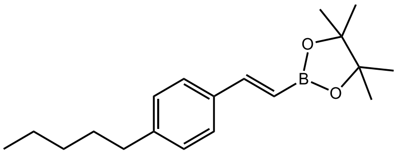

^{1}H NMR (400 MHz, $CDCl_3$): δ (ppm) 7.41 (d, J_{HH} = 8.0 Hz, 2H, CH_{Ar}) + 7.36 (d, J_{HH} = 18.5 Hz, 1H, C*H*=), 7.15 (d, J_{HH} = 8.1 Hz, 2H, CH_{Ar}), 6.12 (d, J_{HH} = 18.5 Hz, 1H, C*H*=), 2.59 (t, J_{HH} = 8.0 Hz, 2H, CH_2), 1.66 – 1.56 (m, 2H, CH_2), 1.31 (s, 16H, CH_3 + CH_2), 0.89 (t, J_{HH} = 6.9 Hz, 3H, CH_3); ^{13}C NMR (75 MHz, $CDCl_3$): δ (ppm) 149.51, 144.03, 134.97, 128.61, 127.00, 83.24, 35.73, 31.46, 30.97, 24.79, 22.51, 13.99; MS: *m/z* (rel. Intensity): 115.00 (7), 117.00 (8), 129.20 (7), 143.20 (51), 144.10 (18), 157.10 (10), 184.10 (11), 199.00 (11), 214.20 (32), 215.10 (18), 244.00 (17), 257.10 (5), 285.10 (24), 299.10 (30), 299.80 (100, M^+).

3e: E-2-(4-tert-Butylphenyl)vinylboronic acid pinacol ester[2]

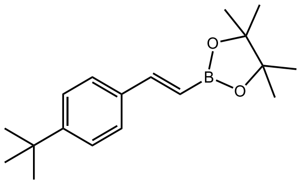

^{1}H NMR (400 MHz, $CDCl_3$): δ (ppm) 7.44 – 7.35 (m, 5H, C*H*(Ar) + C*H*=), 6.13 (d, J_{HH} = 18.5 Hz, 1H, C*H*=), 1.32 (s, 21H, CH_3(BPin) + C(CH_3)); ^{13}C NMR (75 MHz, $CDCl_3$): δ (ppm) 152.10, 149.37, 134.75, 126.81, 125.48, 83.25, 34.67, 31.22, 24.98; MS: *m/z* (rel. Intensity): 55.20 (2), 57.10 (7), 128.20 (2), 142.20 (3), 143.20 (6), 171.10 (4), 185.00 (2), 213.20 (1), 271.20 (100, $M^+ - CH_3$), 272.10 (18), 285.80 (14), 286.80 (3, M^+).

***3f: E*-2-(4-Methoxyphenyl)vinylboronic acid pinacol ester[1]**

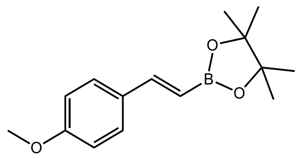

^{1}H NMR (400 MHz, $CDCl_3$): δ (ppm) 7.44 (d, J_{HH} = 8.8 Hz, 2H, CH_{Ar}), 7.35 (d, J_{HH} = 18.4 Hz, 1H, C*H*=), 6.87 (d, J_{HH} = 8.8 Hz, 2H, CH_{Ar}), 6.01 (d, J_{HH} = 18.4 Hz, 1H, C*H*=), 3.81 (s, 3H, OCH_3), 1.31 (s, 12H, CH_3); ^{13}C NMR (75 MHz, $CDCl_3$): δ (ppm) 160.28, 149.05, 130.41, 128.45, 113.96, 83.21, 55.27, 24.79; MS: *m/z* (rel. Intensity): 116.80 (4), 143.20 (10), 144.80 (5), 159.00 (16), 159.90 (10), 173.90 (10), 174.80 (9), 217.00 (6), 245.10 (6), 259.80 (27), 260.80 (100, M^+).

***3g: E*-2-(4-(*N,N*-dimethylamino)phenyl)vinylboronic acid pinacol ester[8]**

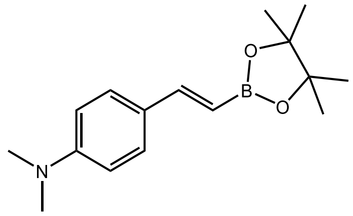

^{1}H NMR (400 MHz, $CDCl_3$): δ (ppm) 7.40 (d, J_{HH} = 8.8 Hz, 2H, CH_{Ar}), 7.33 (d, J_{HH} = 18.4 Hz, 1H, C*H*=), 6.67 (d, J_{HH} = 8.8 Hz, 2H, CH_{Ar}), 5.92 (d, J_{HH} = 18.4 Hz, 1H, C*H*=), 2.98 (s, 6H, N(CH_3)$_2$), 1.3 (s, 12H, CH_3); MS: *m/z* (rel. Intensity): 272.40 (13), 273.30 (100, M^+).

***3j: E*-2-(Heptyl)vinylboronic acid pinacol ester[3]**

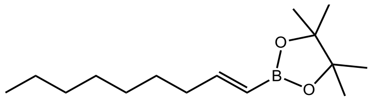

^{1}H NMR (400 MHz, $CDCl_3$): δ (ppm) 6.63 (dt, J_{HH} = 18.0, 6.5 Hz, 1H, C*H*=), 5.42 (dt, *J* = 18.0, 1.6 Hz, 1H C*H*=), 2.10 – 2.17 (m, 2H, CH_2), 1.36 -1.44 (m, 2H, CH_2), 1.26 (s, 20H, CH_3 + CH_2 overlapping), 0.87 (t, J_{HH} = 7.1 Hz, 3H, CH_3) ^{13}C NMR (75 MHz, $CDCl_3$): δ (ppm) 154.87, 82.95, 35.82, 31.79, 29.15, 28.20, 24.75, 22.65, 14.09; MS: *m/z* (rel. Intensity): 55.20 (20), 57.10 (13), 67.20 (22), 69.00 (13), 81.10 (33), 83.00 (25), 95.10 (21), 109.10 (27), 111.00 (41), 123.20 (18), 139.20 (10), 152.20 (36), 153.20 (100), 166.20 (16), 167.10 (22), 194.10 (10), 195.00 (23), 236.20(20), 237.20 (73), 252.80 (25, M^+).

***3l: E*-2-(naphthalen-1-yl)vinylboronic acid pinacol ester[7]**

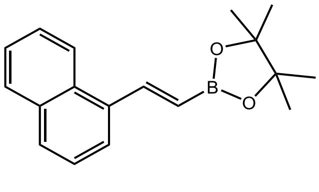

^{1}H NMR (400 MHz, $CDCl_3$): δ (ppm) 8.27 (d, J_{HH} = 8.2 Hz, 1H, CH_{Ar}), 8.21 (d, J = 18.1, Hz, 1H CH=), 7.86 – 7.80 (m, 2H, CH_{Ar}) 7.73 (d, J_{HH} = 7.2 Hz, 1H, CH_{Ar}), 7.55 – 7.45 (m, 3H, CH_{Ar}), 6.26 (d, J_{HH} = 17.9 Hz, 1H, CH_{Ar}), 1.35 (s, 12H, CH_3); ^{13}C NMR (75 MHz, $CDCl_3$): δ (ppm) 146.43, 135.32, 133.58, 131.07, 129.01, 128.47, 126.15, 125.78, 125.58, 124.06, 123.77, 83.41, 24.85; MS: *m/z* (rel. Intensity): 69.20 (15), 83.30 (8), 84.20 (16), 152.20 (19), 153.00 (19), 179.20 (22), 180.00 (13), 223.20 (7), 237.20 (6), 265.30 (6), 279.20 (29), 279.90 (100, M^+).

3m: *E*-2-(Phenanthren-9-yl)vinylboronic acid pinacol ester[7]

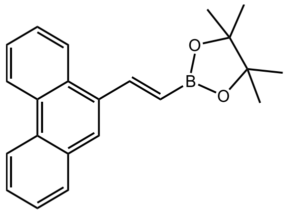

^{1}H NMR (400 MHz, $CDCl_3$): δ (ppm) 8.73 (d, J_{HH} = 8.1, Hz, 1H, CH_{Ar}), 8.66 (d, J_{HH} = 8.1, Hz, 1H, CH_{Ar}), 8.29 (d, J_{HH} = 7.6, Hz, 1H, CH_{Ar}), 8.21 (d, J_{HH} = 18.0, Hz, 1H CH=), 7.96 (s, 1H, CH_{Ar}) 7.89 (d, J_{HH} = 7.7, Hz, 1H, CH_{Ar}), 7.70 – 7.59 (m, 4H, CH_{Ar}), 6.35 (d, J_{HH} = 18.0 Hz, 1H, CH_{Ar}), 1.37 (s, 12H, CH_3); ^{13}C NMR (75 MHz, $CDCl_3$): δ (ppm) 147.18, 134.60, 131.62, 130.60, 130.32, 130.28, 129.03, 126.87, 126.75, 126.64, 126.51, 125.34, 124.67, 123.01, 122.50, 83.46, 24.88; MS: m/z (rel. Intensity): 69.20 (13), 84.20 (19), 202.20 (36), 203.00 (24), 214.30 (9), 229.30 (26), 231.20 (11), 273.20 (10), 330.00 (100, M^+).

3n: (*E*)-2-(4-(trifluoromethyl)phenyl)vinylboronic acid pinacol ester[6]

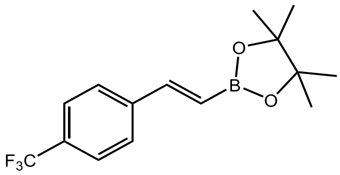

^{1}H NMR (400 MHz, $CDCl_3$): δ (ppm) 7.61 – 7.56 (m, 4H, CH_{Ar}), 7.40 (d, J_{HH} = 18.3 Hz, 1H, CH=), 6.26 (d, J_{HH} = 18.4 Hz, 1H, CH=), 1.32 (s, 12H, CH_3); ^{13}C NMR (75 MHz, $CDCl_3$): δ (ppm) 147.67, 127.13, 125.6 (q, J = 4.0 Hz) 83.60, 24.80; MS: m/z (rel. Intensity): 208.70 (31), 209.80 (36), 210.50 (37), 211.10 (41), 212.20 (57), 213.20 (35), 279.50 (64), 282.40 (32), 283.20 (100), 298.10 (35, M^+).

3o: (*E*)-2-(cyclohexyl)vinylboronic acid pinacol ester[6]

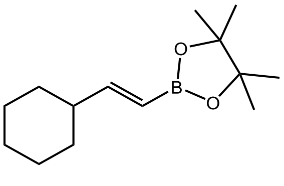

^{1}H NMR (400 MHz, $CDCl_3$): δ (ppm) 6.57 (dd, J_{HH} = 18.1, 6.2 Hz, 1H, CH=), 5.37 (dd, J_{HH} = 18.1, 1.5 Hz, 1H, CH=), 2.06 – 1.98 (m, 1H, CH), 1.77 – 1.69 (m, 4H, CH_2), 1.66 – 1.60 (m, 1H, CH_2),

1.26 (s, 12H, CH_3), 1.25 – 1.02 (m, 5H, CH_2); ^{13}C NMR (75 MHz, $CDCl_3$): δ (ppm) 159.87, 82.96, 43.24, 31.89, 26.14, 25.94, 24.77; MS: m/z (rel. Intensity): 81.20 (40), 83.20 (62), 84.20 (100), 85.10 (40), 107.20 (38), 108.20 (70), 109.10 (37), 135.20 (63), 153.20 (33), 178.20 (41), 179.20 (95), 221.30 (47), 236.20 (42, M^+).

3p: (*E*)-2-(Thiophen-3-yl)vinylboronic acid pinacol ester[1]

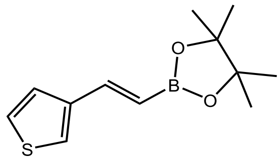

^{1}H NMR (400 MHz, $CDCl_3$): δ (ppm) 7.38 (d, J_{HH} = 18.4 Hz, 1H, CH=), 7.32 – 7.30 (m, 2H, CH_{Ar}), 7.27 – 7.25 (m, 1H, CH_{Ar}), 5.94 (d, J_{HH} = 18.4 Hz, 1H, CH=), 1.30 (s, 12H, CH_3); ^{13}C NMR (75 MHz, $CDCl_3$): δ (ppm) 143.12, 141.21, 126.08, 125.01, 124.82, 83.28, 24.78; MS: m/z (rel. Intensity): 135.00 (45), 148.80 (36), 162.20 (47), 162.80 (48), 190.30 (38), 191.50 (53), 192.50 (80), 193.80 (83), 194.10 (38), 235.70 (54), 236.70 (94, M^+).

3r: (*E*)-2-(4-bromophenyl)vinylboronic acid pinacol ester[6]

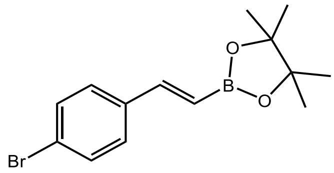

^{1}H NMR (400 MHz, $CDCl_3$): δ (ppm) 7.46 (d, J_{HH} = 8.5 Hz, 1H, CH_{Ar}), 7.34 (d, J_{HH} = 8.4 Hz, 1H, CH_{Ar}), 7.32 (d, J_{HH} = 18.3 Hz, 1H, CH=), 6.15 (d, J_{HH} = 18.2 Hz, 1H, CH=), 1.31 (s, 12H, CH_3); ^{13}C NMR (75 MHz, $CDCl_3$): δ (ppm) 148.04, 136.34, 131.72, 130.66, 129.75, 128.48, 122.88, 83.45, 24.78; MS: *m/z* (rel. Intensity): 142.50 (23), 143.50 (26), 211.70 (24), 214.30 (24), 217.50 (23), 305.80 (24), 306.80 (34), 307.80 (54), 308.70 (100), 309.50 (84, M^+).

3s: *E*-2-(2-Methylphenyl)vinylboronic acid pinacol ester

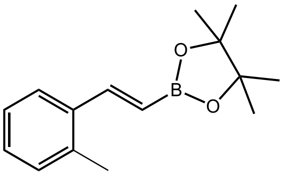

^{1}H NMR (400 MHz, $CDCl_3$): δ (ppm) 7.65 (d, J_{HH} = 18.3 Hz, 1H, CH=), 7.52 – 7.58 (m, 1H, CH_{Ar}), 7.11-7.22 (m, 3H, CH_{Ar}), 6.09 (d, J_{HH} = 18.3 Hz, 1H, CH=), 2.42 (s, 3H, CH_3), 1.32 (s, 12H, CH_3); ^{13}C NMR (75 MHz, $CDCl_3$): δ (ppm) 147.08, 136.65, 136.27, 130.36, 128.54, 126.07, 125.73, 83.27, 24.80, 19.81; MS: *m/z* (rel. Intensity): 85.2 (10), 115.2 (43), 116.1 (41), 117.0 (34), 141.2 (22), 142.2 (30), 143.2 (48), 144.1 (38), 145.0 (16), 201.2 (16), 231.5 (20), 234.2 (25), 241.0 (19), 242.4 (28), 243.3 (51), 244.3 (100, M^+).

3t: *E*-2-(2-fluorophenyl)vinylboronic acid pinacol ester

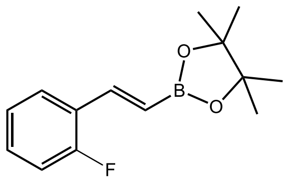

^{1}H NMR (400 MHz, $CDCl_3$): 7.52-7.61 (m, 2H, CH_{Ar} + C*H*=), 7.27-7.25, 7.21 – 7.24 (m, 1H, CH_{Ar}), 7.07 – 7.12 (m, 1H, CH_{Ar}), 6.99-7.04 (m, 1H, CH_{Ar}), 6.22 (d, J_{HH} = 18.6 Hz, 1H, C*H*=), 1.30 (s, 12H, CH_3); ^{13}C NMR (75 MHz, $CDCl_3$): 160.67 (d, *J* = 251.5 Hz), 130.15 (d, *J* = 8.4 Hz), 127.35 (d, *J* = 3.2 Hz), 125.33 (d, *J* = 11.5 Hz), 124.07 (d, *J* = 3.4 Hz), 115.79 (d, *J* = 22.0 Hz), 83.43, 24.79; MS: *m/z* (rel. Intensity): 77.0 (19), 101.9 (30), 103.0 (22), 128.0 (26), 132.0 (25), 135.0 (21), 146.2 (19), 148.0 (80), 149.0 (31), 161.0 (24), 162.0 (100), 163.1 (73), 175.0 (25), 205.1 (36), 232.1 (22), 233.0 (64), 247.0 (20), 247.8 (29)

***5a: (E)*-2-(4-ethynylbenzene)vinylboronic acid pinacol ester[10]**

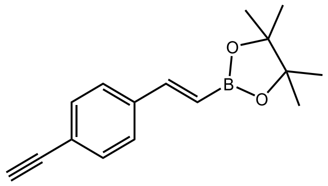

^{1}H NMR (400 MHz, $CDCl_3$): δ (ppm) 7.41 – 7.49 (m, 4H, CH_{Ar}), 7.36 (d, J_{HH} = 18.4 Hz, 1H, C*H*=), 6.16 (d, J_{HH} = 18.4 Hz, 1H, C*H*=), 3.13 (s, 1H, ≡C*H*), 1.31 (s, 12H, CH_3); ^{13}C NMR (75 MHz, $CDCl_3$): δ (ppm) 148.55, 137.96, 132.51, 127.05, 122.53, 83.62, 78.39, 24.96; MS: *m/z* (rel. Intensity): 126. 10 (20), 127.00 (20), 151.30 (21), 152.30 (39), 153.30 (76), 154.10 (64), 155.00 (36), 166.30 (25), 167.20 (44), 168.20 (67), 169.20 (35), 238.50 (27), 239.50 (63), 240.50 (22), 253.00 (40), 254.00 (100, M^+).

***5b: (E)*-2-(3-ethynylbenzene)vinylboronic acid pinacol ester[12]**

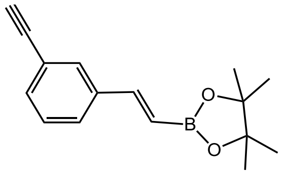

^{1}H NMR (400 MHz, $CDCl_3$): δ (ppm) 7.60 (t, *J* = 1.7 Hz, 1H, CH_{Ar}), 7.47 (d, *J* = 7.8 Hz, 1H, CH_{Ar}), 7.41 (dt, *J* = 7.7, 1.4 Hz, 2H, CH_{Ar}), 7.34 (d, J_{HH} = 18.5 Hz, 1H, C*H*=), 6.17 (d, J_{HH} = 18.4 Hz, 1H, C*H*=), 3.07 (s, 1H, ≡C*H*), 1.31 (s, 12H, CH_3); ^{13}C NMR (75 MHz, $CDCl_3$): δ (ppm) 148.27, 132.34, 130.74, 129.52, 128.58, 128.42, 127.35, 126.54, 122.44, 83.45, 83.36, 24.80; MS: *m/z* (rel. Intensity): 154.10 (31), 168.00 (41), 239.20 (13), 254.20 (100, M^+).

6a: 1,4-Benzene-*E*-divinylboronic acid dipinacol ester[5]

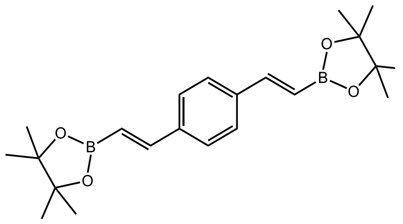

^{1}H NMR (400 MHz, $CDCl_3$): δ (ppm) 7.45 (s, 4H, CH_{Ar}), 7.37 (d, J_{HH} = 18.41 Hz, 2H, C*H*=), 6.17 (d, J_{HH} = 18.25 Hz, 2H, C*H*=), 1.31 (s, 24H, CH_3); ^{13}C NMR (75 MHz, $CDCl_3$): δ (ppm) 148.83, 137.94, 127.30, 116.86, 83.37, 24.78; MS: *m/z* (rel. Intensity): 55.20 (5), 83.50 (4), 139.00 (3), 181.10 (5), 195.30 (10), 266.40 (4), 296.50 (3), 367.90 (5), 382.20 (100, M^+).

6b: 1,3-Benzene-*E*-divinylboronic acid dipinacol ester[11]

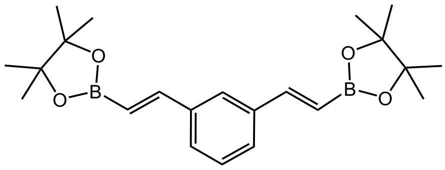

^{1}H NMR (400 MHz, $CDCl_3$): δ (ppm) 7.56 (s, 1H, CH_{Ar}), (7.45 – 7.42 (m, 1H, CH_{Ar}), 7.38 (d, J_{HH} = 18.4 Hz, 2H, C*H*=), 7.33 – 7.29 (m, 2H, CH_{Ar}), 6.17 (d, J_{HH} = 18.4 Hz, 2H, C*H*=), 1.32 (s, 24H, CH_3); ^{13}C NMR (75 MHz, $CDCl_3$): δ (ppm) 149.13, 137.73, 128.80, 127.32, 126.14, 83.38, 24.80; MS: *m/z* (rel. Intensity): 55.30 (10), 83.20 (10), 84.50 (10), 85.10 (10), 180.30 (10), 181.20 (12), 195.50 (16), 195.30 (13), 296.70 (16), 367.90 (15), 381.70 (55), 382.40 (100, M^+).

8c: *(Z)*-4,4,5,5,-tetramethyl-2-(1-phenylprop-1-en-1-yl)-1,3,2-dioxaborolane and *4c': (Z)*-4,4,5,5-tetramethyl-2-(1-phenylprop-1-en)-2-yl)-1,3,2-diocaborolane[1]

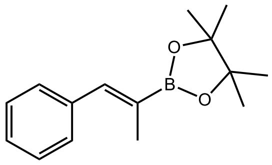

4c

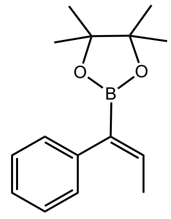

4c'

^{1}H NMR (400 MHz, $CDCl_3$): for 4c: δ (ppm) 7.40–7.37 (m, 2H, CH_{Ar}), 7.37–7.34 (m, 2H, CH_{Ar}), 7.22–7.19 (m, 1H, CH_{Ar}), 7.18–7.15 (m, 1H, C*H*=), 1.99 (d, J_{HH} = 1.8 Hz, 3H, CH_3), 1.32 (s, 12H, CH_3); for 4c': δ (ppm) 7.33–7.30 (m, 2H, CH_{Ar}), 7.26–7.23 (m, 3H, CH_{Ar}), 6.72 (q, J_{HH} = 6.9 Hz, 1H, C*H*=), 1.77 (d, J_{HH} = 6.9 Hz, 3H, CH_3), 1.27 (s, 12H, CH_3); ^{13}C NMR (75 MHz, $CDCl_3$): for 4c + 4c': δ (ppm) 142.77, 142.35, 139.74, 137.89, 129.38, 129.06, 128.01, 127.73, 127.07, 125.85, 83.50, 24.82, 24.71, 15.97, 15.88; MS: m/z (rel. Intensity): for 4c: 105.00 (14), 116.10 (14), 143.20 (61), 187.00 (100), 228.80 (12), 243.80 (10); for 4c': 102.80 (5), 116.10 (10), 143.20 (31), 158.00 (10), 187.20 (8), 229.20 (11), 244.00 (100, M^+).

(E)-1-deuterium-2-(phenyl)vinylboronic acid pinacol ester[2]

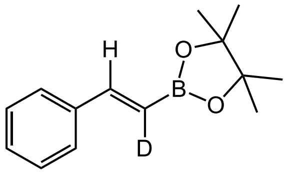

^{1}H NMR (400 MHz, $CDCl_3$): δ (ppm) 7.51-7.48 (m, 2H, CH_{Ar}), 7.40 (s, 1H, C*H*=), 7.36 – 7.26 (m, 3H, CH_{Ar}), lack of signals in the range 6.00 – 6.40; 1.32 (s, 12H, CH_3); ^{13}C NMR (75 MHz, $CDCl_3$): δ (ppm) 149.42, 137.40, 128.86, 128.54, 127.03, 83.31, 24.78; MS: *m/z* (rel. Intensity): 77.90 (13), 104.80 (17), 130.20 (61), 145.0 (100), 158.00 (11), 174.10 (11), 188.20 (29), 216.20 (45), 216.20 (45), 231.00 (80), 231.90 (42, M^+).

3. NMR spectra of NHC-cobalt complex I

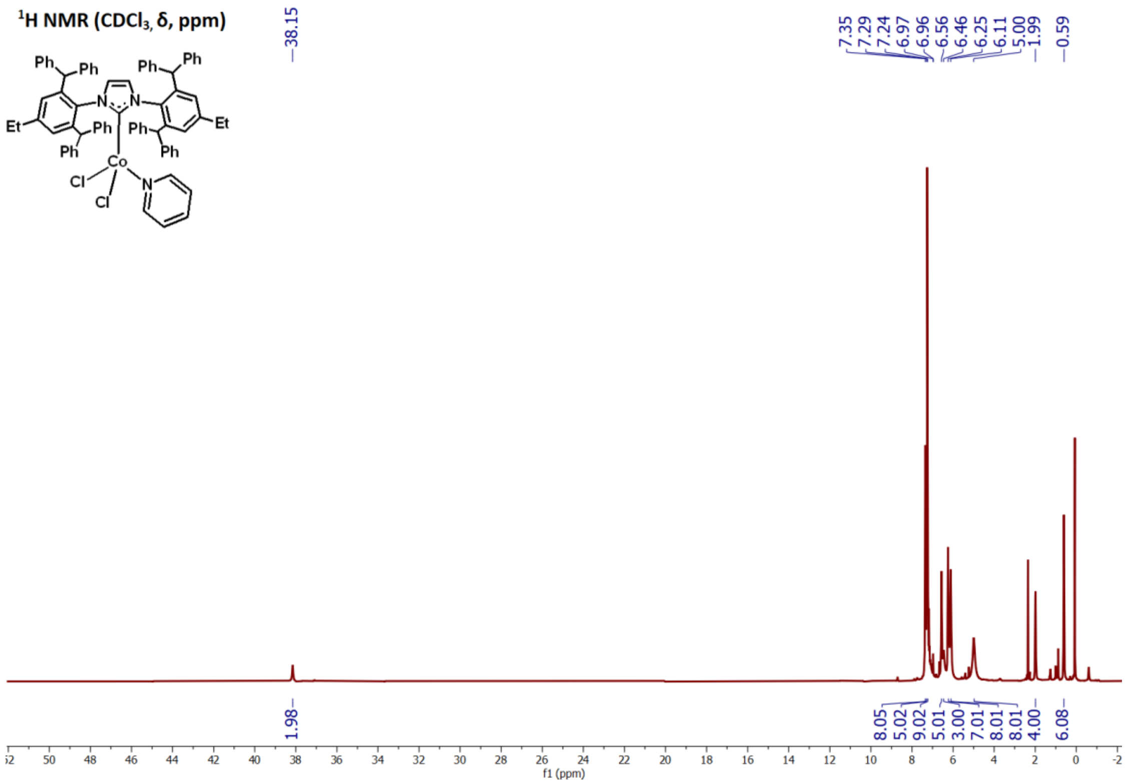

Figure 1. ^{1}H NMR (600 MHz, δ, $CDCl_3$) of complex **I**

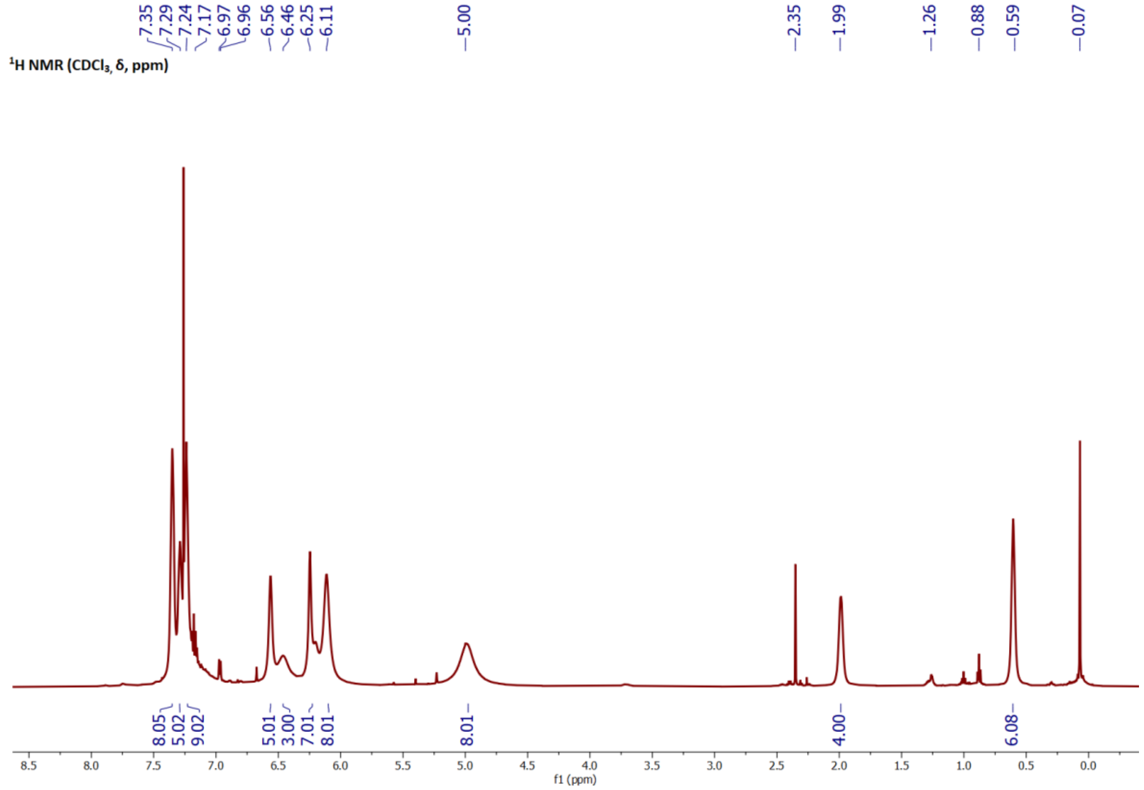

Figure 2. ^{1}H NMR (600 MHz, δ, $CDCl_3$) of complex **I** – expanded aliphatic and aromatic region. Additional signals from impurities: 0.07 ppm – silicon grease, 0.88 ppm and 1.26 pm – hexane, 2.35 and 7.17 ppm – toluene.

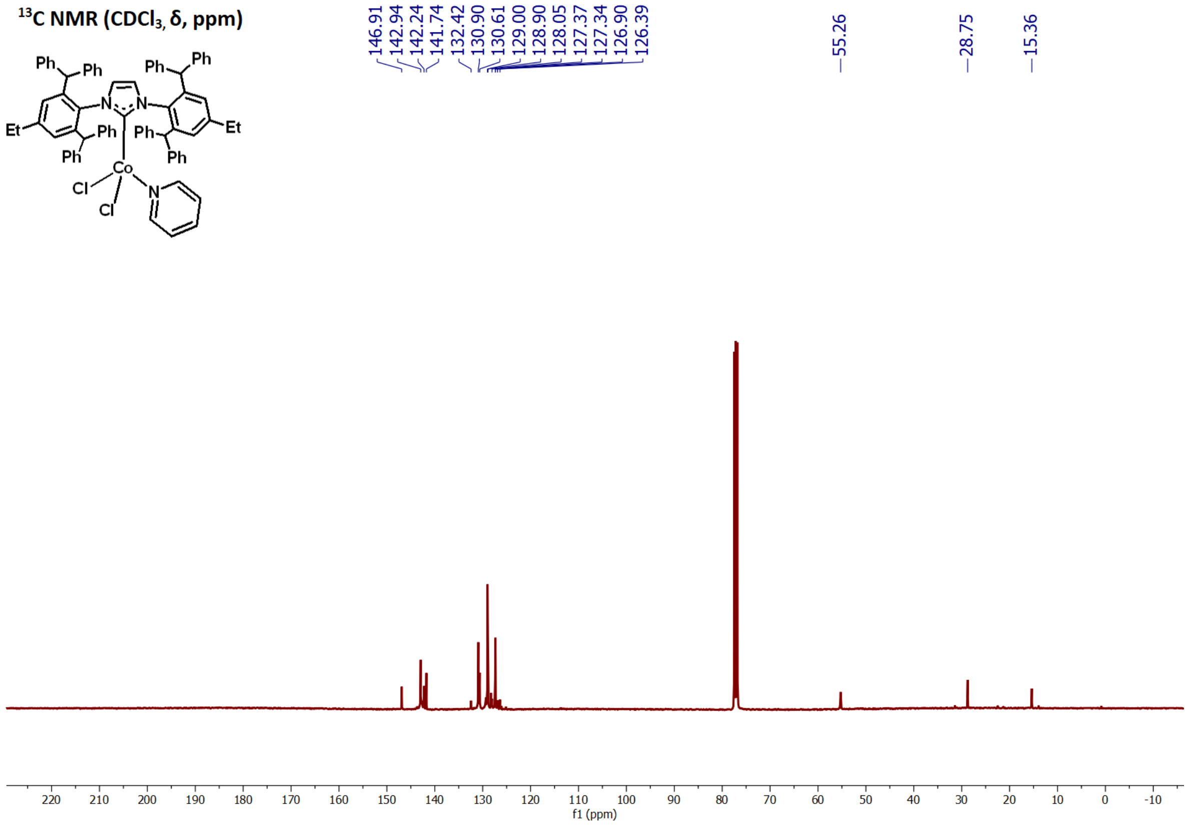

Figure 3. ^{13}C NMR (150 MHz, δ, $CDCl_3$) of complex **I**

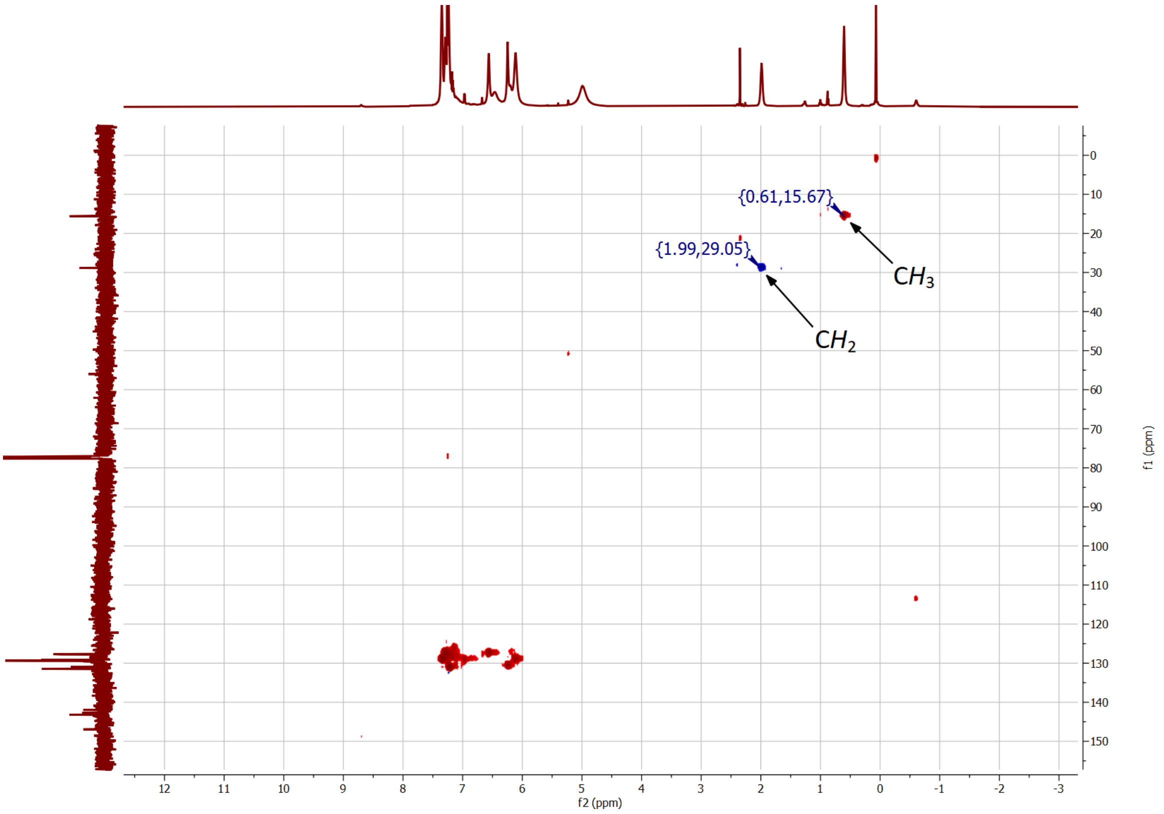

Figure 4. $^{1}H,^{13}C$ -HSQC NMR (600, 150 MHz, δ, $CDCl_3$) of complex **I**

4. NMR spectra of isolated products

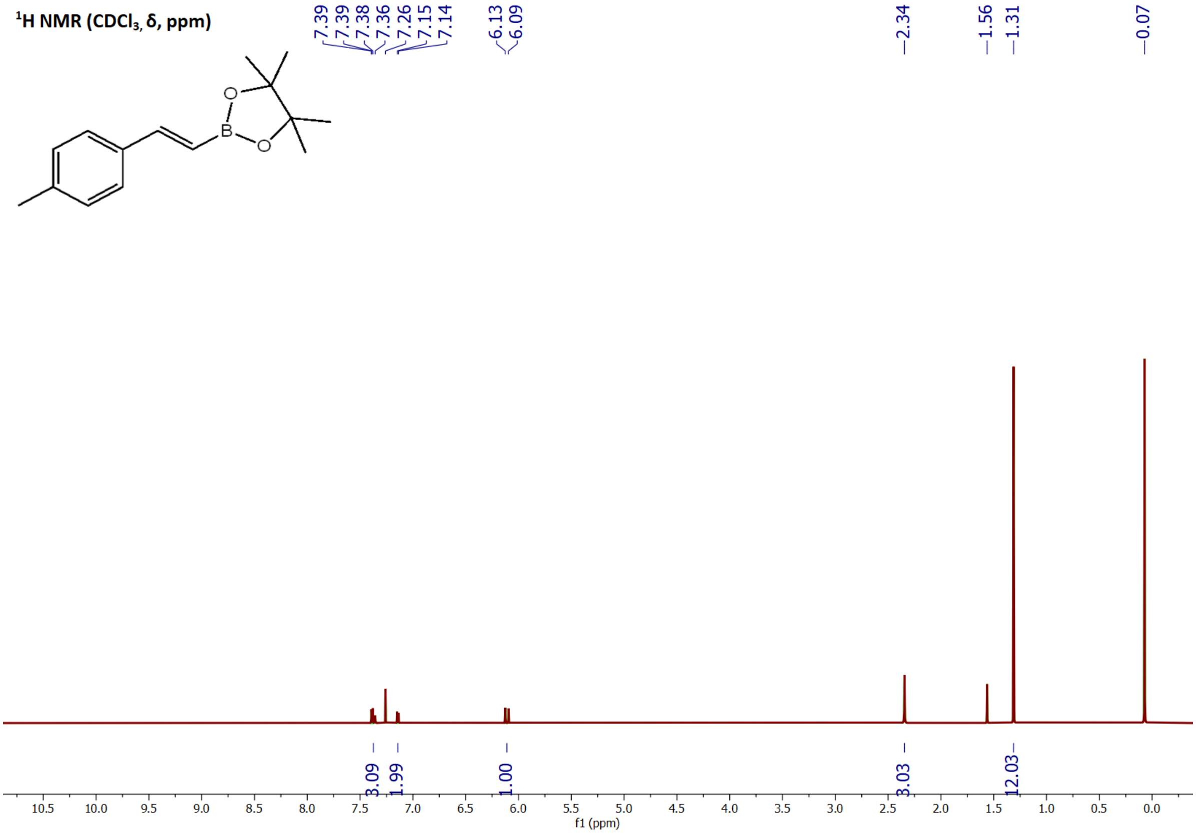

Figure 5. ^{1}H NMR (400 MHz, δ, $CDCl_3$) of **3a**

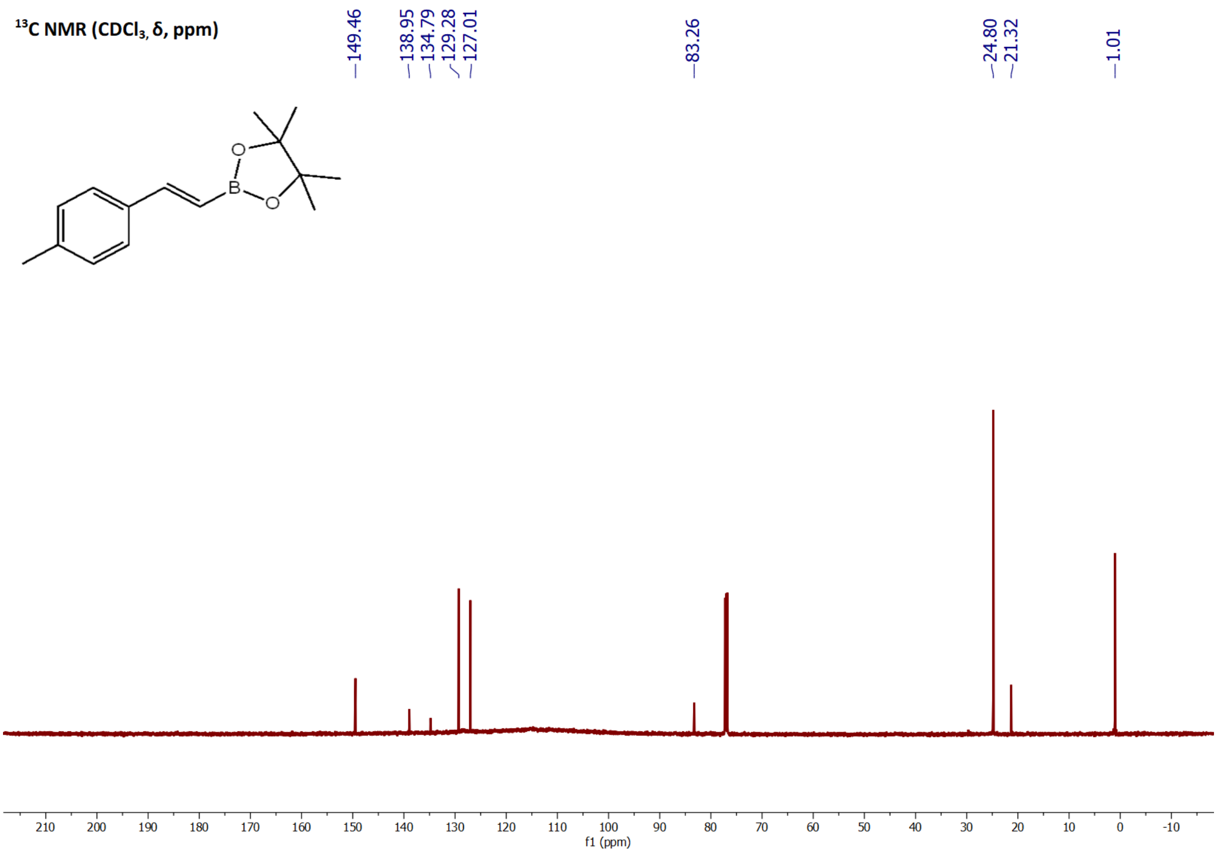

Figure 6. ^{13}C NMR (75 MHz, δ, $CDCl_3$) of **3a**

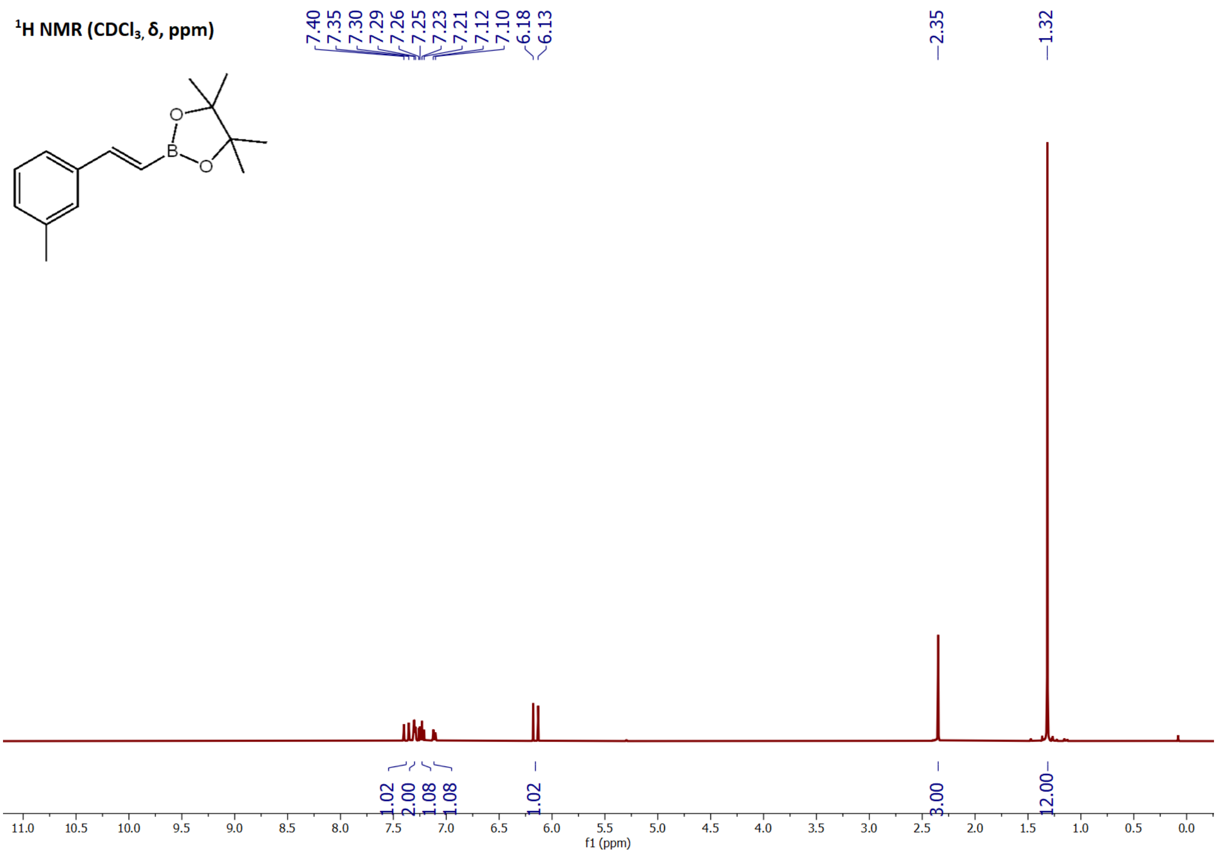

Figure 7. ^{1}H NMR (400 MHz, δ, $CDCl_3$) of **3b**

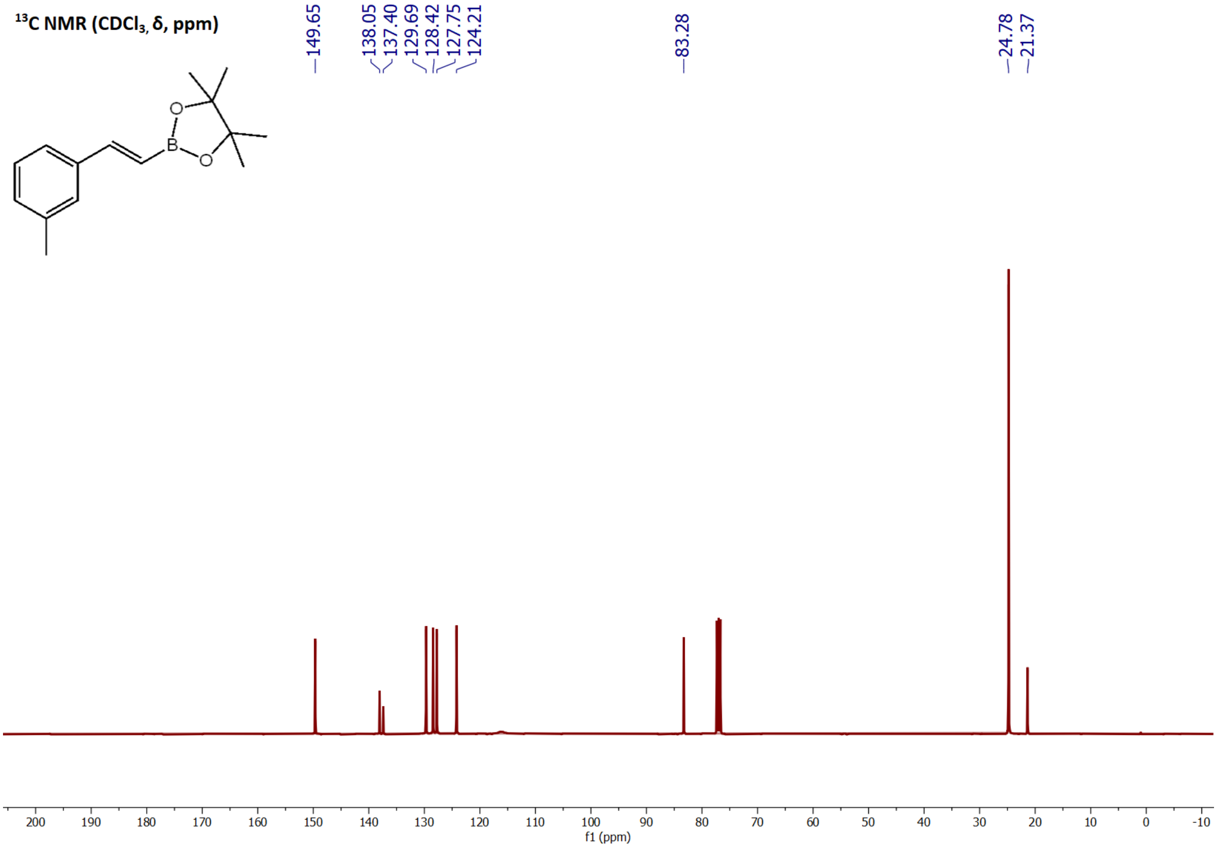

Figure 8. ^{13}C NMR (75 MHz, δ, $CDCl_3$) of **3b**

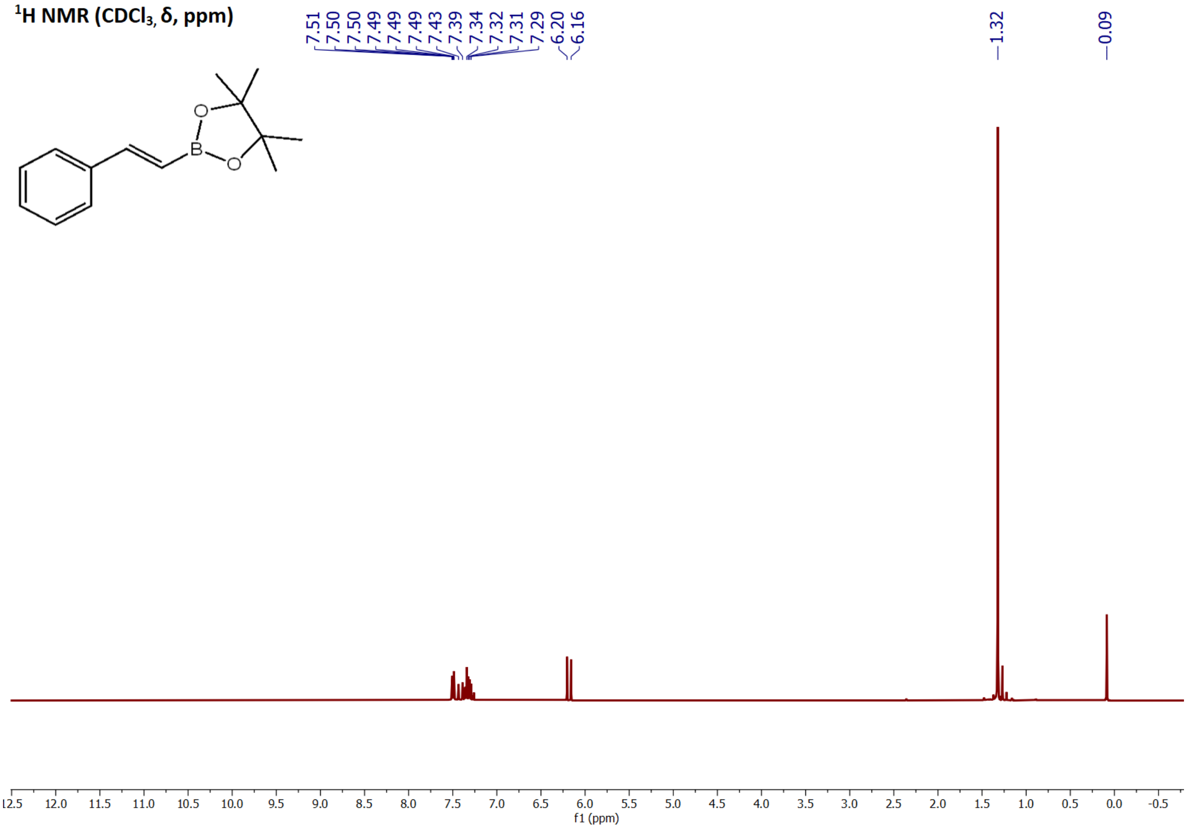

Figure 9. ^{1}H NMR (400 MHz, δ, $CDCl_3$) of **3c**

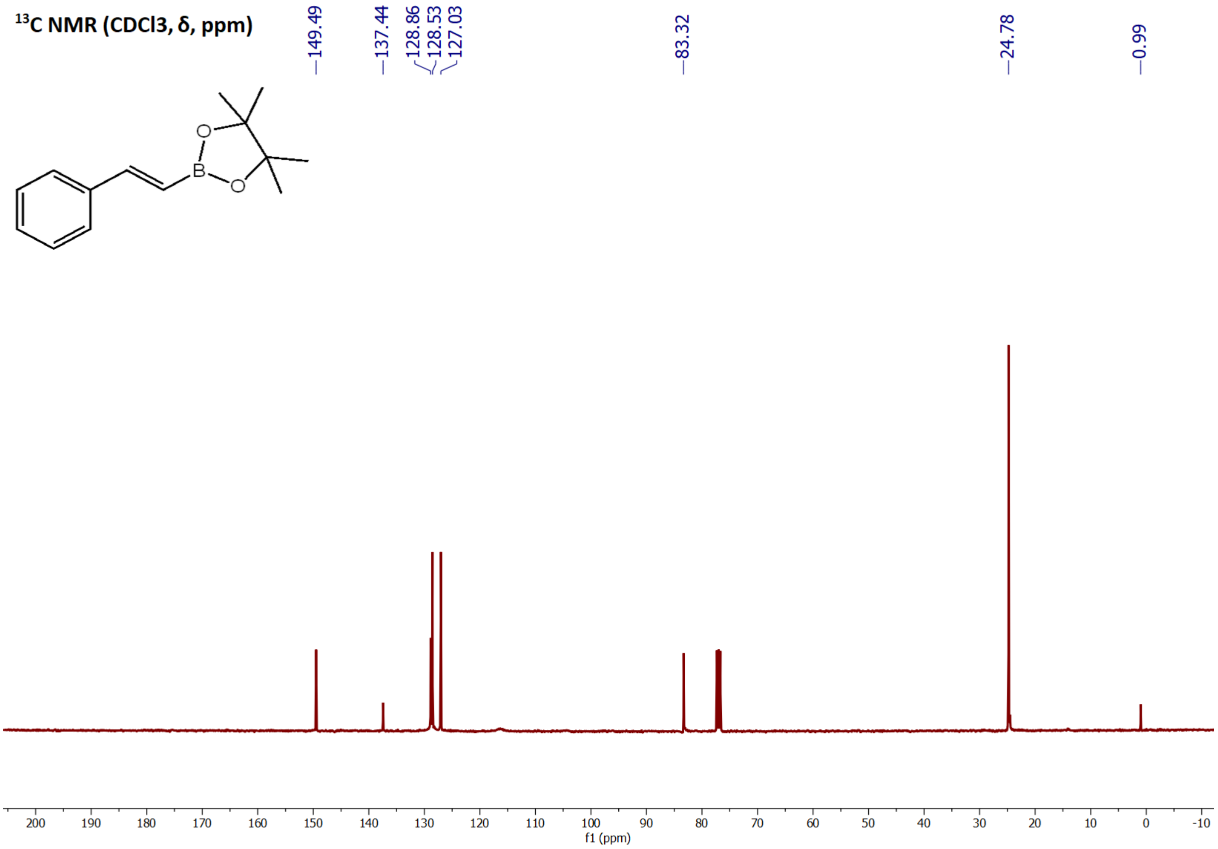

Figure 10. ^{13}C NMR (75 MHz, δ, $CDCl_3$) of **3c**

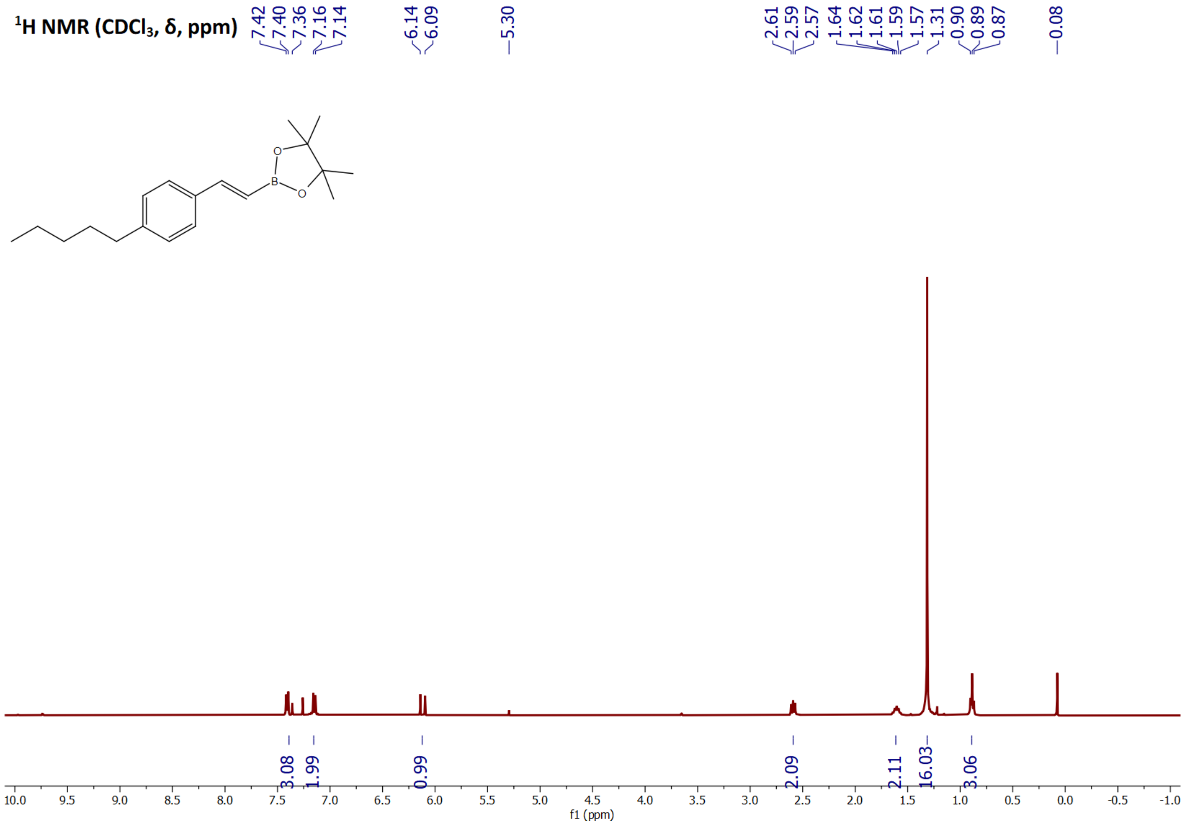

Figure 11. ^{1}H NMR (400 MHz, δ, $CDCl_3$) of **3d**

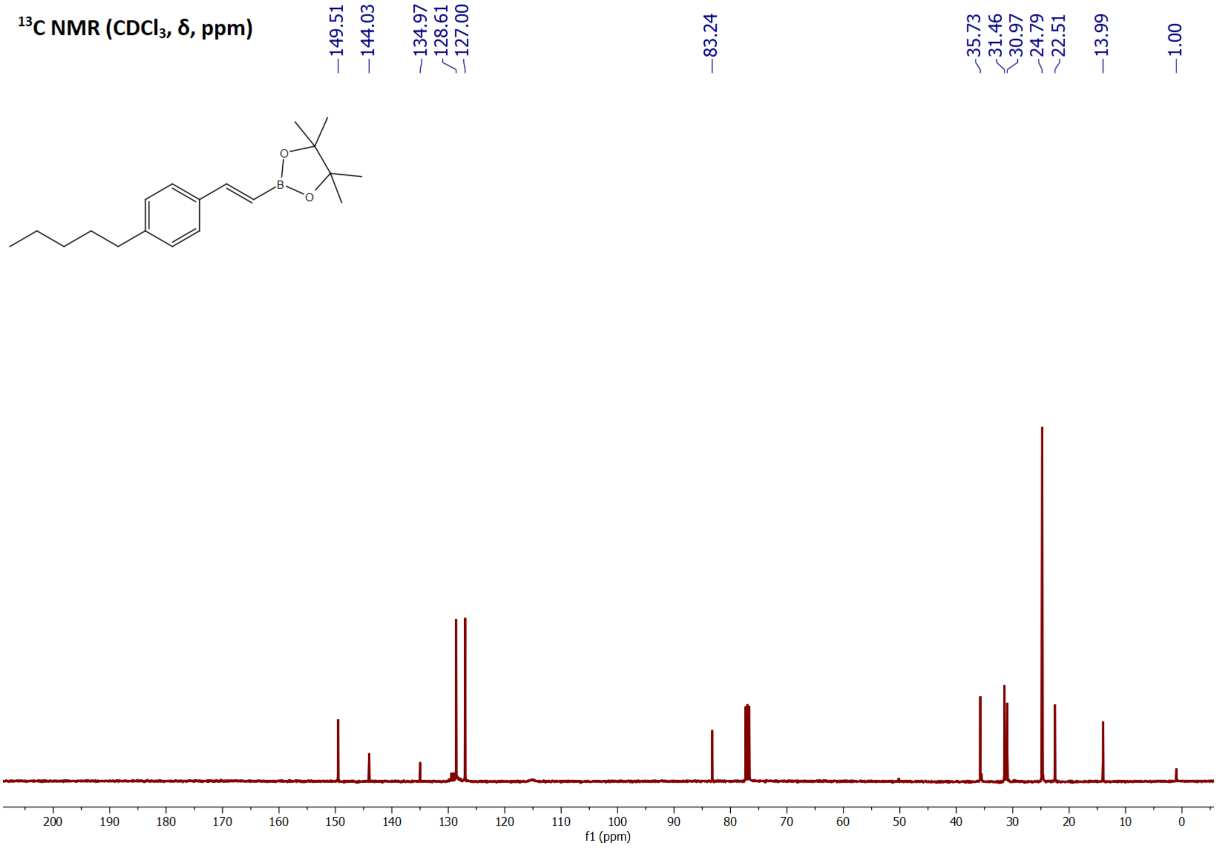

Figure 12. ^{13}C NMR (75 MHz, δ, $CDCl_3$) of **3d**

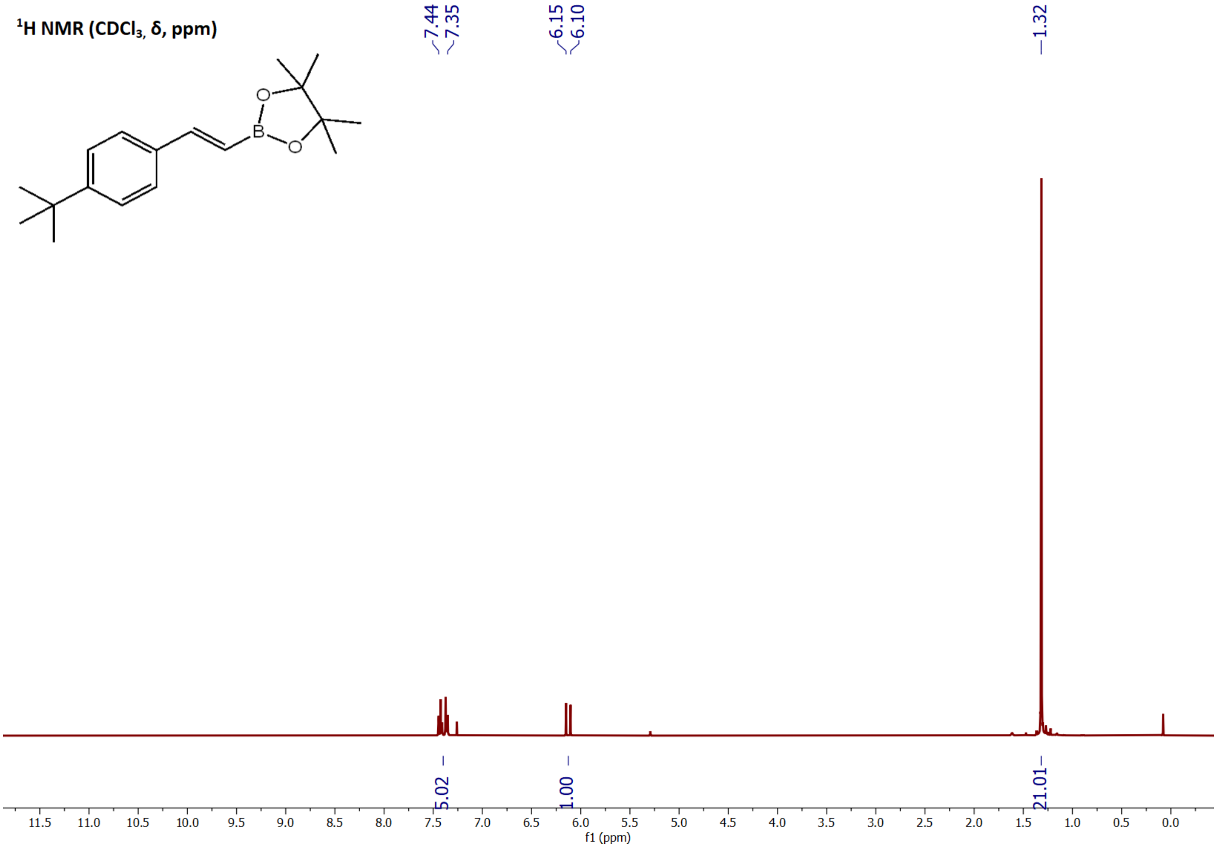

Figure 13. ^{1}H NMR (400 MHz, δ, $CDCl_3$) of **3e**

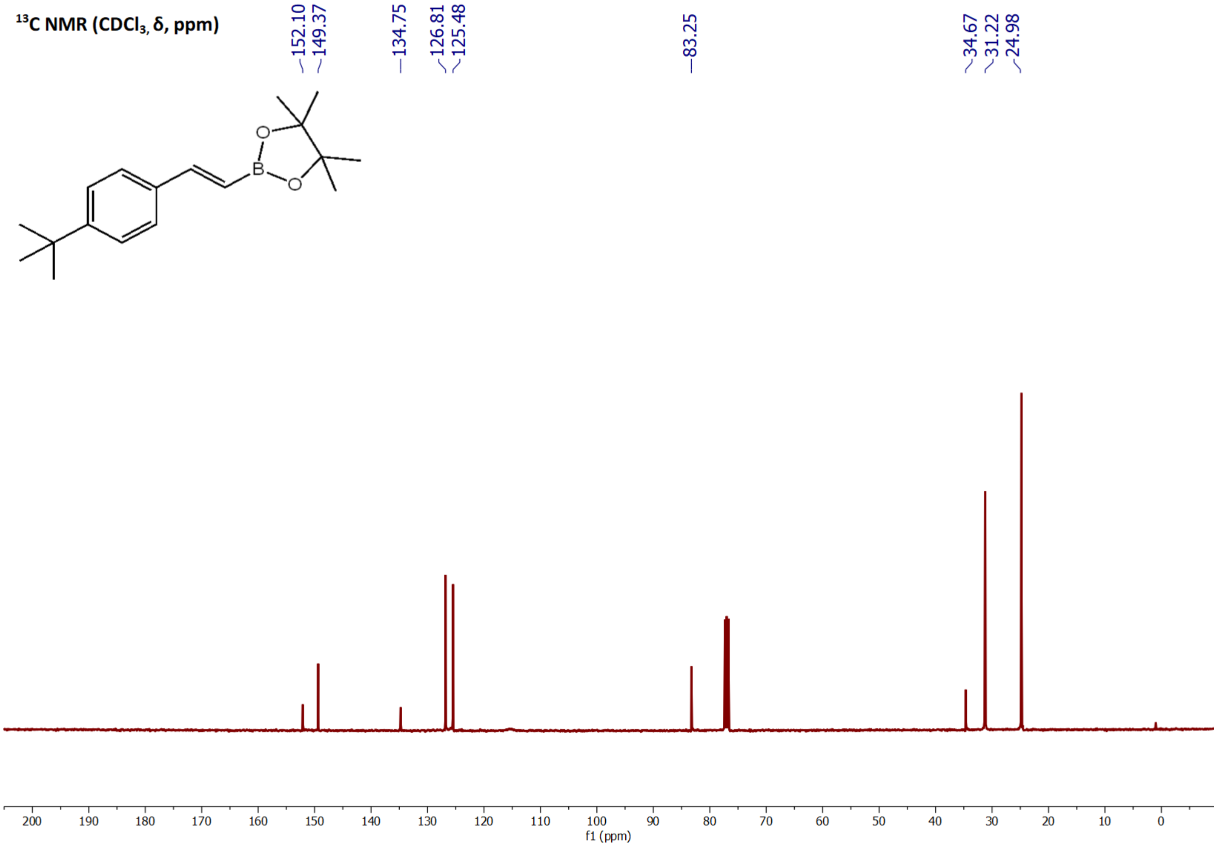

Figure 14. ^{1}H NMR (400 MHz, δ, $CDCl_3$) of **3e**

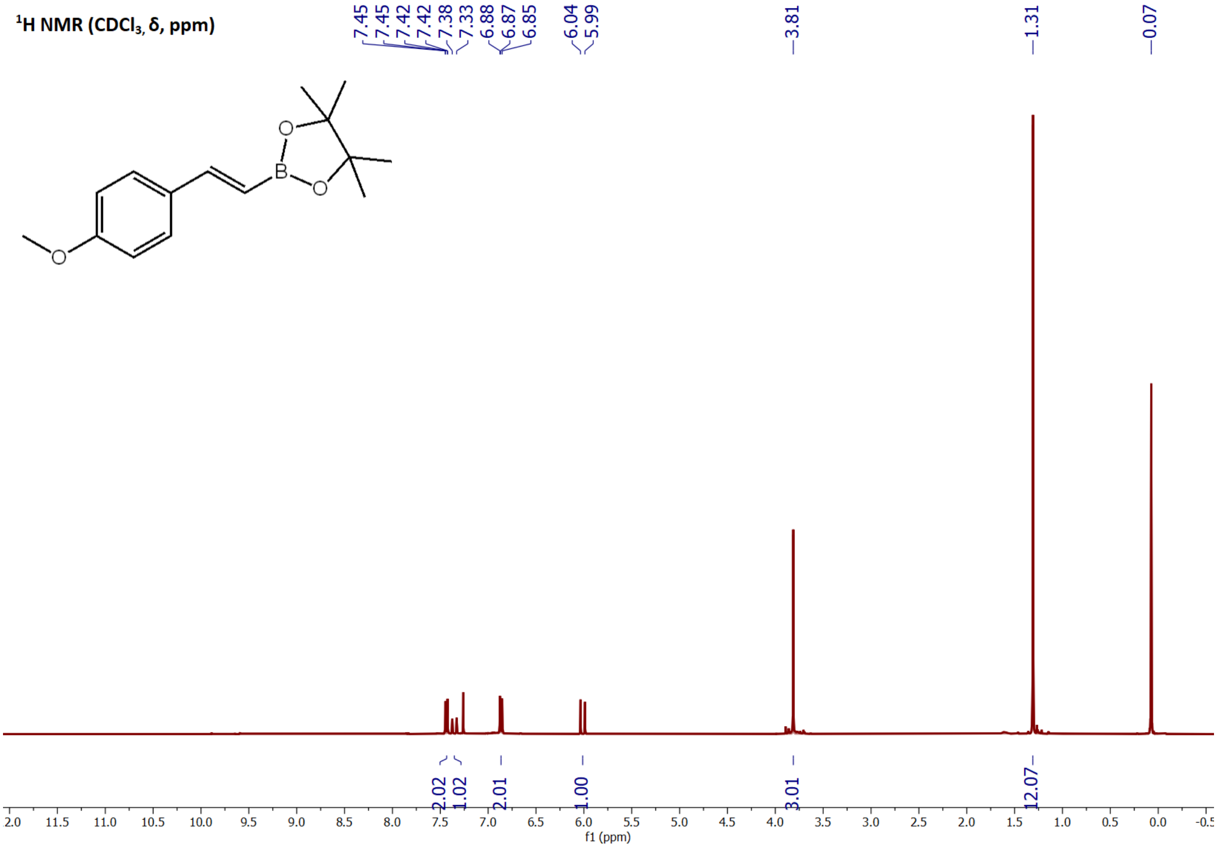

Figure 15. ^{1}H NMR (400 MHz, δ, $CDCl_3$) of **3f**

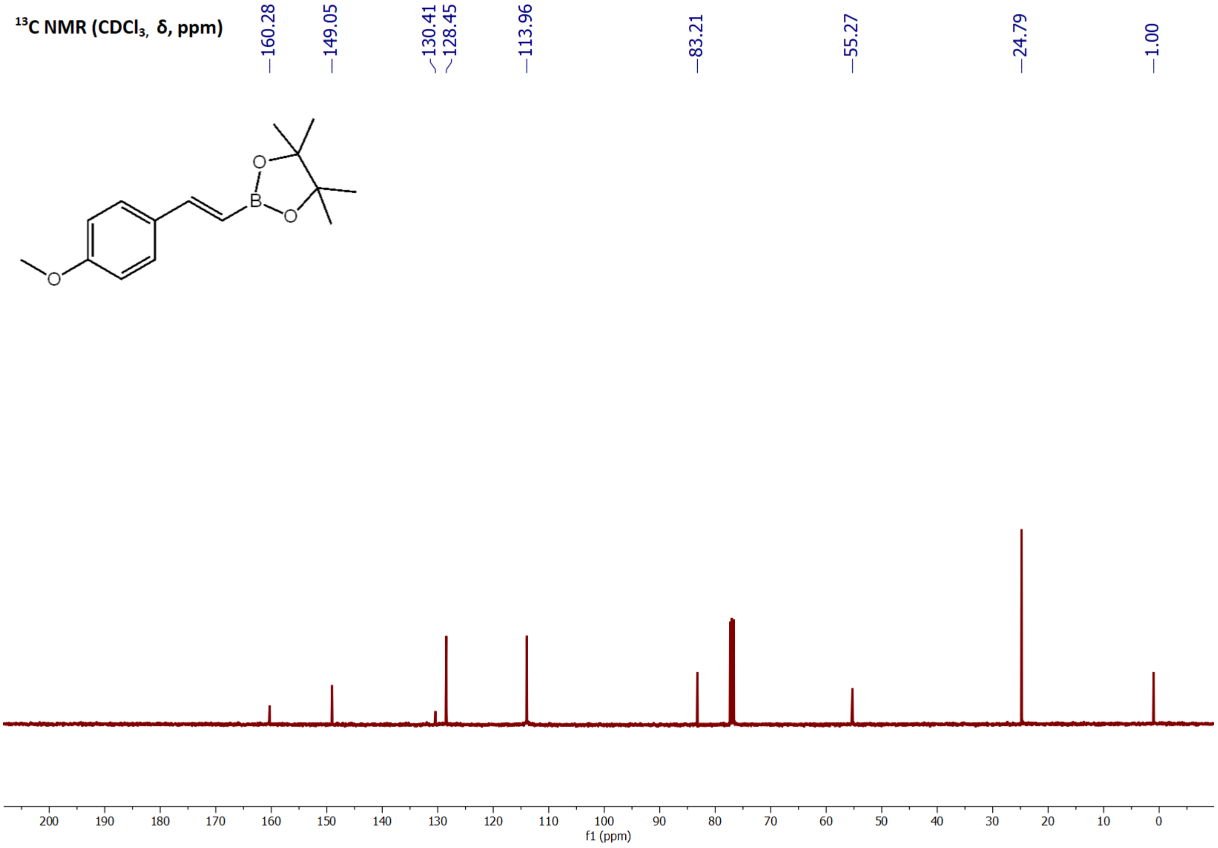

Figure 16. ^{13}C NMR (75 MHz, δ, $CDCl_3$) of **3f**

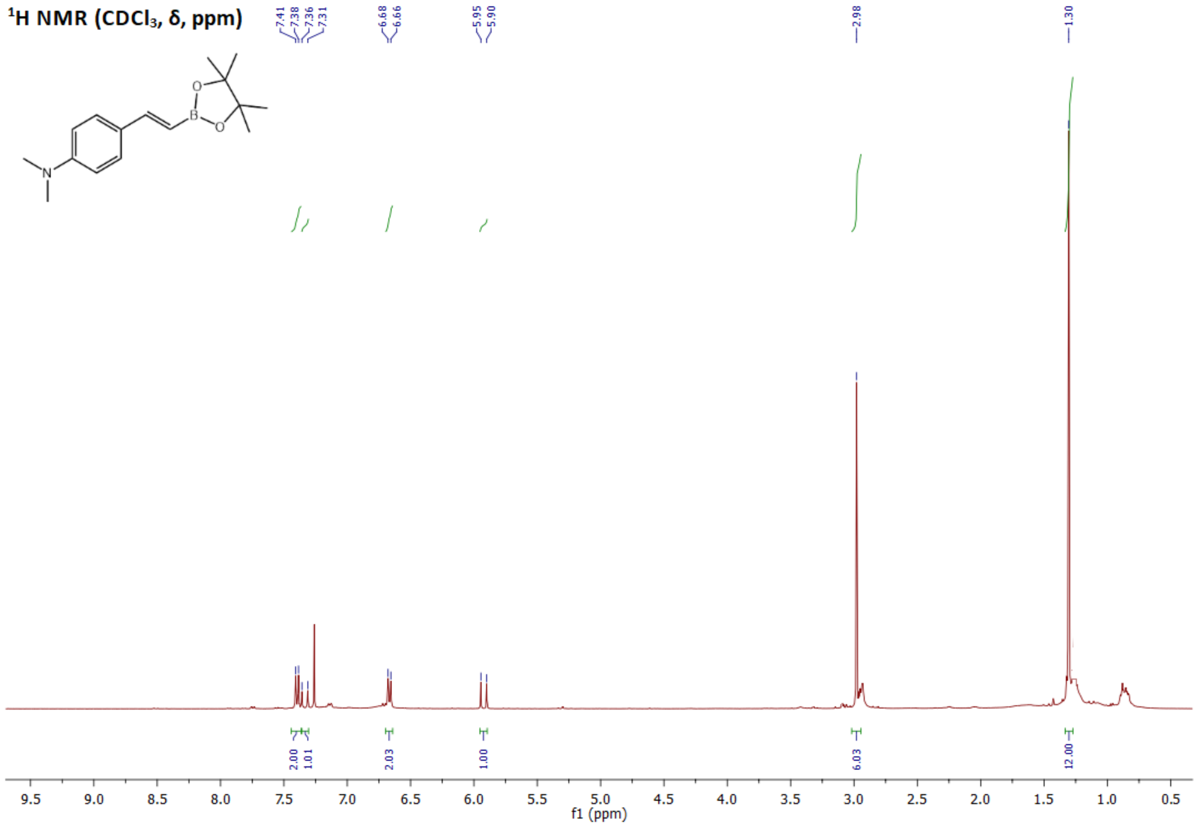

Figure 17. ^{1}H NMR (400 MHz, δ, $CDCl_3$) of **3g**

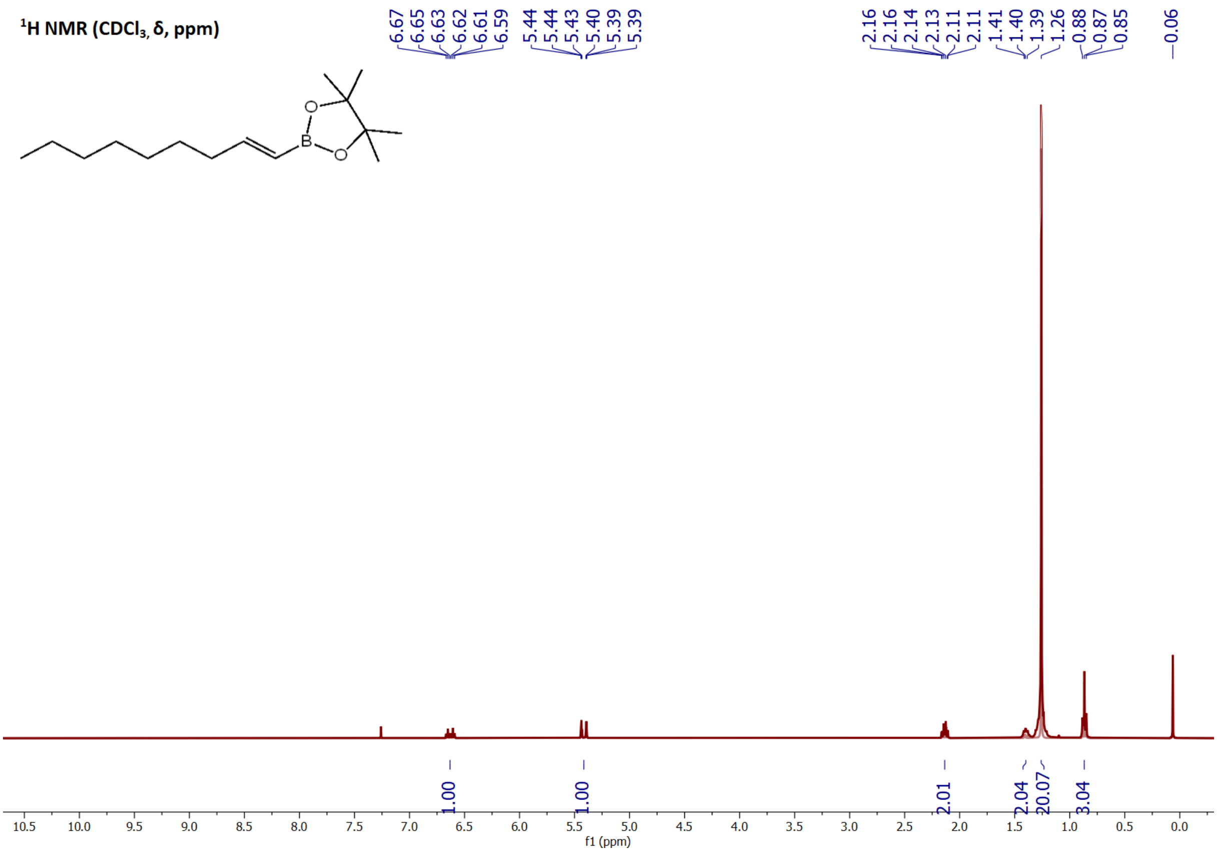

Figure 18. ^{1}H NMR (400 MHz, δ, $CDCl_3$) of **3j**

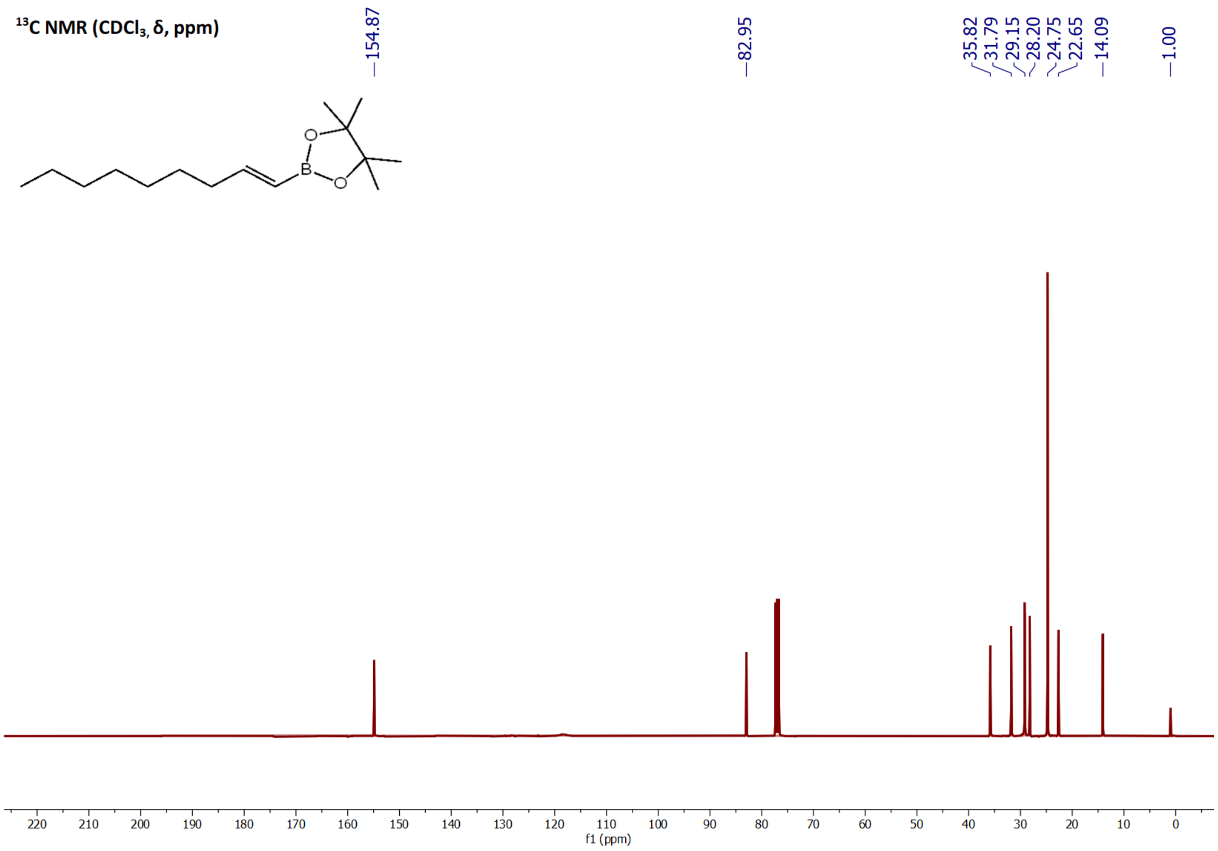

Figure 19. ^{13}C NMR (75 MHz, δ, $CDCl_3$) of **3j**

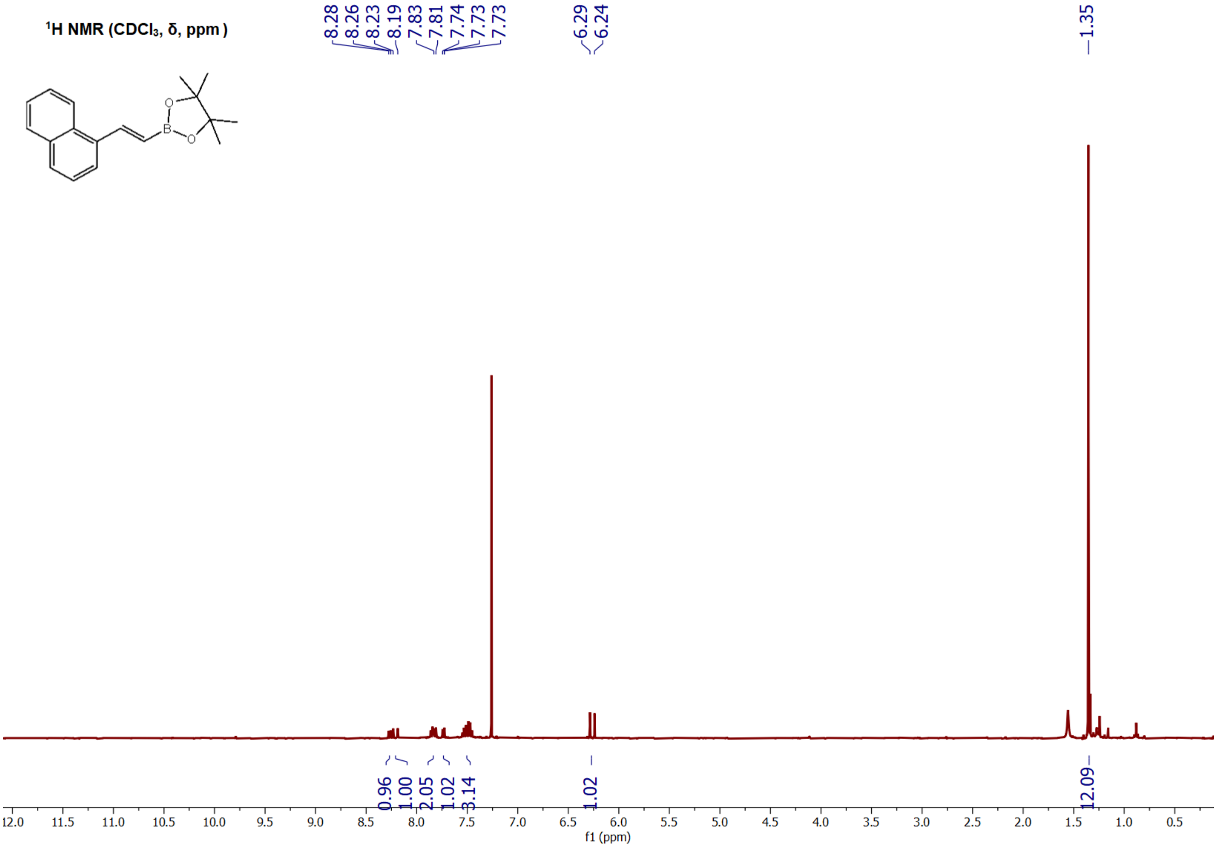

Figure 20. ^{1}H NMR (400 MHz, δ, $CDCl_3$) of **3l**

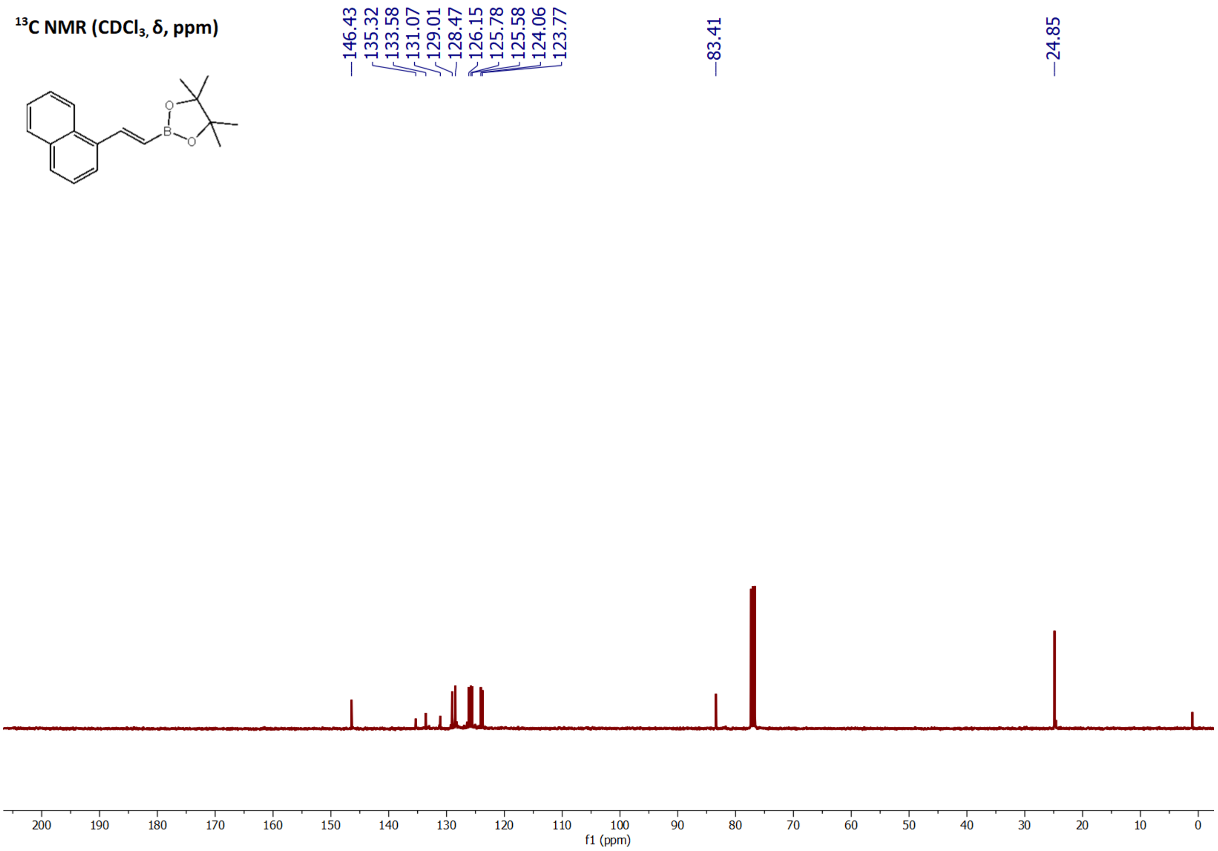

Figure 21. ^{13}C NMR (75 MHz, δ, $CDCl_3$) of **3l**

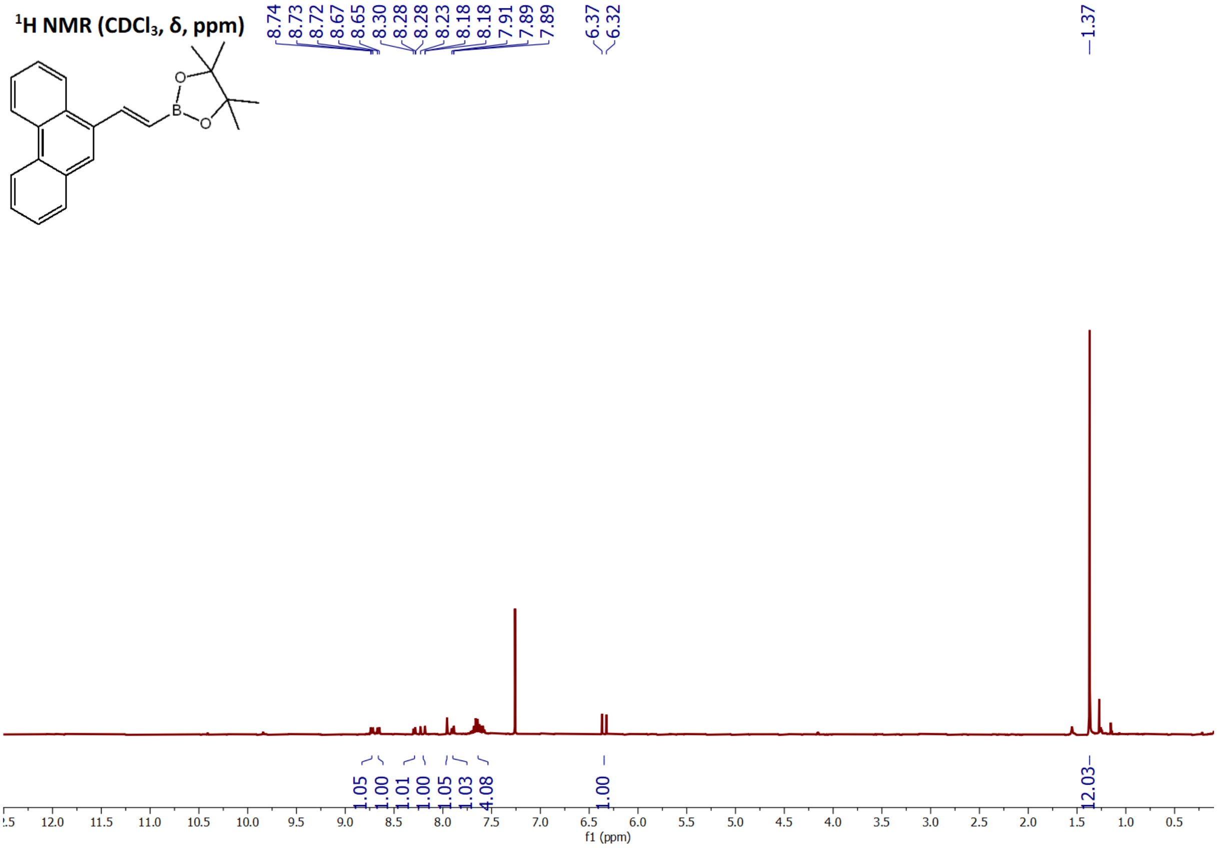

Figure 22. ^{1}H NMR (400 MHz, δ, $CDCl_3$) of **3m**

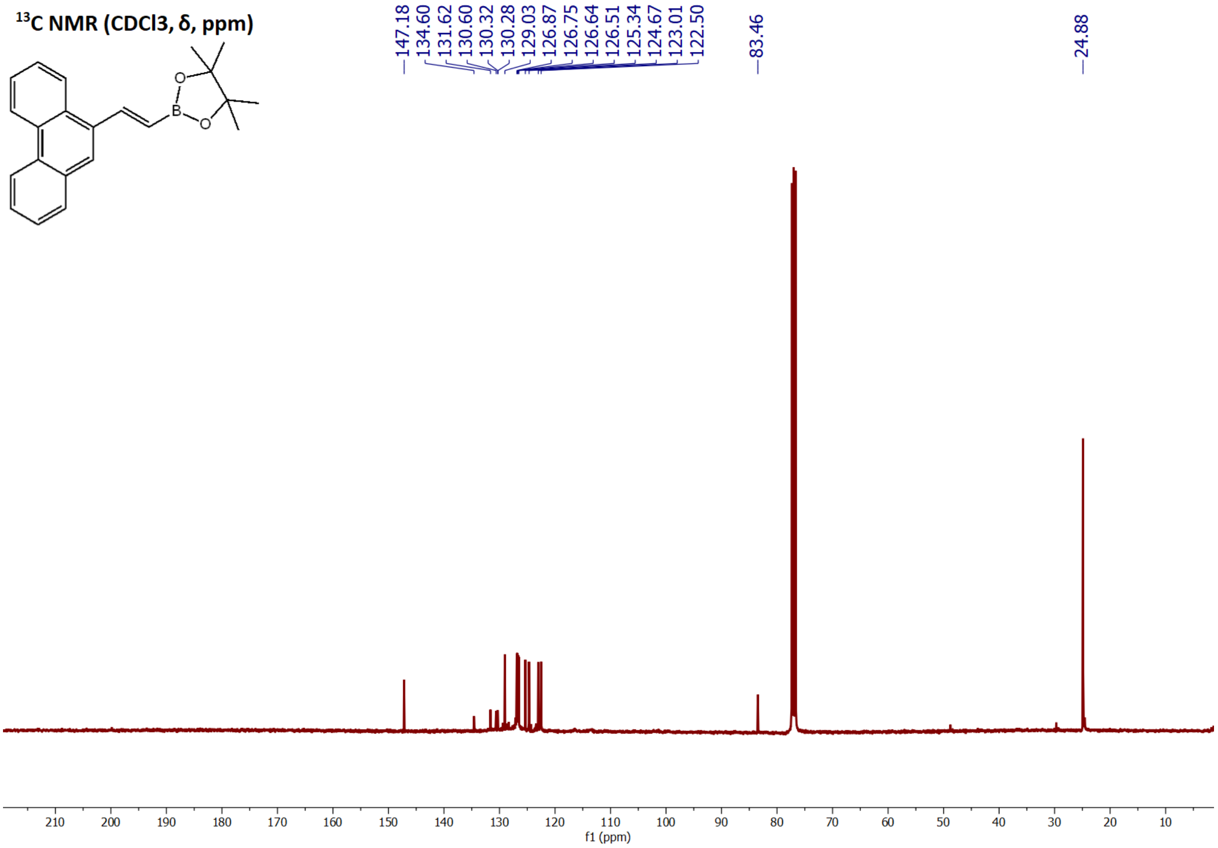

Figure 23. ^{13}C NMR (75 MHz, δ, $CDCl_3$) of **3m**

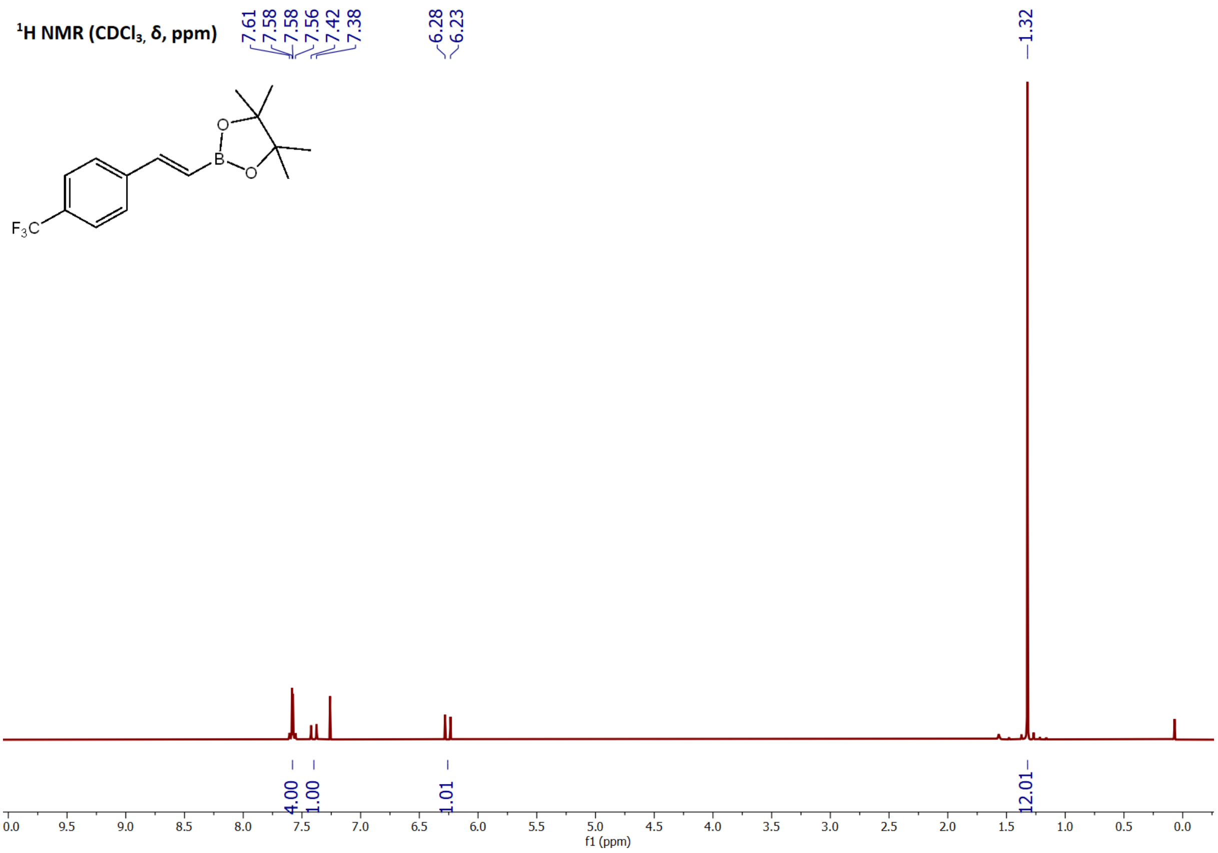

Figure 24. ^{1}H NMR (400 MHz, δ, $CDCl_3$) of **3n**

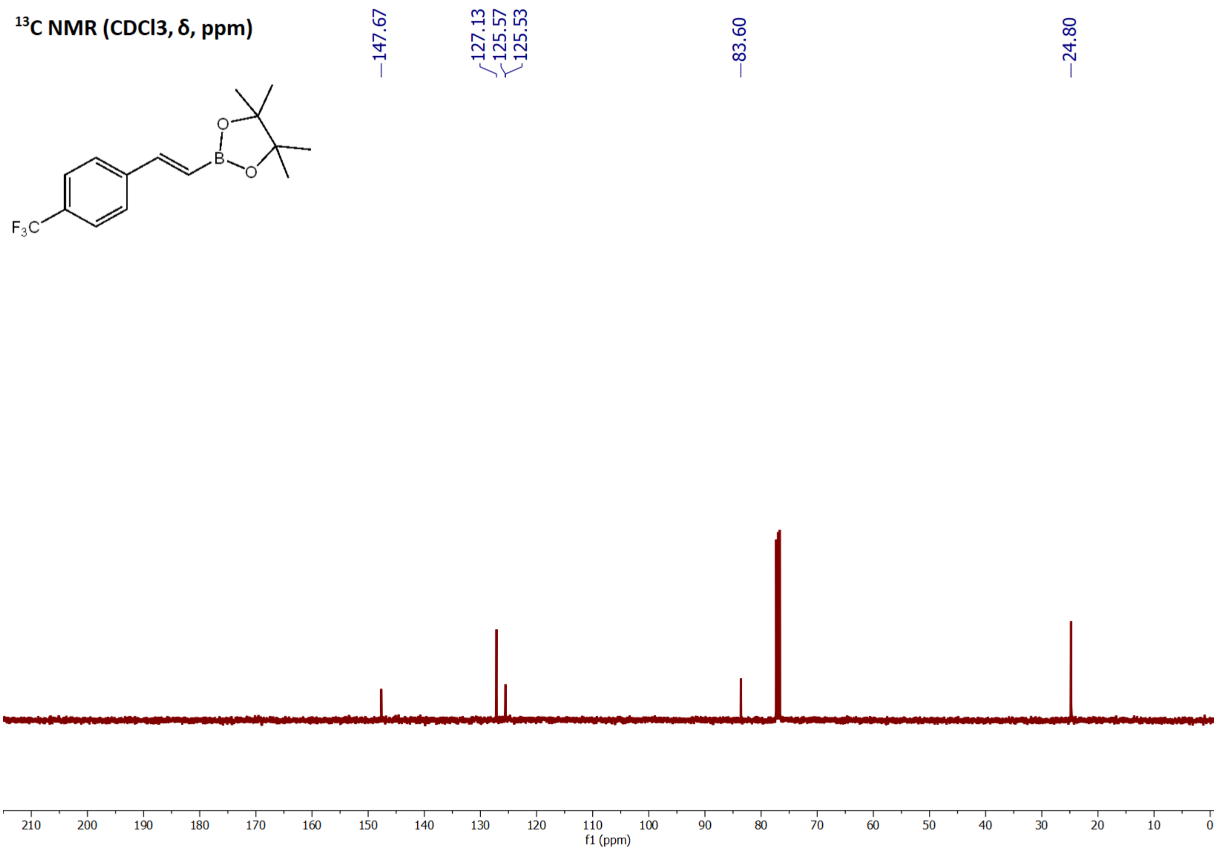

Figure 25. ^{13}C NMR (75 MHz, δ, $CDCl_3$) of **3n**

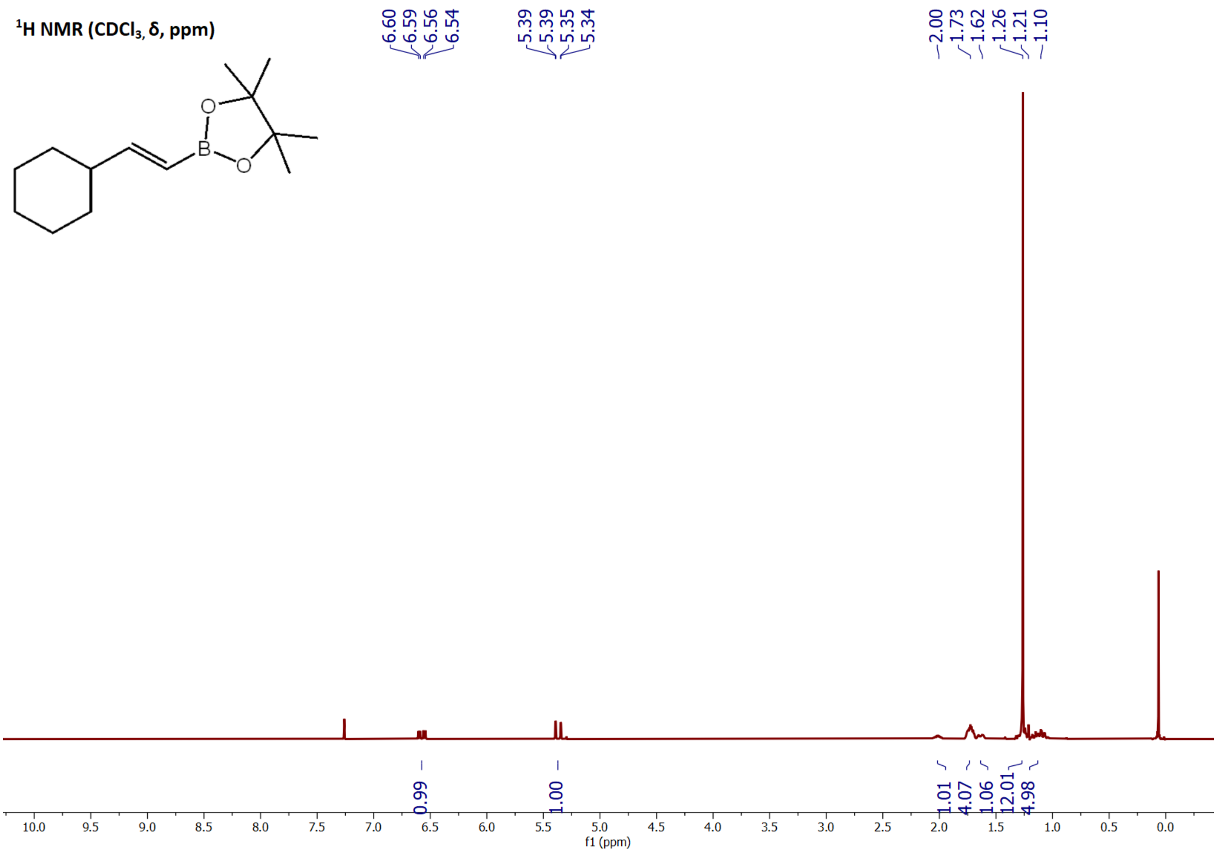

Figure 26. ^{1}H NMR (400 MHz, δ, $CDCl_3$) of **3o**

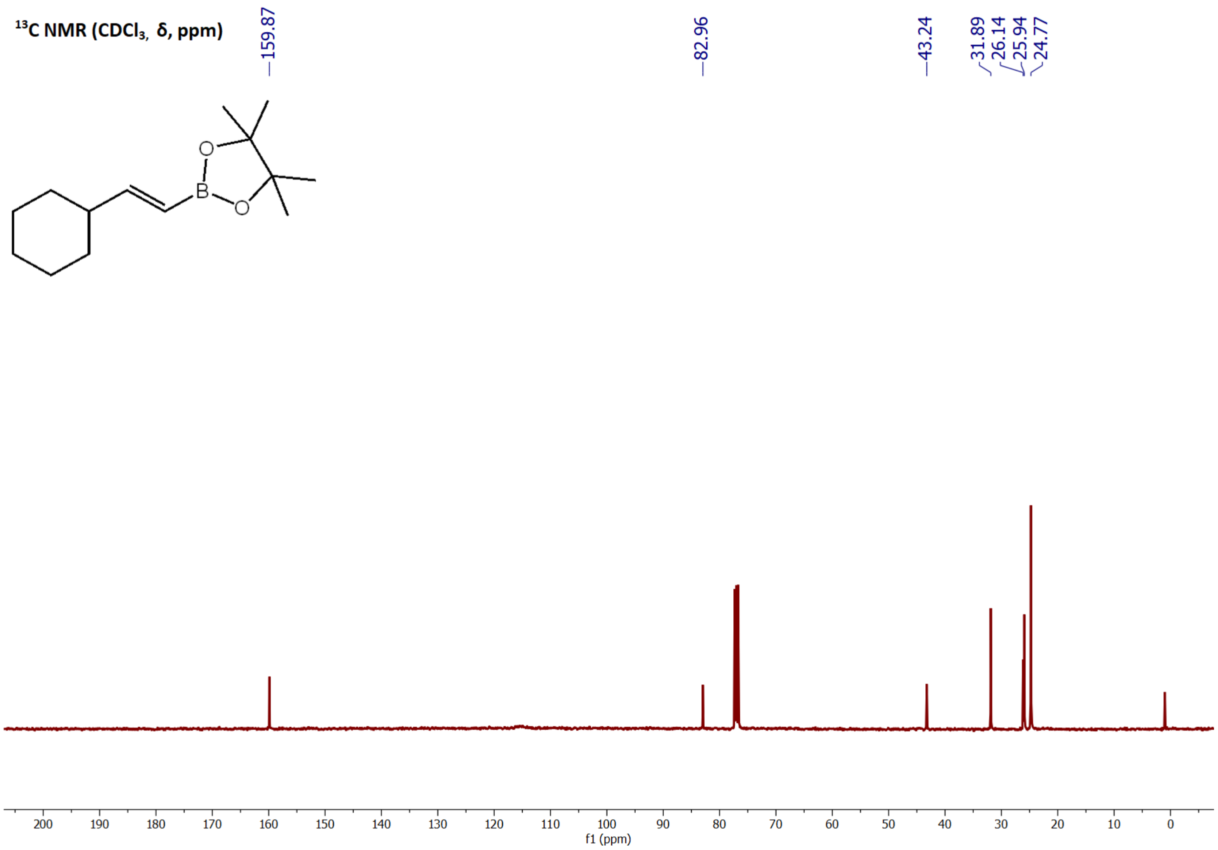

Figure 27. ^{13}C NMR (75 MHz, δ, $CDCl_3$) of **3o**

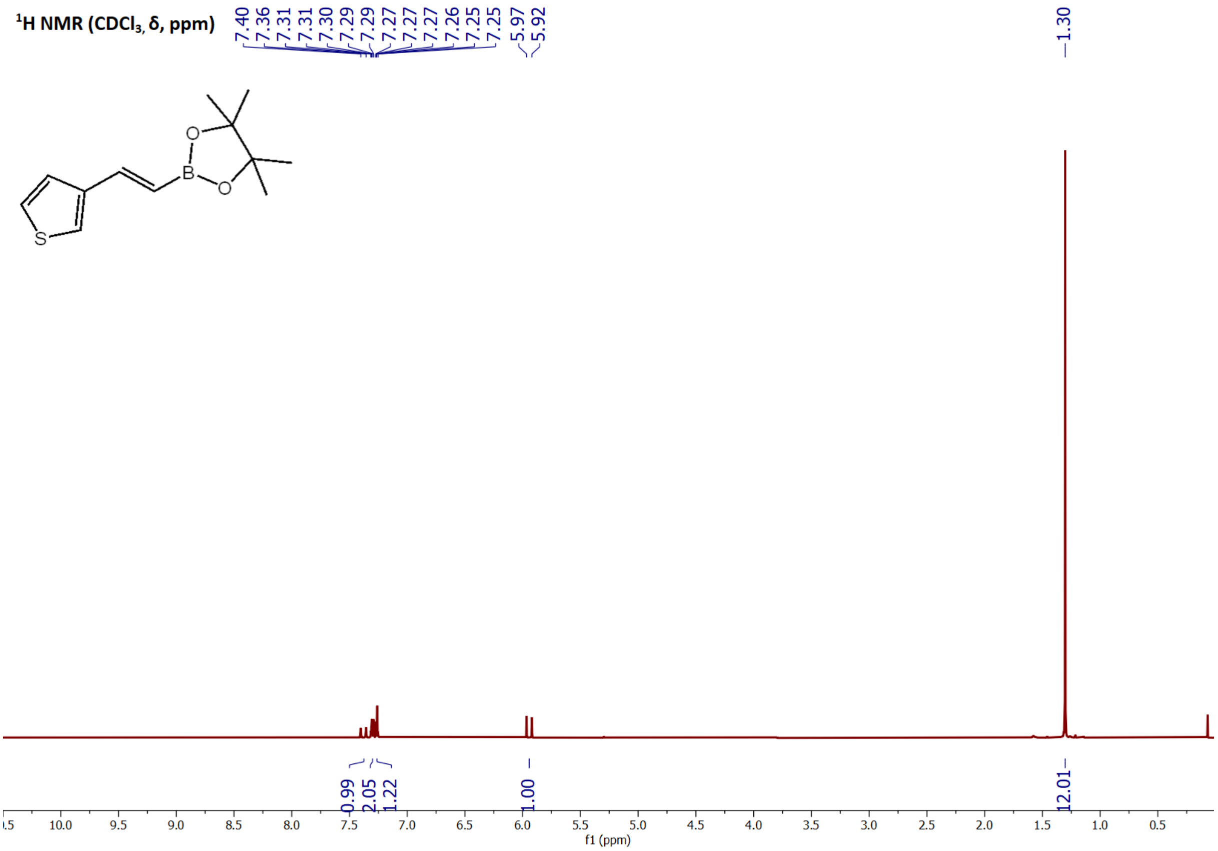

Figure 28. ^{1}H NMR (400 MHz, δ, $CDCl_3$) of **3p**

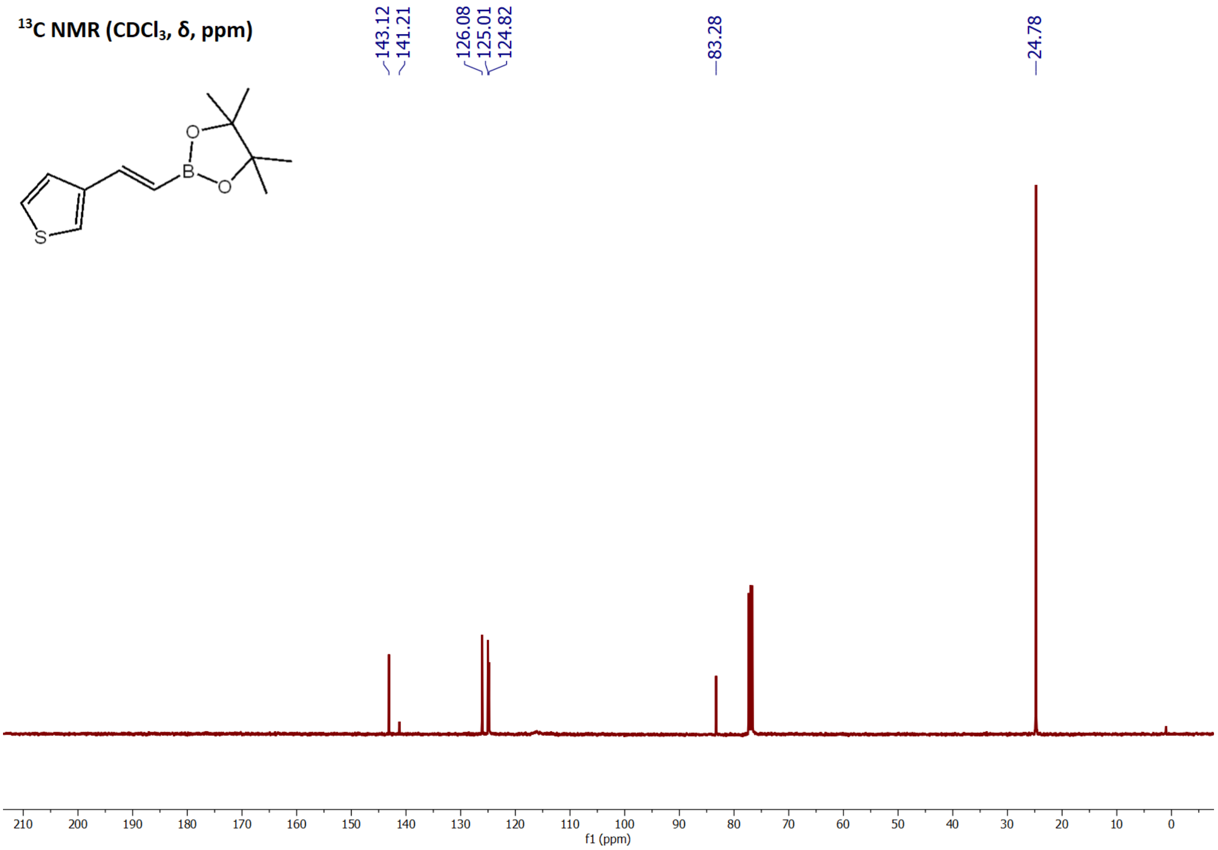

Figure 29. ^{13}C NMR (75 MHz, δ, $CDCl_3$) of **3p**

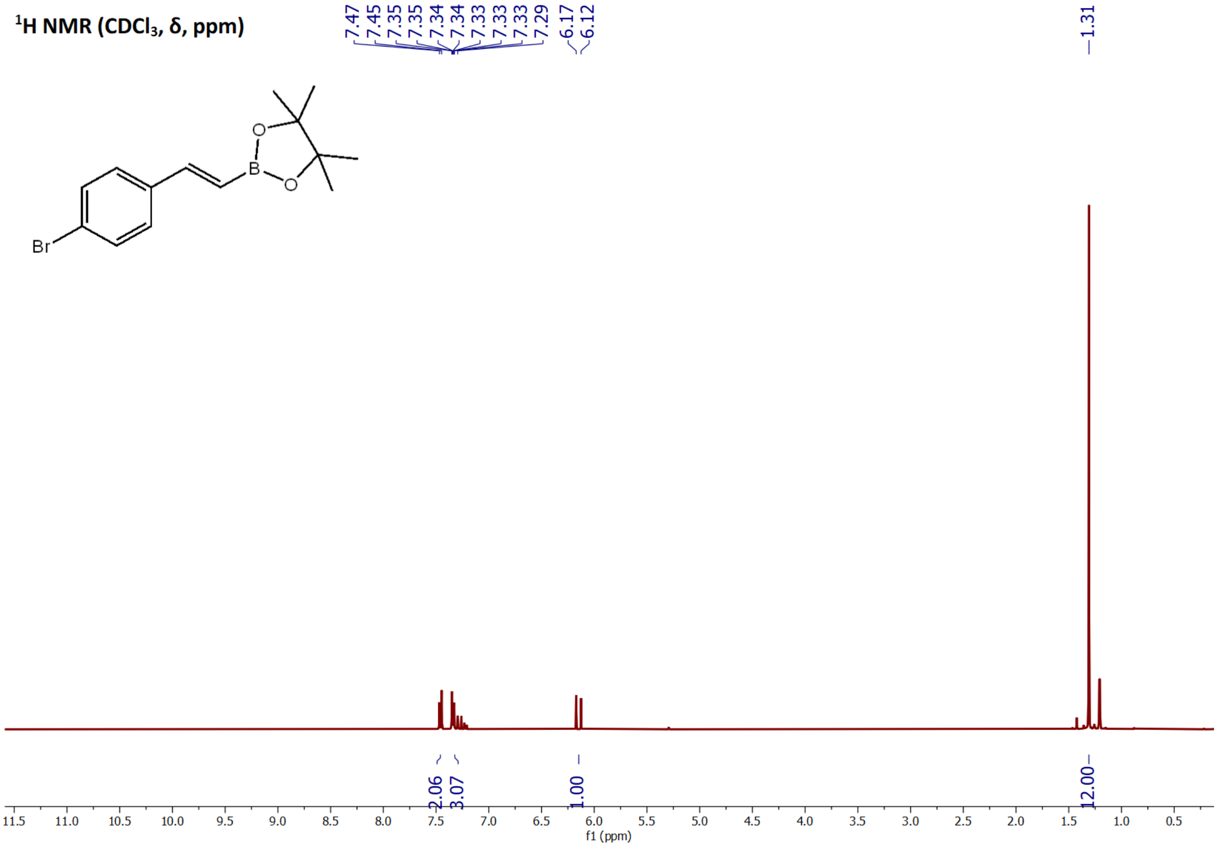

Figure 30. ^{1}H NMR (400 MHz, δ, $CDCl_3$) of **3r**

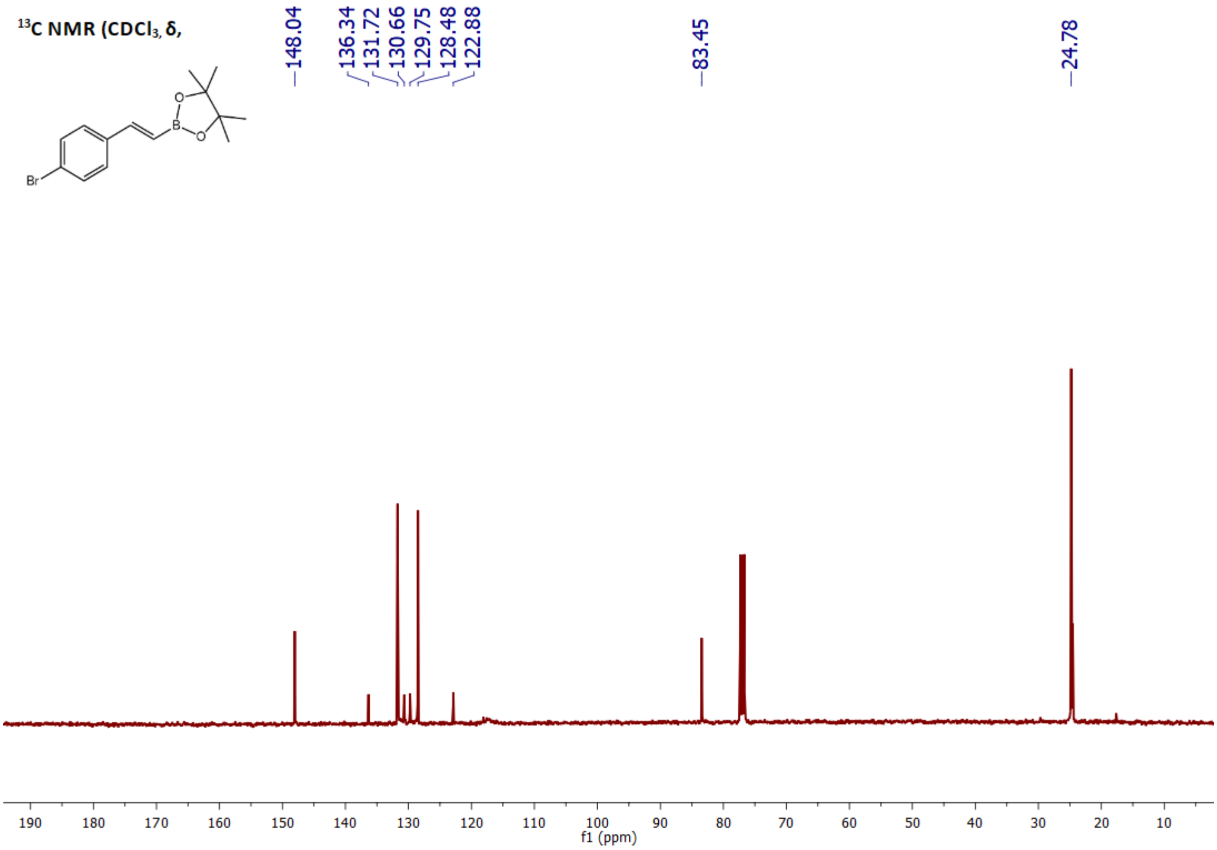

Figure 31. ^{13}C NMR (75 MHz, δ, $CDCl_3$) of **3r**

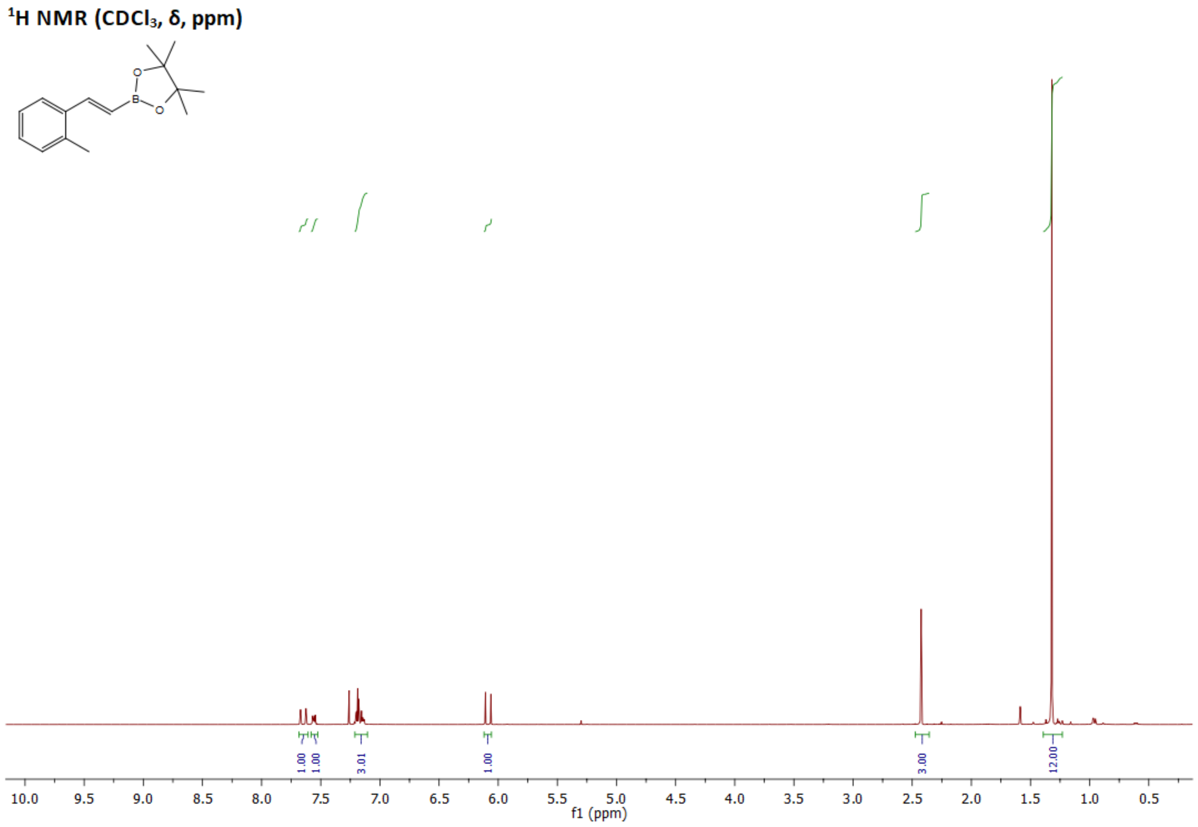

Figure 32. ^{1}H NMR (400 MHz, δ, CDCl$_3$) of **3s**

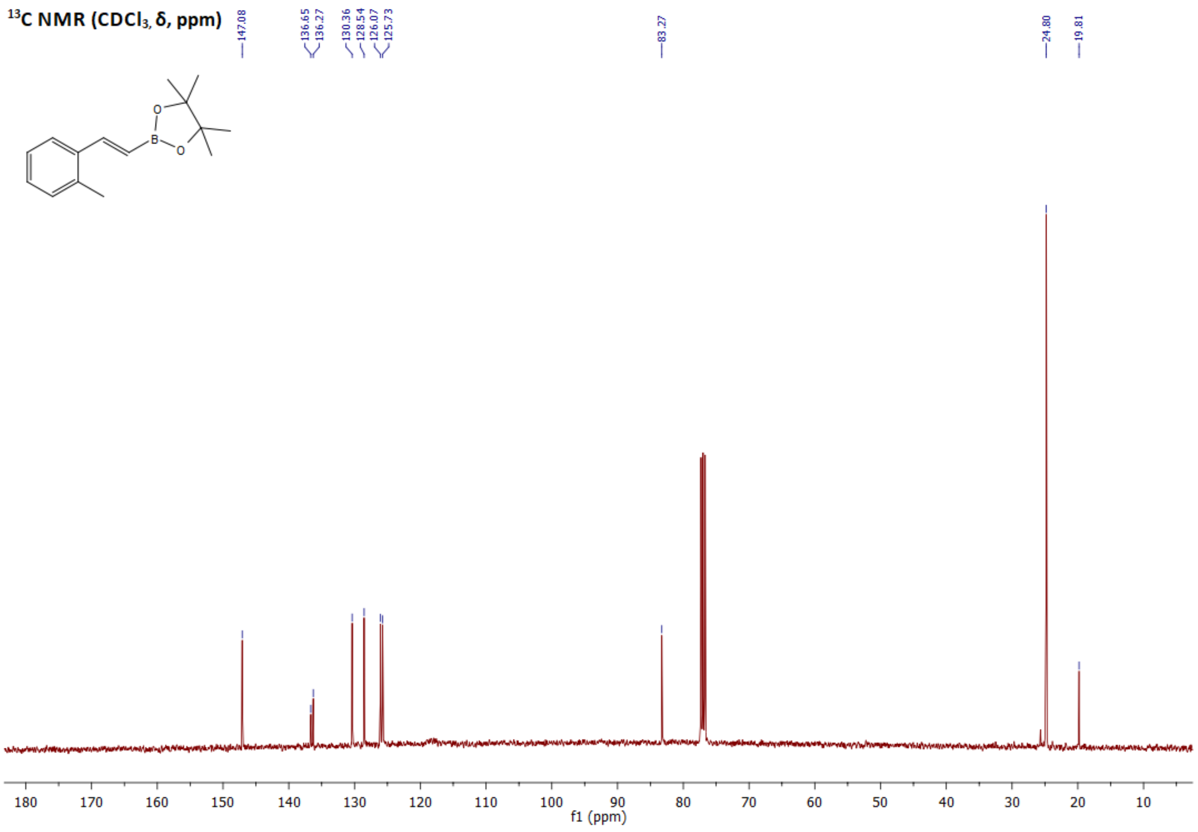

Figure 33. ^{13}C NMR (75 MHz, δ, CDCl$_3$) of **3s**

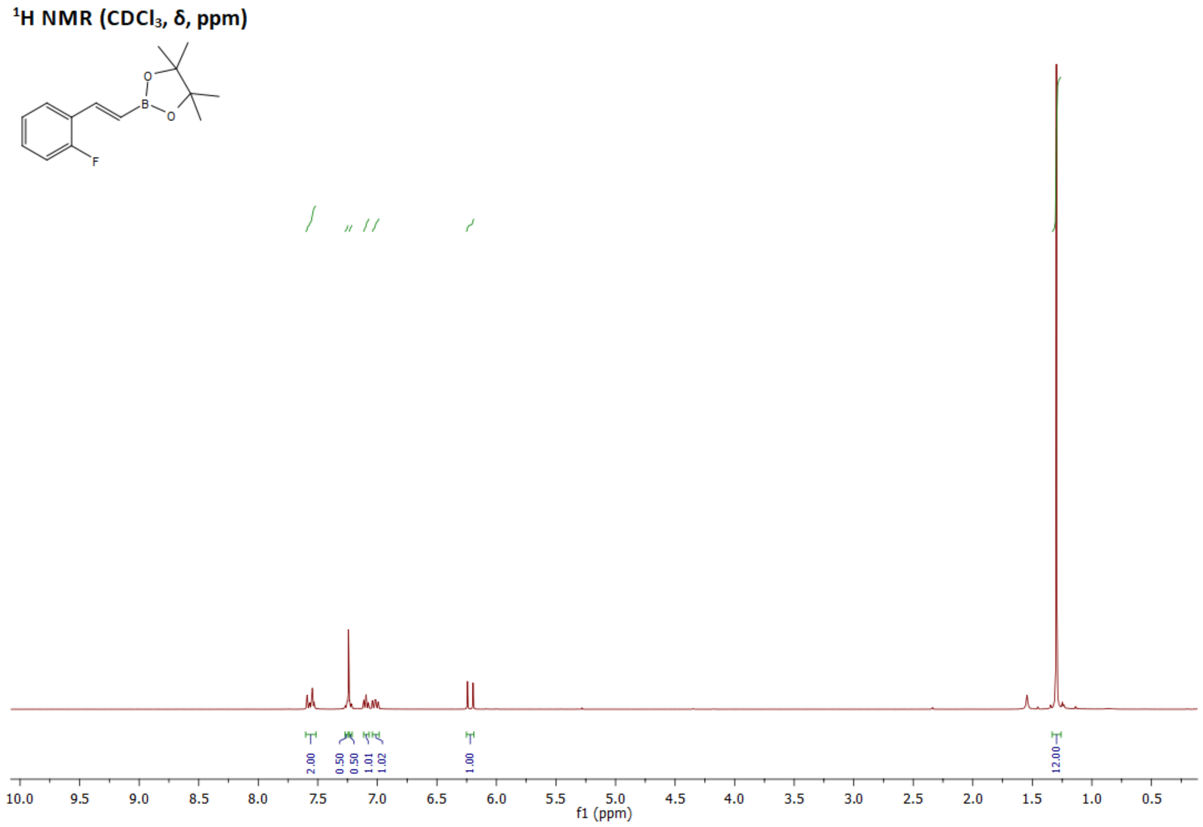

Figure 34. ^{1}H NMR (400 MHz, δ, $CDCl_3$) of **3t**

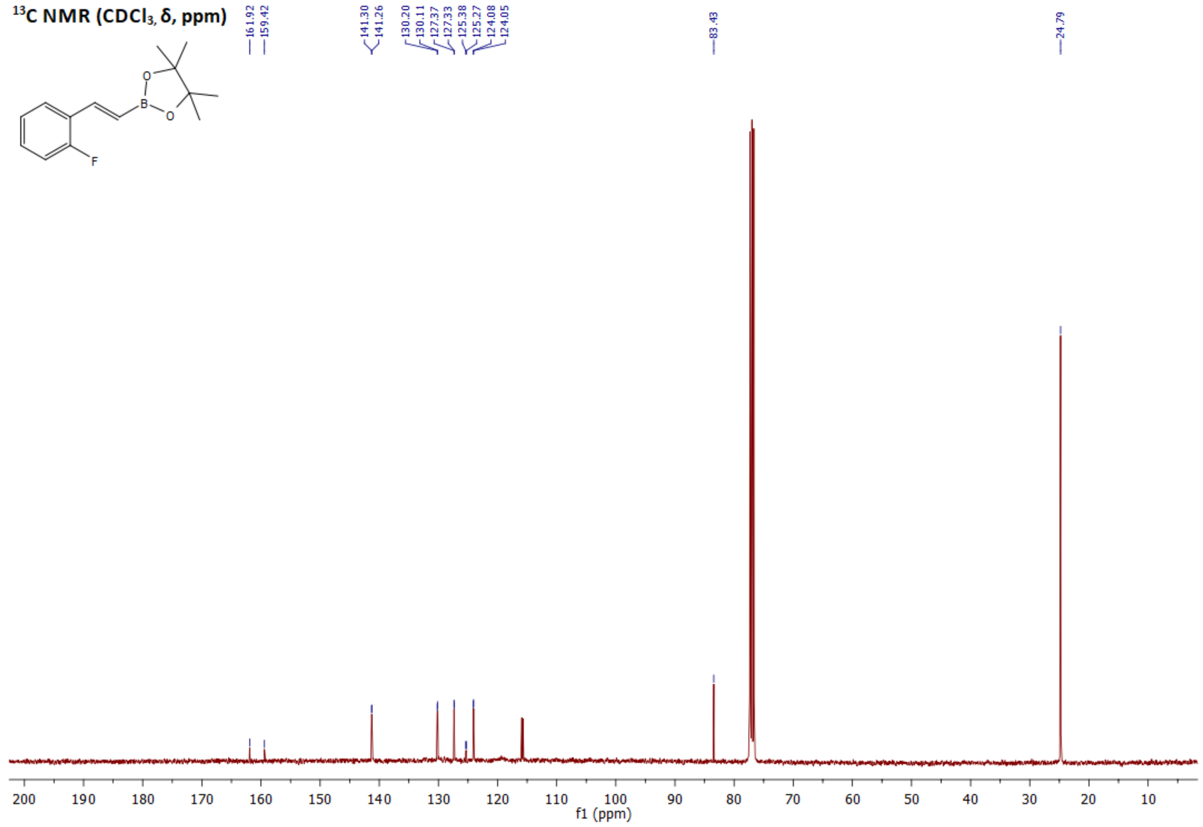

Figure 35. ^{13}C NMR (75 MHz, δ, $CDCl_3$) of **3t**

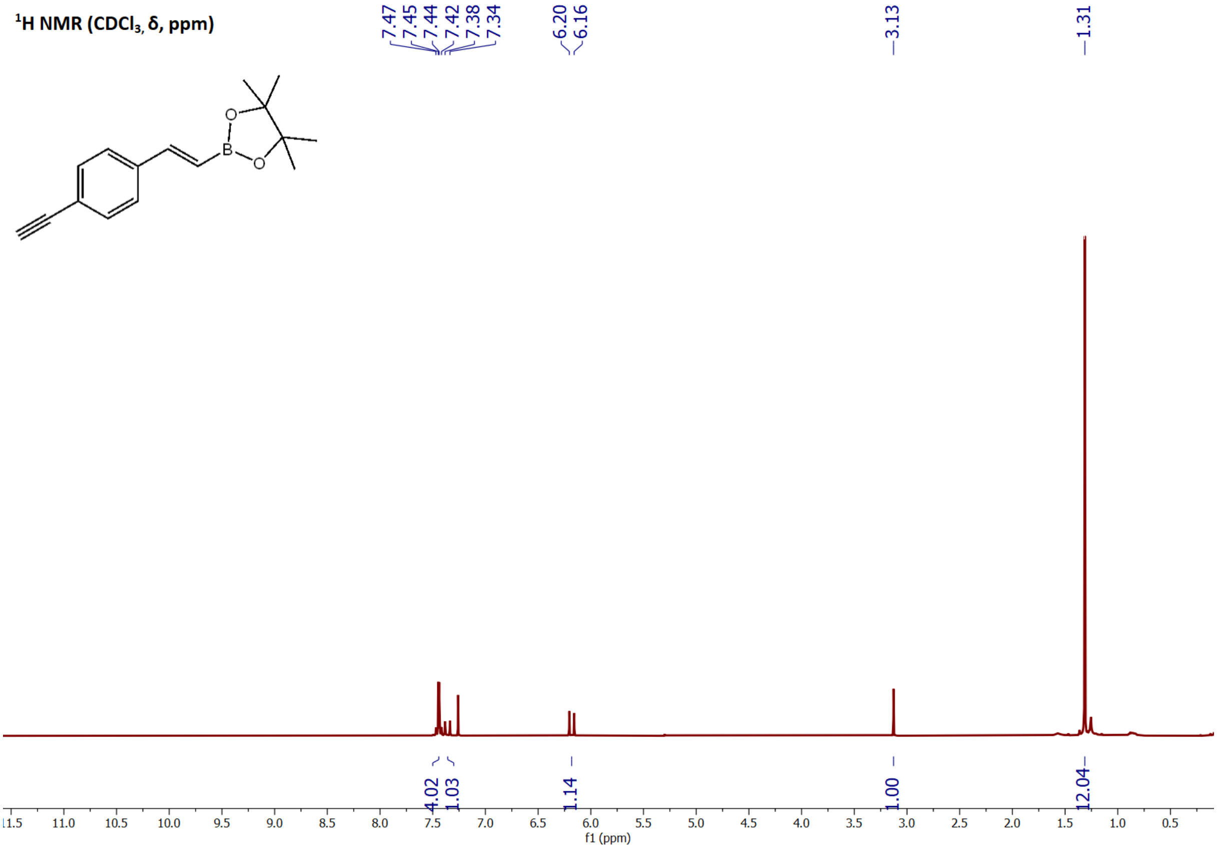

Figure 36. ^{1}H NMR (400 MHz, δ, $CDCl_3$) of **5a**

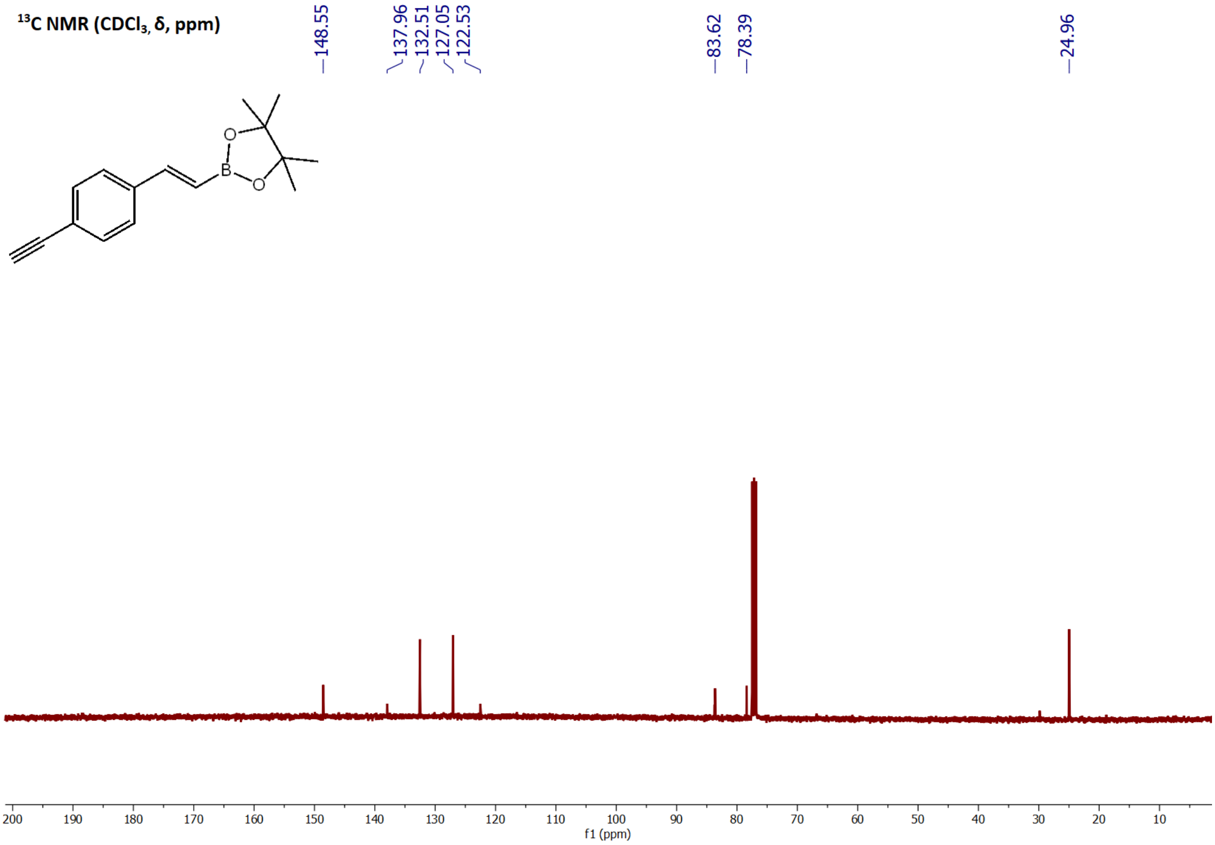

Figure 37. ^{13}C NMR (75 MHz, δ, $CDCl_3$) of **5a**

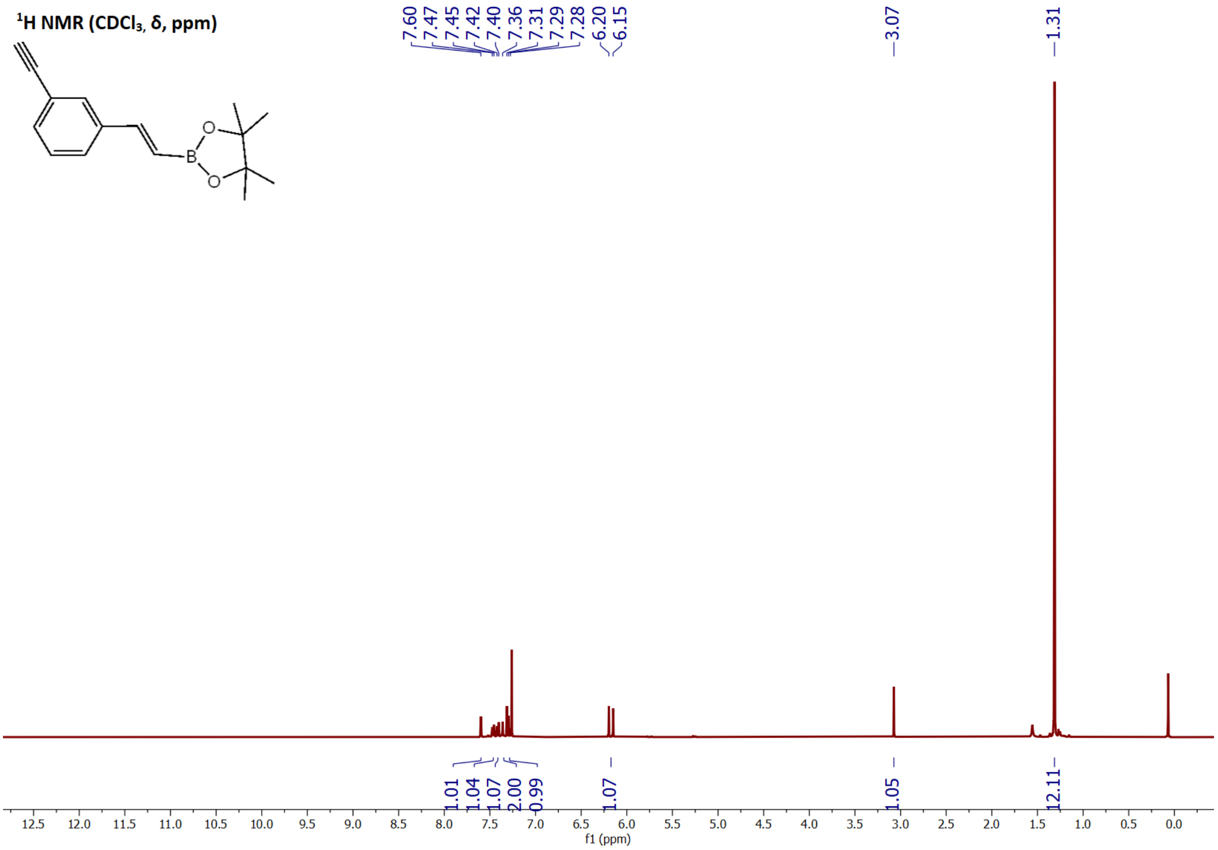

Figure 38. ^{1}H NMR (400 MHz, δ, $CDCl_3$) of **5b**

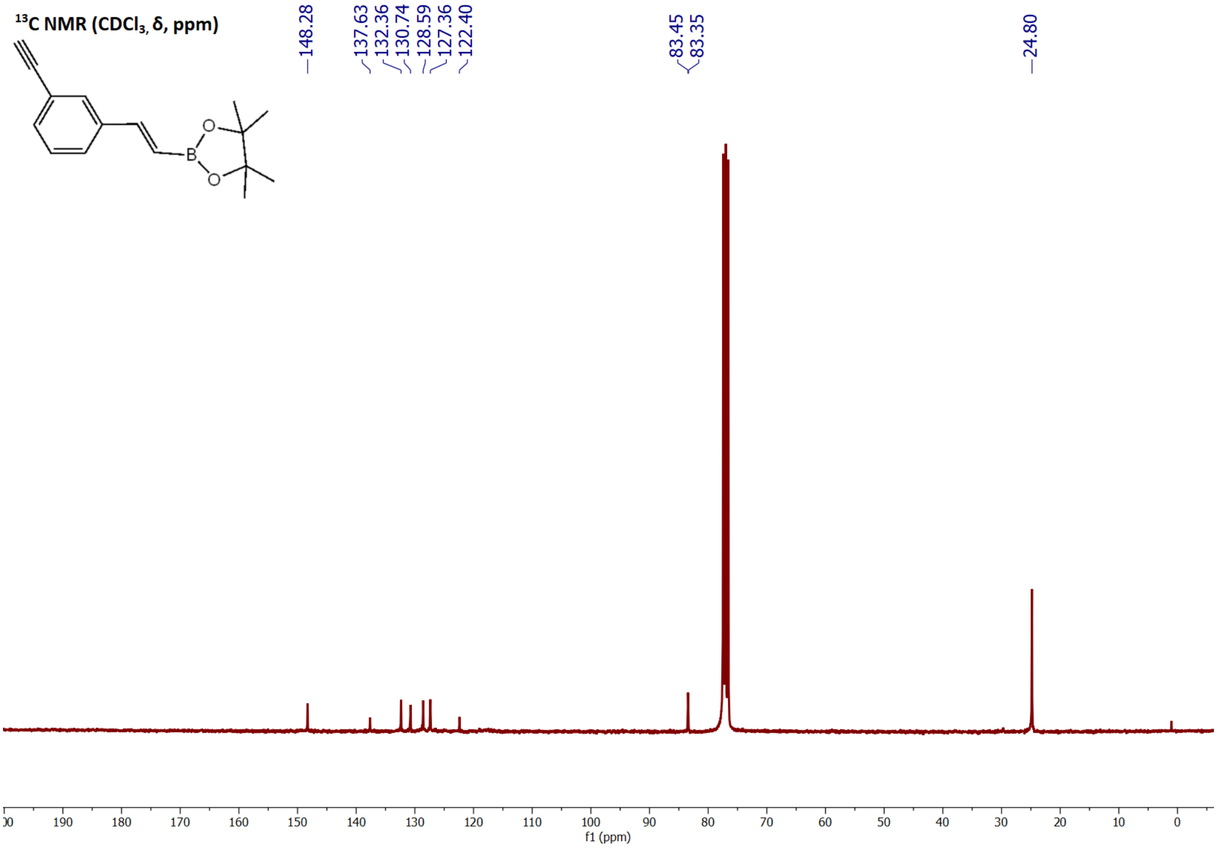

Figure39. ^{13}C NMR (75 MHz, δ, $CDCl_3$) of **5b**

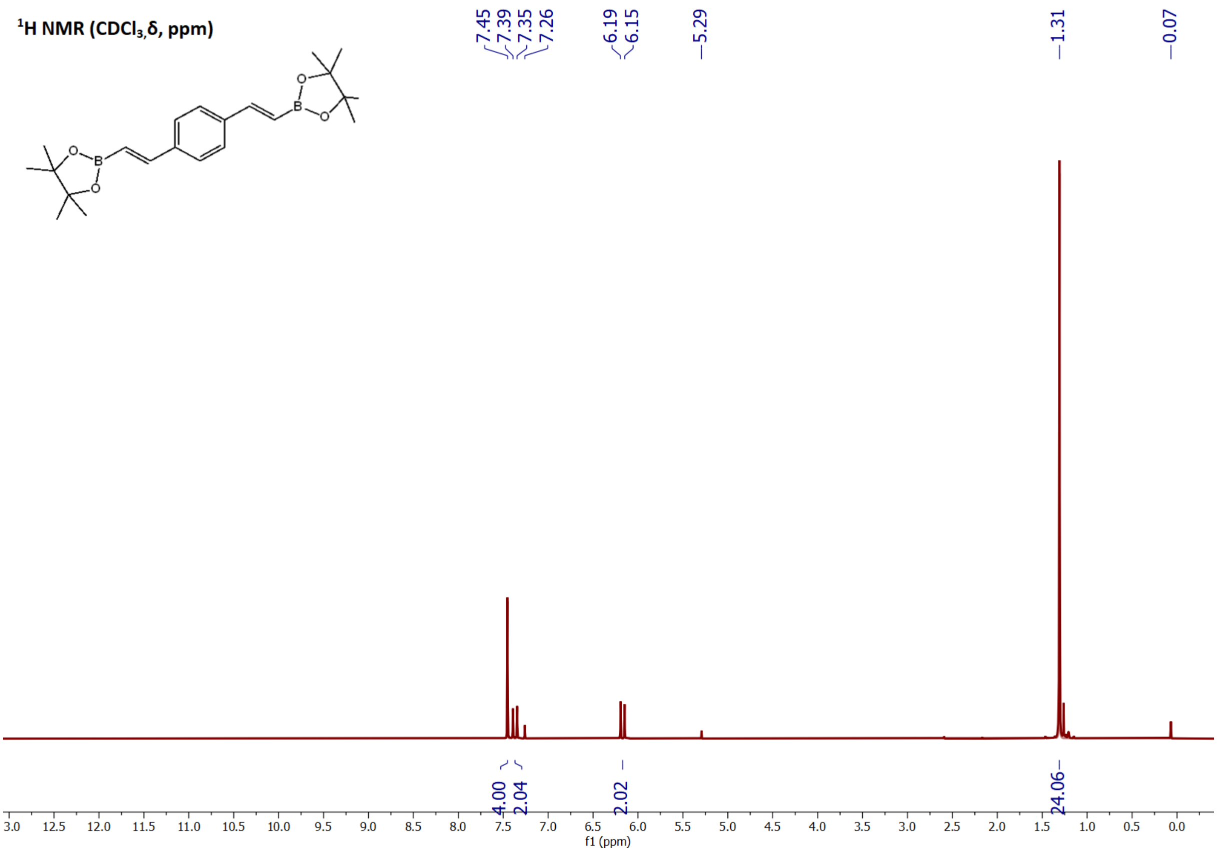

Figure 40. ^{1}H NMR (400 MHz, δ, $CDCl_3$) of **6a**

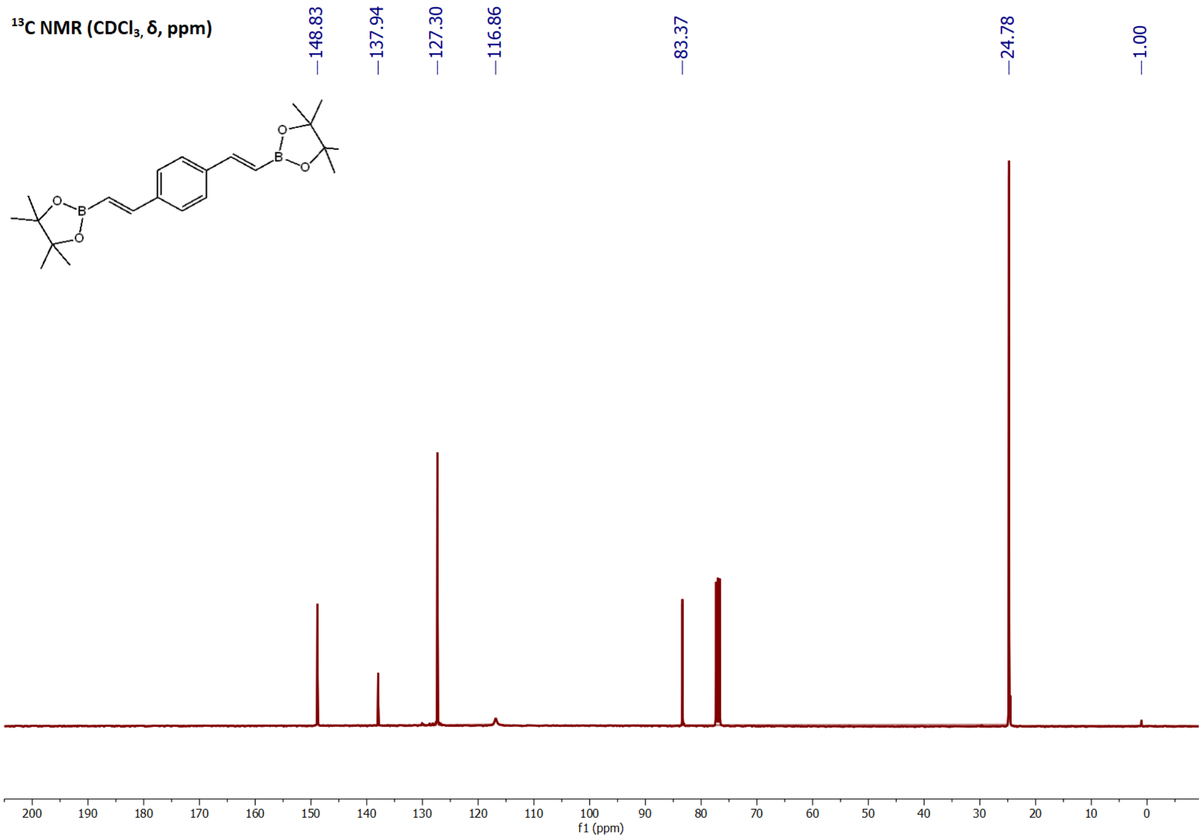

Figure 41. ^{13}C NMR (75 MHz, δ, $CDCl_3$) of **6a**

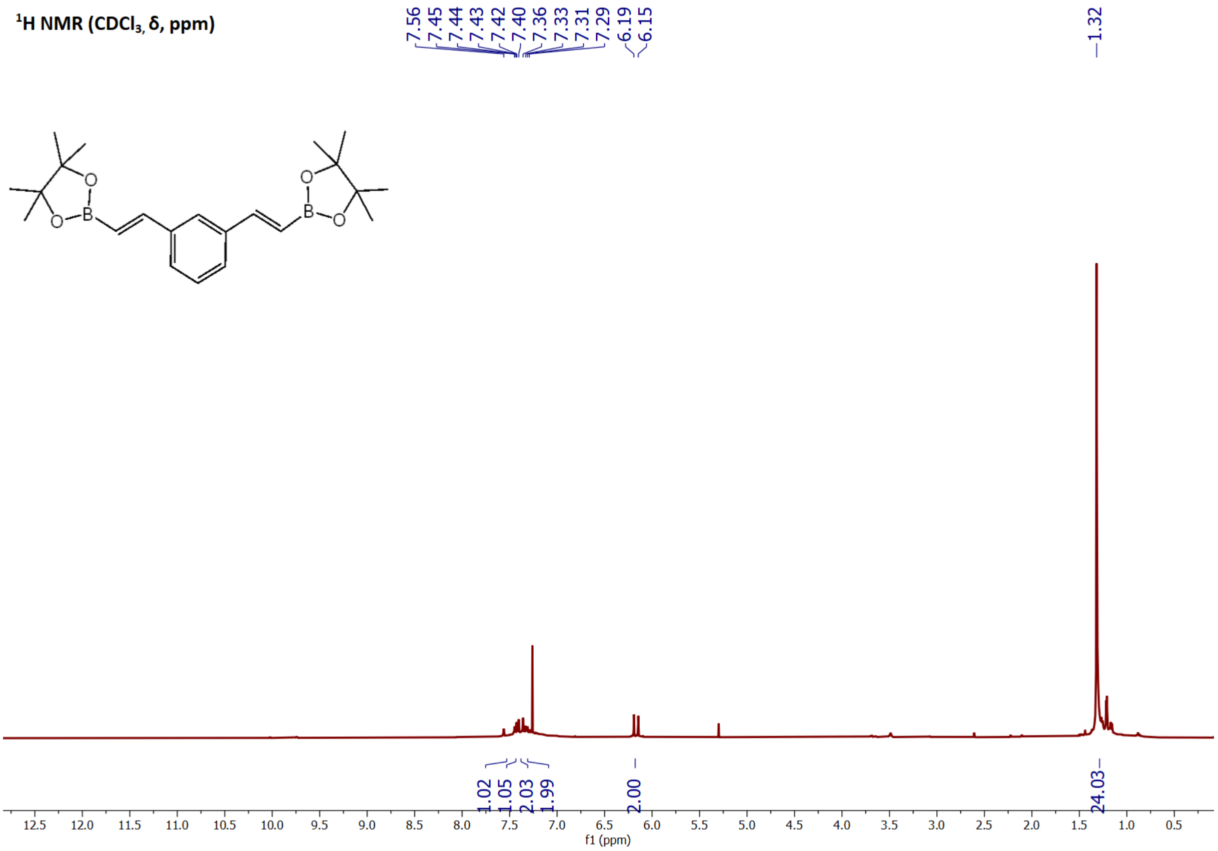

Figure 42. ^{1}H NMR (400 MHz, δ, $CDCl_3$) of **6b**

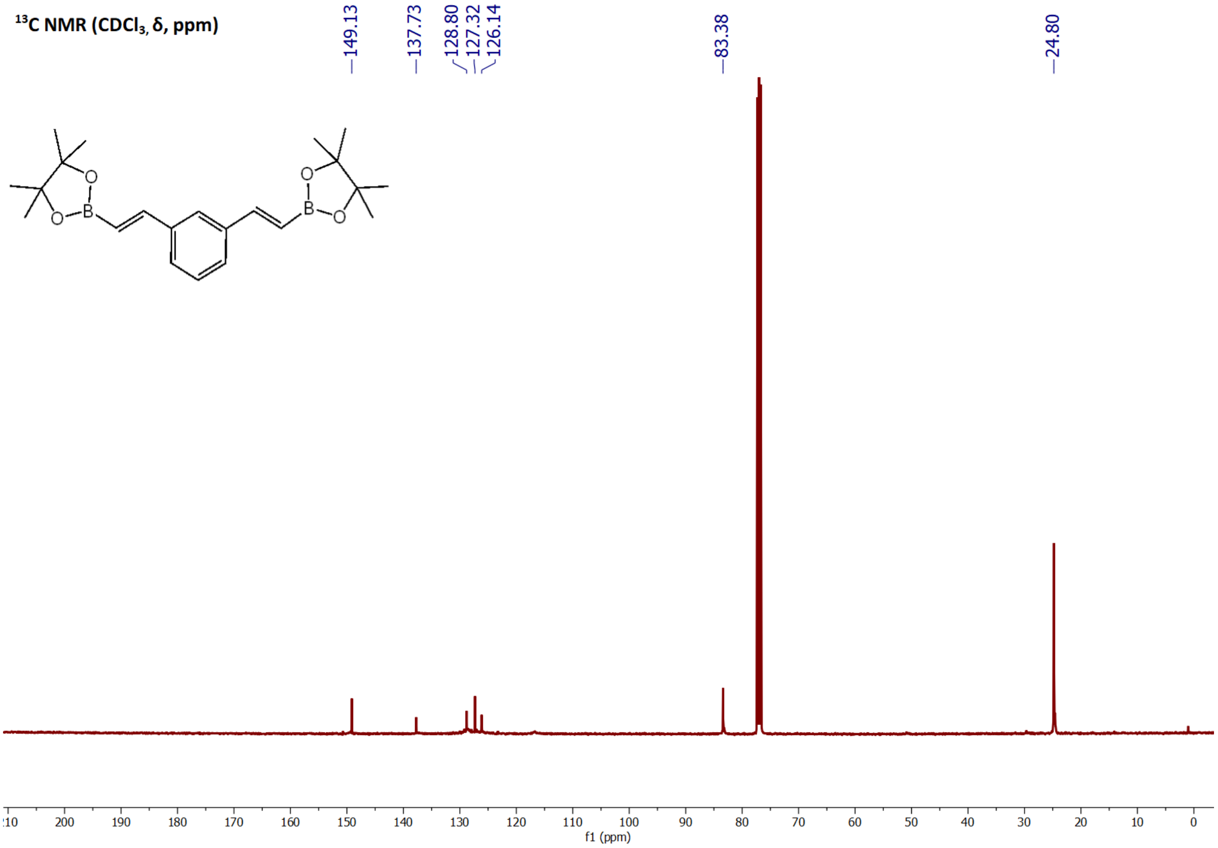

Figure 43. ^{13}C NMR (75 MHz, δ, $CDCl_3$) of **6b**

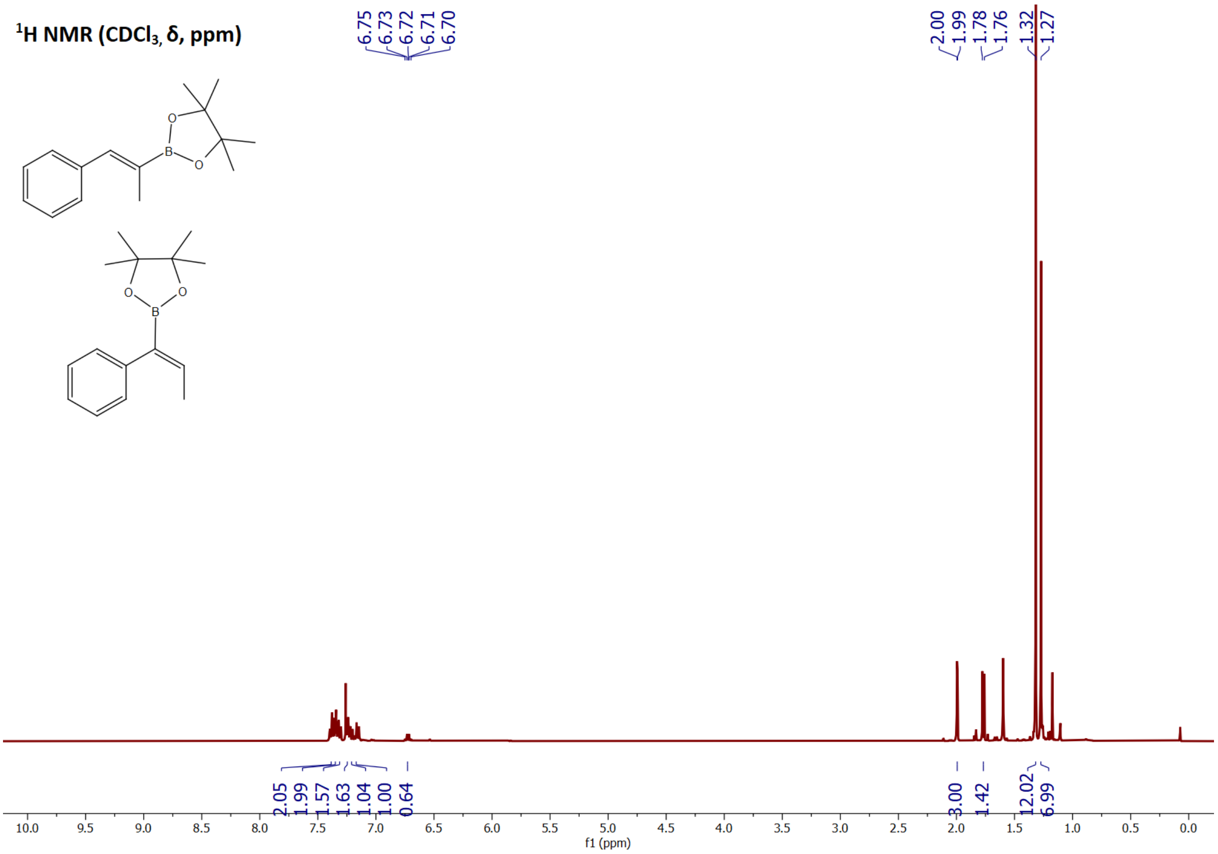

Figure 44. ^{1}H NMR (400 MHz, δ, $CDCl_3$) of mixture of **4c and 4c′**

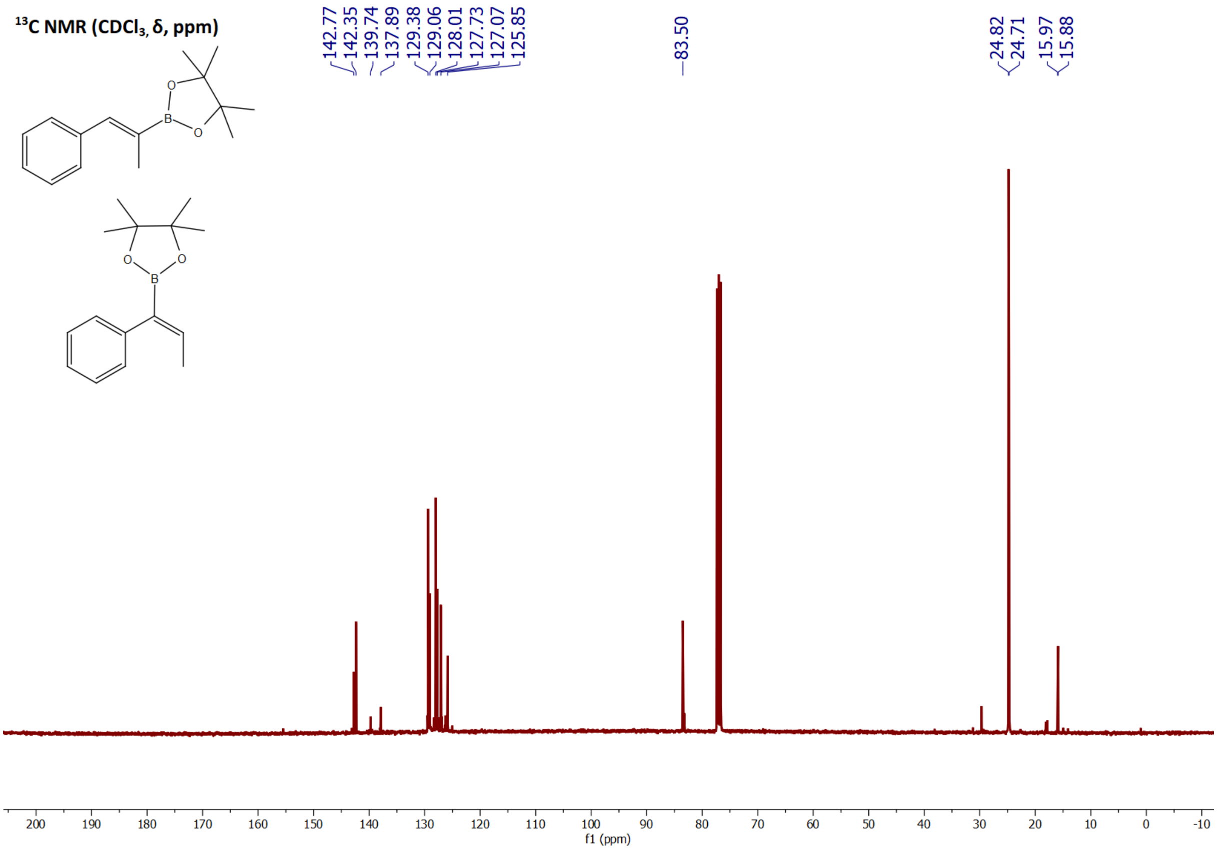

Figure 45. ^{13}C NMR (75MHz, δ, $CDCl_3$) of mixture of **4c and 4c′**

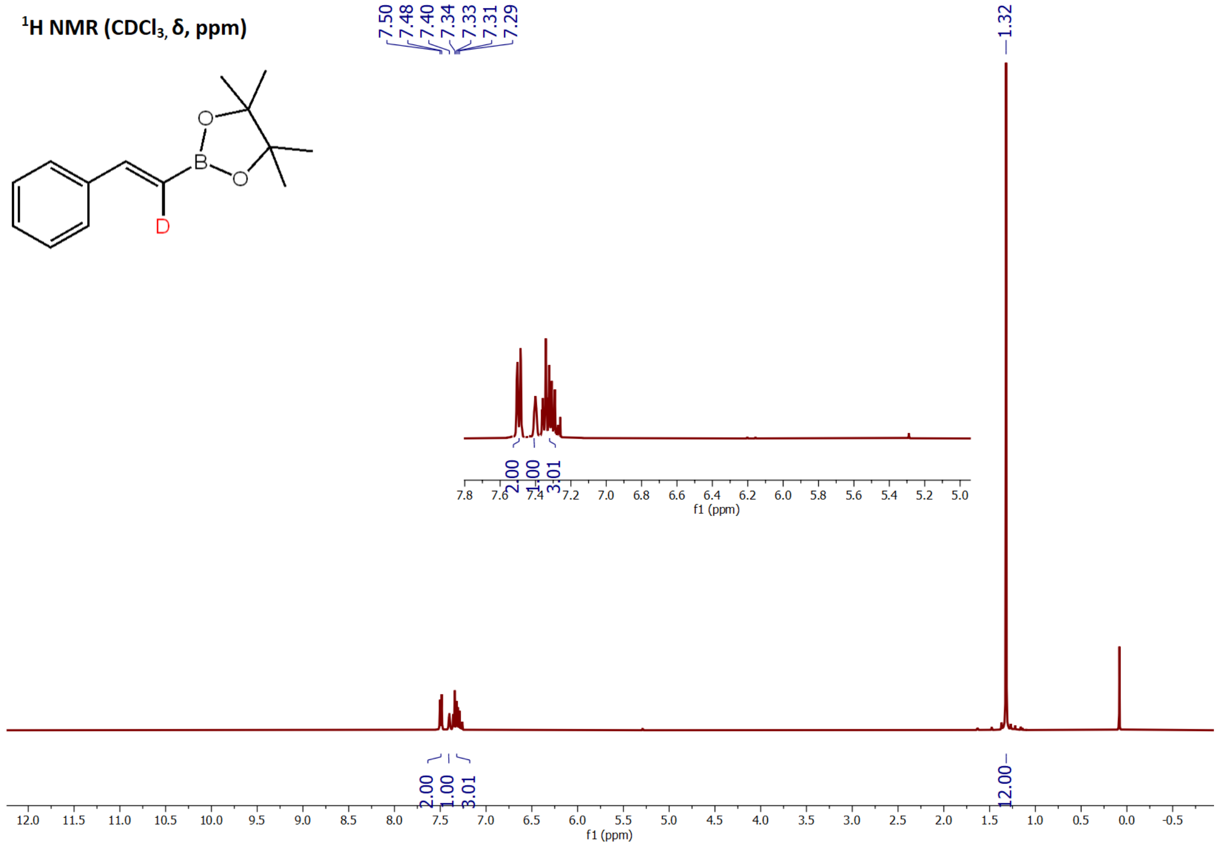

Figure 46. ^{1}H NMR (400 MHz, δ, $CDCl_3$) of **3a'**

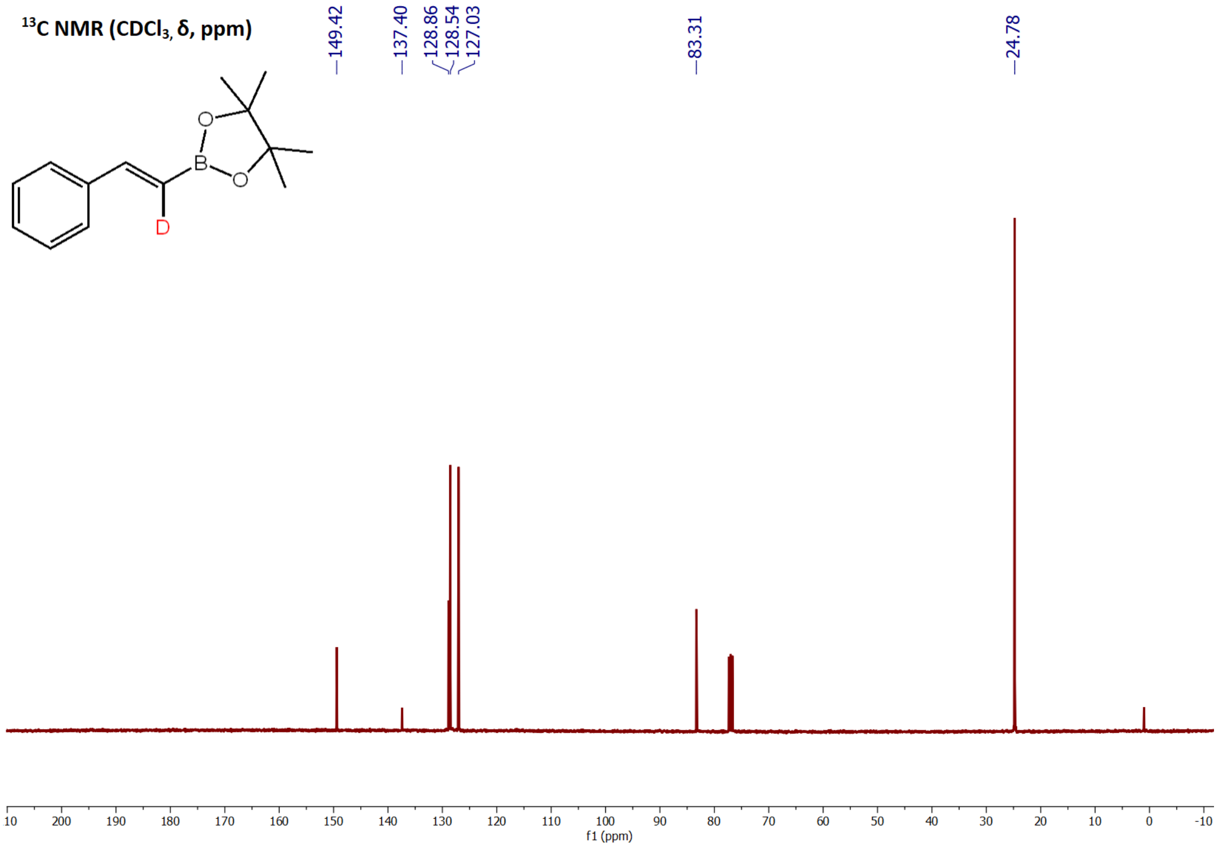

Figure 47. ^{13}C NMR (75MHz, δ, $CDCl_3$) of **3a'**

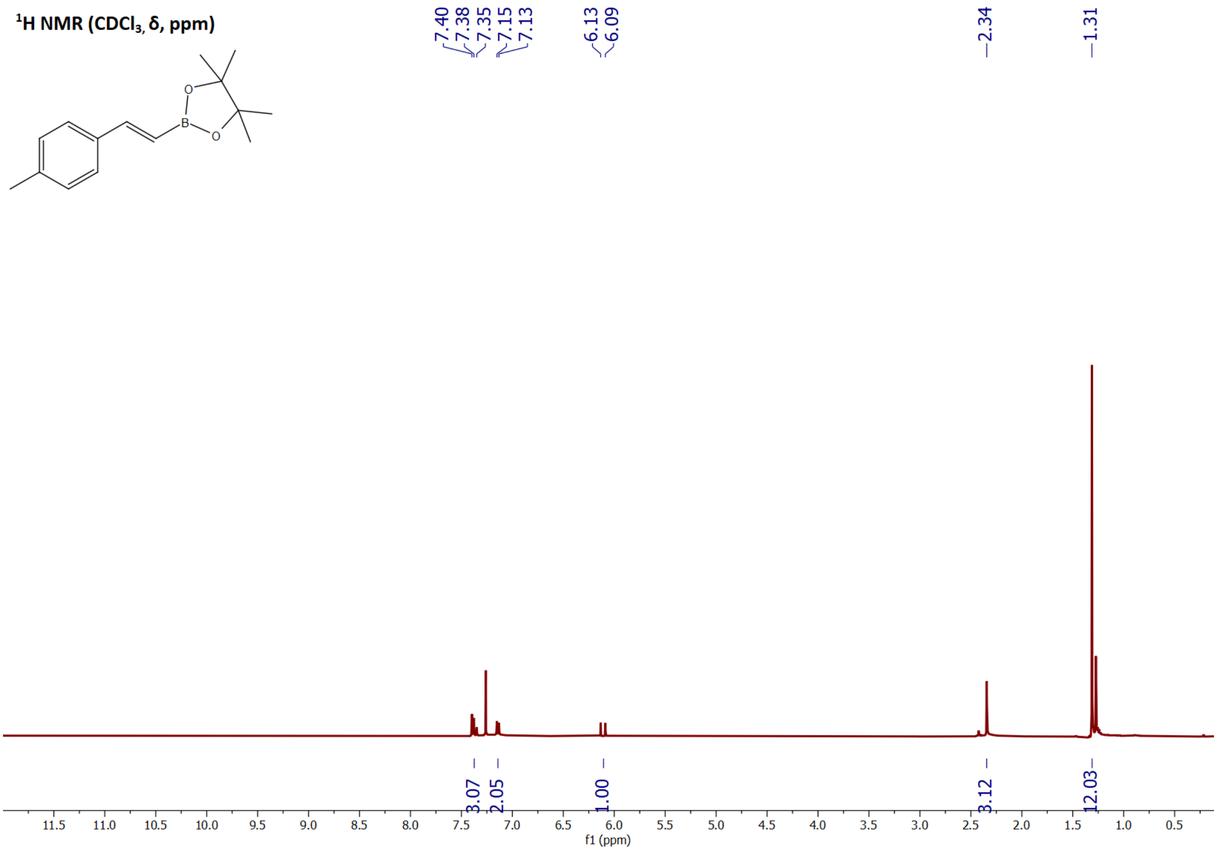

Figure 48. ^{1}H NMR (400 MHz, δ, CDCl$_3$) of product **3a** obtained in mercury poisoning test

5. References

[S1] M. Haberberger, S. Enthaler *Chem. Asian J.*, 2013, **8**, 50-54.

[S2] G. Zhang, S. Li, J. Wu, H. Zeng, Z. Mo, K. Davis, S. Zheng *Org. Chem. Front.*, 2019, **6**, 3228-3233

[S3] D. Ojha, K. Prabhu *Org. Lett.* 2016, **18**, 432–435

[S4] J. Zhao, Z. Niu, H. Fu, Y. Li *Chem. Commun.* 2014, **50**, 2058-2060

[S5] H. Ho, N. Asao, Y. Yamamoto, T. Jin *Org. Lett.* 2014, **16**, 4670–4673

[S6] W. J. Jang, B.-N. Kang, J. H. Lee, Y. M. Choi, C.-H. Kim, J. Yun *Org. Biomol. Chem.* 2019, **17**, 5249-5252

[S7] R. Mamidala, V. K. Pandey, A. Rit, *Chem. Commun.* 2019, **55**, 989-992

[S8] X. Shi, S. Li, L. Wu, *Angew. Chem. Int. Ed.* 2019, **58**, 16167

[S9] V. S. Rawat, B. Sreedhar, *Synlett* 2014, **25**, 1132-1136

[S10] S. Mandal,S. Mandal, K. Geetharani, *Chem. Asian J.* 2019, **14**, 4553 –4556

[S11] J. Altarejos, D. Sucunza, J.J. Vaquero, J. Carreras, *Eur. J. Org. Chem.* 2020, 3024-3029

[S12] S. Chen, L. Yang, D. Yi, Q. Fu, Z. Zhang, W. Liang, Q. Zhang, J. Jia, W. Wei, *RSC Adv.* 2017, **7**, 26070-26073
